# A maximum surgical blood ordering schedule: Does it add value?

**DOI:** 10.1111/vox.13804

**Published:** 2025-02-25

**Authors:** Deborah L. Benzil, Moises Auron, Zaher K. Otrock, Daniel Lallo, Noreen Flowers, Kenneth Cummings, NurJehan Quraishy, Deborah Tolich

**Affiliations:** ^1^ Department of Neurosurgery Neurological Institute, Cleveland Clinic Cleveland Ohio USA; ^2^ Department of Hospital Medicine Integrated Hospital Care Institute, Cleveland Clinic Cleveland Ohio USA; ^3^ Case Western Reserve University School of Medicine Cleveland Ohio USA; ^4^ Outcomes Research Consortium® Cleveland Ohio USA; ^5^ Transfusion Medicine, Division of Laboratory Medicine Diagnostic Institute, Cleveland Clinic Cleveland Ohio USA; ^6^ Department of Laboratory Medicine Diagnostic Institute, Cleveland Clinic Cleveland Ohio USA; ^7^ OR Operations, Cleveland Clinic Cleveland Ohio USA; ^8^ Center for Perioperative Medicine, Integrated Hospital Care Institute, Cleveland Clinic Cleveland Ohio USA; ^9^ University of Toledo Medical Center Cleveland Ohio USA

**Keywords:** cost savings, high‐value care, MSBOS (maximum surgical blood ordering system), patient safety, type and screen

## Abstract

**Background and Objectives:**

Implementing and complying with a maximum surgical blood ordering schedule (MSBOS) is challenging but essential to avoid waste and reduce costs. MSBOS helps manage blood product scarcity and healthcare expenditure by avoiding unnecessary pre‐transfusion testing and preparation, reducing product waste and improving clinical and operational efficiencies while maintaining patient safety.

**Materials and Methods:**

A multi‐hospital health system in Ohio and Florida performing more than 200,000 surgeries annually implemented MSBOS through a risk‐stratified protocol and electronic medical record automation.

**Results:**

The first‐year analysis included 107,149 cases in 23 surgical specialties and 18 hospitals. Compliance with MSBOS improved over time, reducing type and screen tests by 4166 and saving $223,839 in costs. No patient safety issues were identified.

**Conclusion:**

This project demonstrates that adopting MSBOS in a large health system adds value by reducing unnecessary testing and costs while maintaining patient safety.

**Highlights**

- A risk‐stratified protocol and stakeholder engagement are necessary to develop a maximum surgical blood ordering schedule (MSBOS).
- Electronic medical record automation assists in MSBOS adherence.
- Continuous improvement strategies are valuable to improve MSBOS adherence.

## INTRODUCTION

Effective implementation and compliance with a maximum surgical blood ordering schedule (MSBOS) poses significant challenges, despite its known benefits to avoid waste and reduce costs. This problem is further aggravated in large healthcare systems spanning multiple hospitals that may have variable traditions, cooperation, trust and levels of patient acuity. However, given the high cost of healthcare in the United States, such programmes are essential to reduce waste and costs. Shifts towards a high‐value approach to blood transfusions are a necessity given the increased healthcare expenditure without demonstrable impact in healthcare outcomes and the increasing scarcity of available blood products despite a constant demand [1].

Traditionally, anaesthesia and surgical teams have required that blood products be available prior to the start of many surgical procedures [2]. An increasing body of evidence suggests that many surgeries do not require a type and screen (TSCR) or type and cross‐match (TC), which adds cost and impacts the availability of blood which has become a stressed resource [3, 4]. Hospital blood banks prepare blood products for the operating room only to retrieve the vast majority as not being transfused at the end of the day. Studies have shown that more than 70% of red blood cell (RBC) units prepared were returned to the blood bank unused, resulting in an elevated cross‐match to transfusion (C/T) ratio [5]. In the periprocedural environment, appropriate MSBOS implementation can determine the need for blood TSCR testing as well as predict the need for RBC units with significant (40%) reduction in perioperative blood product orders and reduction in costs, with no demonstrable harm to patients [4]. Establishing an MSBOS allows aligning orders for cross‐matched blood and the potential transfusion needs of a patient, serving as guidelines for surgeons, anaesthesiologists and transfusion services, without interfering with clinical judgement. The successful implementation of MSBOS has been proven to reduce blood product waste, minimize the number of cross‐matched units, improve blood availability, save costs and enhance clinical and operational efficiencies while ensuring that patient safety is not compromised [6]. However, just having a generic MSBOS in place may not optimize waste elimination. In one study, even after MSBOS, analysis of more than 1000 surgical procedures revealed 71% of units issued for 215 cases were returned unused. This suggests that an institution‐specific, data‐driven MSBOS may be required to achieve full benefit of cost reduction and patient safety [5]. Surgeon and anaesthesiologist traditions seem particularly hard‐wired, and it requires a change in culture for the implementation of something like an MSBOS programme. The increasing pressure that all healthcare systems face for improved productivity and reduced costs only aggravates this challenge if not made easy to implement.

A 17‐hospital health system in Ohio and Florida performing more than 200,000 surgeries and procedures annually had not yet implemented MSBOS in 2020 for surgical patients. Out of all surgeries conducted, 65% had a TSCR test routinely performed, leading to inefficiency from unnecessary testing, allocation and issuing of unnecessary units. A project was launched in June 2021, and clinical assistance was sought from anaesthesiology and surgical department chairs. The objective was to eliminate 90% of unnecessary TSCR testing through an automated, risk‐stratified protocol with electronic medical record (EMR) prompts to clinicians. The initial implementation did not include recommendations for cross‐matched units to focus solely on adherence of TSCR testing. The MSBOS document is available in File S1. The project plan included tracking compliance post implementation, as well as monitoring any unforeseen impacts. This paper describes the significant challenges associated with this improvement effort and highlights strategies that can help others with similar endeavours.

## MATERIALS AND METHODS

### Healthcare system

The healthcare system spans Ohio and Florida with 17 hospitals when the project began (the 18th hospital opened during the first year of MSBOS implementation). Many of the hospitals are tertiary or quaternary centres doing the most complex cases including a high proportion of cardiac, thoracic, neurosurgical and urological procedures. Trauma is also a major component of the care provided. Most of the >200,000 surgeries performed annually are on adults. The proportion of surgical patients who require hospital admission is higher than the national average given the case complexity.

### Target population and context

Surgical procedures included in the development of an MSBOS were those performed in an operating room with at least 25 cases per year. Paediatric, obstetric, emergency procedures and patients with history of or current positive antibody screen were excluded. The focus was elective surgical procedures with a risk of transfusion.

### Interventions

#### Phased approach to MSBOS implementation and analysis

An A3 project to operationalize implementation and analysis of an MSBOS programme across all hospitals within a large health system was initiated early in 2021. A3 is a lean methodology tool that allows a stepwise assessment and problem solving [6, 7]. The project included the following phases: A3 team creation and project initiation, data collection and review, MSBOS drafting and endorsement, MSBOS implementation and analysis of impact. Details of each component are outlined.

#### A3 team creation and project initiation

Surgical services leadership was tasked with creating a team that could complete an A3 project, based on standard quality improvement/lean problem solving representing surgery, anaesthesiology, laboratory and blood bank and data analytics. Support from legal and ethics teams was used ad hoc (see Appendix S1 for full list of contributors). Following extensive discussions and review of baseline data, a SMART (Specific, Measurable, Assignable, Realistic and Time‐bound) goal was established to *Eliminate 90% of unnecessary Type and Screen/Confirm ABO tests through an automated, risk‐stratified protocol and automated electronic medical record automation*. Specifics included the following:

1. Develop a consensus risk stratification for the most common procedures performed across the enterprise;
2. Track compliance, reduction of tests and associated cost savings;
3. Track complications resulting from *not* having a TSCR in a patient who required transfusion perioperatively.

A detailed plan was worked out that involved data collection to achieve coverage of 75% of all surgical cases, establishment of key demographic data (case, transfusion rate, C/T index, average case‐specific blood loss), drafting of MSBOS through simultaneous application of professional expertise (an anaesthesiologist and surgeon pair from each specialty and facility) and based on literature‐supported data, consensus development of MSBOS, EMR automation, launch, health system communication, then audit for compliance, patient safety and assessment of financial impact (see Appendix S2 for full A3).

Data sourced from the anaesthesia information management system were retrieved for a 6‐month interval using current procedural terminology (CPT®) codes including the descriptions. Additional data included service line, average estimated blood loss (EBL), number of procedures/transfused RBCs in the operating room, total number of procedures and total number of RBCs transfused.

#### Health‐system‐specific MSBOS development

As part of the change management strategy, surgical specialists and anaesthesiologists were consulted to complete a spreadsheet indicating whether a TSCR or TC was needed based on CPT and procedure name. They had further opportunity to specifically indicate the need for type and cross or type and hold (and how many units) as well as special requirements related to patient features. These designations relied on historical patterns and drivers, rather than actual likely blood loss, current adjuncts to surgery, change in surgical approaches and advances in anaesthesia.

Based on the literature, thresholds were established for system‐specific MSBOS using transfusion indices (5% or more of each CPT transfused, average transfusion per patient of 0.3 and above) [4]. Given the variations between hospital locations, the indices were compared between hospitals and found to be consistent, which led to the adoption of a universal MSBOS for the health system. This determination was based on the confidence that electronic cross‐matching and emergency release is available at all hospital locations, thereby providing the ability for rapid release of blood products. There was significant challenge in obtaining reliable and meaningful blood loss data given known inaccuracy of operative reports, differences in automation of anaesthesia records as well as determination of specific data required.

The final phase involved consensus development of MSBOS for each specialty based on discussion of all cases where the professional expertise and data analysis suggested different recommendations because there was significant misalignment between the collaborating teams and the MSBOS recommendations based on the literature. Two team members (surgery and transfusion services) reviewed all cases and noted suggestions based on last 2‐year experience and added to spreadsheet. Additional transfusion rates were also added to the spreadsheet (two measures: transfusion/case and units transfused/case). The data were shared with clinical teams to ensure transparency, and the feedback provided subsequently led to adjustments in the MSBOS protocol.

The final consensus was shared with all surgeons in each specialty for comments prior to finalizing the MSBOS schedule.

#### Study of the interventions, measures and statistical analysis

The primary outcome measure was to determine whether the project goal was met (85% compliance). Statistics were gathered using an internal analytics database to determine compliance by hospital and institute between May 2023 and May 2024. Compliant (green) was assigned for 85%, nearly compliant (yellow) for 70%–84% and non‐compliant (red) for <70%. Compliance data were compared between hospitals, institutes and between the first 6 months and the last 6 months.

Other important measures included assessing the utility of the automated electronic medical record order process. In addition, data on compliance were considered important with attention to individual specialty and hospital differences, if discovered. Data on patient safety and impact on case delays were collected as additional important factors. Finally, cost and waste reduction were measured.

Most of the statistics reported are descriptive, including compliance (global, specialty‐ and hospital‐specific, and across different time intervals), patient safety, utility of the automated electronic medical record order and total cases analysed. For comparison of emergency release of blood products and case delays, chi‐square analysis was performed with significance set at *p* < 0.05. Cost reduction was calculated using just the charges associated with performance of a TSCR at the main hospital in the system. Four of the 17 hospitals were not on the same EMR platform before launch and therefore pre‐implementation data was not available to use as a baseline.

#### MSBOS launch

Data needed to determine the frequency of transfusion, the total volume of each surgical procedure code and the number of RBC units administered for each surgical procedure code were extracted from anaesthesia records contained in the enterprise data vault via complex Sequel software query. This query was used as the data source to develop an MSBOS Tableau (Seattle, WA: Tableau Software) dashboard that clearly displayed the transfusion frequencies and number of RBC units transfused per surgical procedure code. Additionally, the dashboard identified surgical procedures that qualified for a preoperative TSCR according to MSBOS criteria and those that did not. A second dashboard was developed to identify discrete surgical procedures for which testing was habitually ordered but never required the transfusion of RBCs. Additionally, the dashboards highlighted surgical procedures that consistently transfused RBCs and would always require a TSCR to be performed prior to surgery.

The MSBOS was launched in April 2023 before the EMR alert (October 2023) to give providers time to adjust. The initial schedule release surfaced additional misalignment, which prompted collaboration meetings and modifications to the MSBOS based on end‐user feedback.

### Ethics statement

No ethical issues were raised after a high‐level review of the proposed A3 project by our ethics team.

## RESULTS

### Global findings

Our first‐year analysis included 107,149 cases in 23 surgical specialties and 18 hospitals. In the large healthcare system studied, implementation of a singular process uncovered some important roadblocks. The first was that there was no standard process for ordering TSCR in the perioperative management of patients, and therefore addressing non‐compliance was complex and challenging. Additional waste was also seen in processes historically deployed to confirm blood product availability for a large proportion of cases (many of which did not require such under an even conservative MSBOS programme). Other procedural sectors were keen to implement similar programmes once the primary MSBOS was launched, requesting even more comprehensive programmes to reduce unnecessary perioperative testing. The limitation of using CPT codes was also uncovered, as these frequently may cover cases that both would and would not require TSCR (e.g., 1–2 level lumbar fusions vs. 3–4 level lumbar fusion).

### Primary outcome analysis: Compliance

The goal was 85% compliance with the MSBOS recommendations. During the first 6 months, there were 96 time points measured over 16 hospitals. Of these, just 2 were compliant (2%), 54 (56%) were nearly compliant and 40 (42%) were non‐compliant. During the last 6 months, there were 102 analysed intervals over 17 hospitals, with 4 compliant (4%), 67 nearly compliant (66%) and 31 non‐compliant (30%) (see Table 1). Similar trends were seen when comparing the institutes, with just one nearly compliant institute throughout the first 6 months and then two to three nearly compliant during the remaining analysis (see Table 1). These results did not reach statistical significance at the 0.05 significance level.

**TABLE 1:** Maximum surgical blood ordering system compliance by hospitals and institutes.

Entity	First 6 months^a^			Last 6 months^b^		
	Compliant	Nearly compliant	Non‐compliant	Compliant	Nearly compliant	Non‐compliant
Hospital	2	54	40	4	67	32
Institutes	0	6	24	0	13	17

*Note*: Hospitals include multiple institutes. Institutes are groupings of similar specialties, that is, digestive disease or women's health.

^a^ Hospital *N* = 16, Institute *N* = 5.

^b^ Hospital *N* = 17, Institute *N* = 5.

**FIGURE 1:**
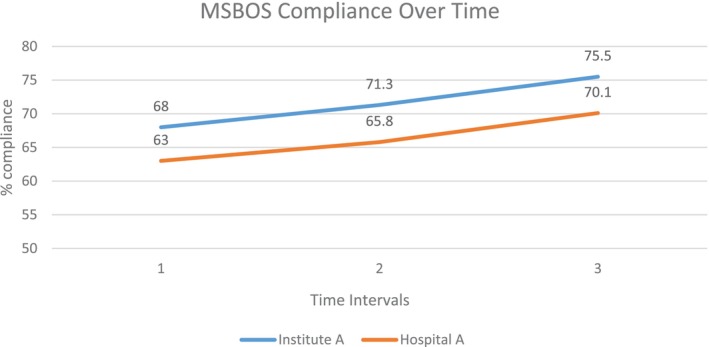
Maximum surgical blood ordering schedule (MSBOS) compliance over time. Time intervals are post implementation: 1 = 3 months, 2 = 6 months and 3 = 9 months. Representation of one hospital location and institute or a grouping of similar service lines.

**FIGURE 2:**
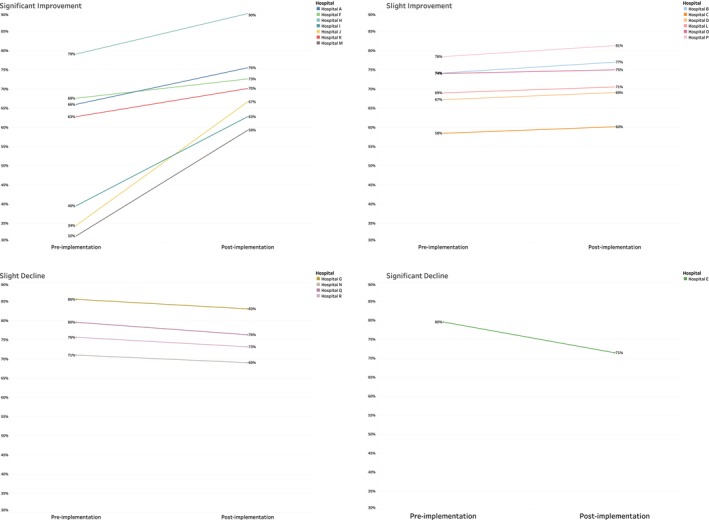
Each hospital location, represented by the degree of change between pre implementation and post implementation.

Every hospital and institute showed at least some overall increased compliance over time (see Figure 1 for representative example). Compliance was highly variable by specialty, hospital and over time (Figure 2 shows representation of hospital variation and over time). Thirteen hospitals (72%) showed overall improvement, whereas 7 hospitals out of 13 demonstrated significant improvement. It is noted that those with the poorest pre‐implementation performance had the most improvement. The hospitals that experienced a decrease post implementation were initially at the high end of adherence. Only one hospital location had a significant decline.

### Test reduction and cost savings

Five hospital sites did not have baseline data available prior to implementation; for these locations, a baseline was calculated using the data available for the first 3 months. During the initial 3 months, 8/13 (62%) hospitals that had baseline data available showed a decrease in testing, leading to a total of 698 fewer tests. In months 4 through 6, 15/17 (88%) hospitals showed reductions of 1453 TSCR tests. Months 7 through 9 included all 18 hospital locations for which we had data for comparison, and all but 2 hospitals (16/18; 89%) showed decreases of TSCR tests (*N* = 2015). The total reduction of TSCR tests at 9 months was 4166.

Table 2 shows the impact of the reduction of TSCR tests and the reduction in cost realized. The cost of a TSCR test varies by location; therefore, the hospital with the largest volume and one of the lowest costs per test ($53.73) was used to calculate a conservative cost avoidance. Over the first 9 months of implementation, the total cost savings was at least $223,839.

**TABLE 2:** Impact of a maximum surgical blood ordering system on ordering and cost of type and cross‐match.

Hospital	TSCR 9‐month test reduction	Cost avoidance
A	440	$23,641
B	151	$8113
C	173	$9295
D	158	$8489
E	55	$2955
F	283	$15,206
G	1698	$91,234
H	−27	−$1451
I	319	$17,140
J	−5	−$269
K	176	$9456
L	153	$8221
M	31	$1666
N	407	$21,868
O	−27	−$1451
P	87	$4675
Q	107	$5749
R	−13	−$698
Totals	4166	$223,839

*Note*: The cost per TSCR is defined as $53.73. Variance from baseline = subtraction of post‐TSCR percentage from before implementation.

$\text{Percentage difference}=\frac{\left|V_{1}-V_{2}\right|}{\left(V_{1}+V_{2}\right)/2}\times 100.$

Abbreviation: TSCR, type and screen.

### Patient safety

Post implementation, there were no reported patient safety issues related to the MSBOS and delays due to antibody detection. Emergency release volumes remained unchanged (pre 6/33,640 or 0.0001%, post 8/73,509 or 0.0001%), and this change was not significant (*p* = 0.355). Surgical delays due to no TSCR before implementation were 15 cases (0.026%), of which 55% would not have required one by the MSBOS schedule. Post implementation, 21 cases (0.029%) were delayed, of which 40% did not require a TSCR according to the MSBOS (*p* = 0.184).

### Utility of automated electronic medical record order

**FIGURE 3:**
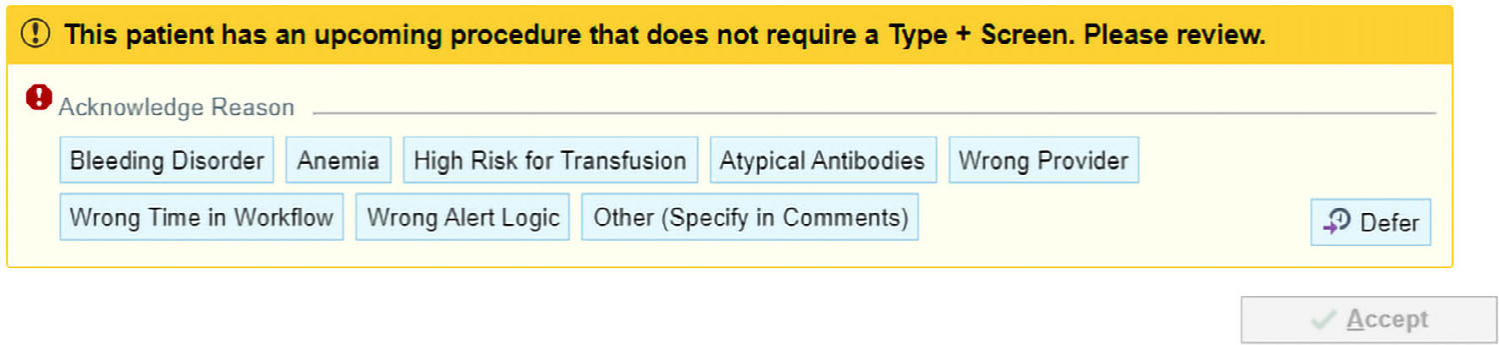
Best practice alert. Alert fires when a patient is scheduled for a procedure that does not require a type and screen. The ordering, the provider may override by indicating a reason to continue ordering the test. Wrong provider, wrong time in workflow and wrong alert logic are standard reasons for override.

**FIGURE 4:**
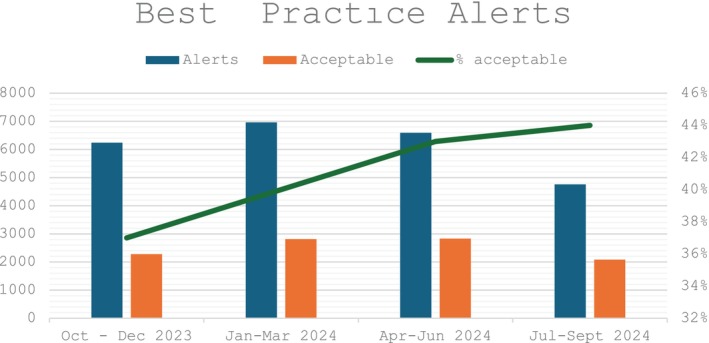
Best practice alert overrides. Alerts = number of alerts fired; acceptable = number for reasons for bleeding disorder, anaemia, high risk for transfusion, and atypical antibodies; percentage acceptable of total alerts.

The EMR best practice alert (Figure 3) was implemented 6 months after the introduction of MSBOS. In the first quarter post implementation, 6240 alerts were generated, of which 37% were overridden due to acceptable exceptions such as the risk of transfusion, positive antibody screen or anaemia and in 11% (708) the order was cancelled. Subsequent quarters showed improvement with the exception that between 6% and 7% of orders were cancelled (Figure 4). In the fourth quarter after initiation, alerts decreased to 4759 with 44% deemed acceptable.

## DISCUSSION

Continuous improvement (CI) strategies should support efforts within healthcare to reduce waste, increase value and enhance patient safety and provider engagement. It can be particularly useful in supporting change management and addressing traditional but unsupported workflows. The uncertainty about bleeding risk, as well as the aim for maximum patient safety, has led to overestimating the need for perioperative blood product testing. This has led to the over‐release of blood products by blood banks and considerable waste of resources that are in short supply and high demand [5]. Thus, MSBOS offers the opportunity to apply CI strategies to effect meaningful change [8]. Using a CI process supported the effective implementation of a systems‐wide MSBOS programme despite significant resistance. The A3 improvement process was effective, but it has not yet allowed the achievement of our primary outcome of 90% reduction in unnecessary testing. Our findings confirm that it is safe and effective to establish MSBOS across a large and varied health system, but achieving full compliance remains a challenge. Financial savings were found to be considerable despite full compliance not being realized. Patterns of compliance suggested opportunities for targeted improvement efforts.

Despite not reaching our primary outcome goal, the improvement project has strengths that are worth noting. First, the process itself prompted collaboration and compromise. Involvement of the surgeons and anaesthesiologists was invaluable for implementation and establishing teams to address ongoing concerns and compliance. Analysis of components beyond cost savings and reduction of waste are also important, as all CI efforts must take care to address the quadruple aim of healthcare of not sacrificing one for the sake of another. Further enhancing the utility of this project will require a long‐term assessment of safety and effectiveness. Ongoing efforts to drive compliance with outliers (specialties or hospitals) are also required. Analysis of the patterns of compliance have allowed us to tailor ongoing interventions to drive these additional efforts. Finally, the need for additional work has been exposed in settings in which the MSBOS recommendations are implemented without any clinical judgement. The lack of a good coding system to group cases is a problem not just for our project but for any hospital system considering implementation of MSBOS. International Classification of Diseases‐10th revision, CPT and diagnosis‐related group are all inadequate in optimally establishing MSBOS in a large‐volume setting.

While our programme found significant limitations related to the use of CPT for establishing an MSBOS programme, others have noted success. One study reported the impact of a hospital in Australia that used CPT codes and the probability of blood transfusion as significant in reducing perioperative testing and, in some surgeries, the rate of transfusions [9]. One advantage of using CPT in a large health system is that it allows easy identification of our high‐volume procedures; it also helped in overcoming the challenges and limitations associated with the different electronic data sources. A challenge for developing and analysing local MSBOS protocols can be the lack of optimal electronic health record (EHR) technology. Despite significant advances over time, most hospitals have multiple systems that document aspects of patient care and the associated costs. Anaesthesia records were frequently separate from other components of the EHR, while reliable calculation of operative blood loss remains unattainable. Our A3 effort identified these challenges and worked to find surrogate measures when reliable data was not available. Ultimately, the lack of data did not impact the final MSBOS recommendations but would have been impactful in discussions with recalcitrant surgeons and anaesthesiologists who could be swayed by rare but memorable events.

In addition to achieving compliance, sustainability will be important. At least one study clearly demonstrated that once implemented, there is sustained cultural change, even after 10 years [10]. Our study showed that implementation of MSBOS results in challenges and resistance to change in practice. There were no patient safety issues, nor were there changes in the rate of emergency release volumes or associated surgical delays. These outcomes contribute to enhanced buy‐in and reinforce best practices. The incorporation of MSBOS into the EHR with best practice alerts, which allows the awareness of the lack of necessity for cross‐matching, helps in reinforcing compliance. Post implementation, we did see enhanced compliance over time with increased cost savings. The cost savings can be further expanded once the adoption and hard‐wiring of the MSBOS process becomes more standardized and disseminated. Further refinement of the MSBOS process can occur by data surveillance with careful attention to clinical outcomes. Also, the efficiency associated with decreased cross‐matching of RBC units facilitates a more efficient management of the blood bank's inventory and decreases waste in unnecessary processes.

Our study has the strength of implementation in a large and varied healthcare system, which allows obtaining robust data from its conclusions and suggests that the data are generalizable in many settings. However, it has several limitations. It is a single healthcare organization, which has numerous hospitals, with several being incorporated within the past 20 years, so adoption of harmonization of best practices within the system is not uniform. The recent implementation of EHR in some hospitals requires training and incorporation into the local physicians' workflow. As noted, some data were challenging to obtain and impacts our ability to fully analyse our current and future data with past data. The launch of MSBOS required redundant communication methods, which may not be replicable in smaller settings, along with a default Yes or No for the TSCR order based on the CPT. The lack of standard process for TSCR or TSCR30 (type and screen 30‐day) ordering was another potential limitation of analysis of the CI process success. As with all MSBOS programmes, the recommendations are based on historical transfusion rates associated with specific CPT codes. A recent study showed that incorporating the patient's haemoglobin in the MSBOS equation can help predict a more accurate estimation of blood requirements [11]. Finally, the long‐term sustainability and potential for enhanced compliance are fundamental goals on which we are keeping a close watch to learn and implement potential new iterations in the process. Our project compares with those of other multi‐hospital systems, in which data‐driven MSBOS leads to decrease in blood ordering practices [12]. Advances in surgical practices, including minimally invasive and robotic procedures, the use of new haemostatic agents and the lowered thresholds for transfusion, support the importance of MSBOS; also, the development of a MSBOS is not a single event but a process that requires regular updating.

Looking into the future, artificial intelligence and machine learning algorithms can allow the study and evaluation of multiple variables to provide refined and accurate predictions on the needs for blood transfusions [13].

In conclusion, MSBOS was effective in reducing the extent of unnecessary pre‐transfusion testing before surgery and reduced the number of RBCs that were cross‐matched for specific patients, apart from contribution to significant healthcare costs saving. Any health system undertaking implementation of am MSBOS programme because of its known benefits should consider utilization of a CI process, such as an A3, to effect a meaningful and safe outcome.

## CONFLICT OF INTEREST STATEMENT

The authors declare no conflicts of interest.

## Supporting information


**Data S1.** Supporting information.

**Data S2.** Supporting information.

**Data S3.** Supporting information.

## Data Availability

The data that support the findings of this study are available on request from the corresponding author. The data are not publicly available due to privacy or ethical restrictions.
