# Supplementary material for: A maximum surgical blood ordering schedule: Does it add value?
Source: Vox Sang. 2025 Feb 25;120(4):411–8. doi: 10.1111/vox.13804 (PMC12017947; doi:10.1111/vox.13804)
Supplement: Supplementary file 1 — Data S1. Supporting information. [file VOX-120-411-s003.docx]

Appendix 1

**List of Project Contributors:**

**Sponsor:** Dr. Mark Taylor
**Process Owners:** Drs. Deborah Benzil and NurJehan Quraishy
**Project Lead**: Dr. Debbie Tolich
**Team:** Ralph Luther, Dr. Ken Cummings, Dr. Moises Auron, Dr. Zaher Otrock, Dan Lallo, Jessica Calo, Michael Reese, Noreen Flowers

**Data Collection, Dashboards:** Mike Sutton
**Ethics:** Cristie Cole, Jane Jankowski **Legal:** Caitlan Grombka-Murphy, Amy Cooper
**ITD:** Jack Stock
