# Supplementary material for: A maximum surgical blood ordering schedule: Does it add value?
Source: Vox Sang. 2025 Feb 25;120(4):411–8. doi: 10.1111/vox.13804 (PMC12017947; doi:10.1111/vox.13804)
Supplement: Supplementary file 2 — Data S2. Supporting information. [file VOX-120-411-s002.pdf]

MSBOS - All records

Individual patient parameters (i.e., anemia, coagulopathy, transfusion risk) should be taken into consideration and may warrant an individual TSCR when not generally recommended.

June 22, 2023 **Sorted by CPT**

Primary CPT	Procedure Name	TSCR (Type and Screen) Needed?
10060	ABSCESS I&D SIMPLE	No
10060	ABSCESS I&D SIMPLE	No
10060	INCISION & DRAINAGE ABSCESS EXTREMITY UPPER COMPLICATED/MULTIPLE	No
10060	INCISION & DRAINAGE CYST UPPER EXTREMITY SIMPLE/SINGLE	No
10060	INCISION & DRAINAGE OF ABSCESS EXTREMITY UPPER, SIMPLE OR SINGLE	No
10060	INCISION AND DRAINAGE ABCESS AXILLA SIMPLE/SINGLE	No
10060	INCISION AND DRAINAGE ABCESS FOOT AND HEEL	No
10060	INCISION AND DRAINAGE ABSCESS ABDOMEN SIMPLE OR SINGLE	No
10060	INCISION AND DRAINAGE ABSCESS BACK SIMPLE OR SINGLE	No
10060	INCISION AND DRAINAGE ABSCESS BUTTOCK	No
10060	INCISION AND DRAINAGE ABSCESS EXTREMITY LOWER SIMPLE OR SINGLE	No
10060	INCISION AND DRAINAGE ABSCESS FACE COMPLICATED OR MULTIPLE	No
10060	INCISION AND DRAINAGE ABSCESS FACE SIMPLE AND SINGLE	No
10060	INCISION AND DRAINAGE ABSCESS GROIN SIMPLE/SINGLE	No
10060	INCISION AND DRAINAGE ABSCESS TRUNK SIMPLE OR SINGLE	No
10060	INCISION AND DRAINAGE CYST GROIN SIMPLE/SINGLE	No
10061	I&D ABSCESS ON FACE	No
10061	INCISION AND DRAINAGE ABCESS AXILLA COMPLICATED/MULTIPLE	No
10061	INCISION AND DRAINAGE ABSCESS ABDOMEN, COMPLICATED OR MULTIPLE	No
10061	INCISION AND DRAINAGE ABSCESS BACK COMPLICATED OR MULTIPLE	No
10061	INCISION AND DRAINAGE ABSCESS BUTTOCKS COMPLICATED OR MULTIPLE	No
10061	INCISION AND DRAINAGE ABSCESS COMPLICATED OR MULTIPLE	No
10061	INCISION AND DRAINAGE ABSCESS EXTREMITY LOWER COMPLICATED OR MULTIPLE	No
10061	INCISION AND DRAINAGE ABSCESS GROIN COMPLEX MULTIPLE PEDIATRIC	No
10061	INCISION AND DRAINAGE ABSCESS GROIN COMPLEX/MULTIPLE	No
10061	INCISION AND DRAINAGE ABSCESS UPPER EXTREMITY, COMPLICATED OR MULTIPLE	No
10061	INCISION AND DRAINAGE BREAST ABSCESS COMPLICATED/MULTIPLE	No
10061	INCISION AND DRAINAGE COMPLEX ABSCESS OF NECK	No
10061	INCISION AND DRAINAGE OF ABSCESS TRUNK COMPLICATED OR MULTIPLE	No
10120	INCISE & REMOVE SUB-Q FOREIGN BODY FROM SCALP SIMPLE	No
10120	INCISION AND REMOVAL OF FOREIGN BODY BUTTOCK SUBCUTANEOUS TISSUE SIMPLE	No
10120	INCISION AND REMOVE FOREIGN BODY	No
10120	INCISION AND REMOVE FOREIGN BODY SUB-Q ABDOMEN	No
10120	INCISION AND REMOVE FOREIGN BODY SUB-Q HEAD / NECK	No
10120	INCISION AND REMOVE FOREIGN BODY SUB-Q LOWER EXTREMITY	No
10120	INCISION AND REMOVE FOREIGN BODY SUB-Q TRUNK	No
10120	INCISION AND REMOVE FOREIGN BODY SUB-Q UPPER EXTREMITY	No

MSBOS - All records

Individual patient parameters (i.e., anemia, coagulopathy, transfusion risk) should be taken into consideration and may warrant an individual TSCR when not generally recommended.

June 22, 2023 **Sorted by CPT**

Primary CPT	Procedure Name	TSCR (Type and Screen) Needed?
10120	REMOVAL FOREIGN BODY FACE	No
10120	REMOVAL FOREIGN BODY HAND	No
10120	REMOVAL FOREIGN BODY NECK ; SUB. CUT. TISSUE SIMPLE	No
10121	INCISION & REMOVAL OF FOREIGN BODY SUB-Q COMPLICATED	No
10121	REMOVAL FOREIGN BODY	No
10121	REMOVAL FOREIGN BODY EXTREMITY LOWER	No
10121	REMOVAL FOREIGN BODY EXTREMITY UPPER	No
10121	REMOVAL FOREIGN BODY EXTREMITY UPPER PEDIATRIC	No
10121	REMOVAL FOREIGN BODY NECK; SUB. CUT. TISSUE COMPLICATED	No
10121	REMOVAL FOREIGN BODY SUBCUTANEOUS ABDOMEN	No
10140	EVACUATION CLOT EXTREMITY LOWER	Yes
10140	EVACUATION CLOT EXTREMITY UPPER	Yes
10140	EVACUATION CLOT NECK	Yes
10140	EVACUATION HEMATOMA BREAST	Yes
10140	INCISION & DRAINAGE OF HEMATOMA	Yes
10140	INCISION & DRAINAGE OF HEMATOMA	Yes
10140	INCISION & DRAINAGE OF SEROMA	Yes
10140	INCISION AND DRAINAGE FLIUD COLLECTION GROIN	Yes
10140	INCISION AND DRAINAGE HEMATOMA GROIN	Yes
10140	INCISION AND DRAINAGE HEMATOMA SEROMA BREAST	Yes
10140	INCISION AND DRAINAGE OF HEMATOMA SEROMA OR FLUID COLLECTION BACK	Yes
10140	INCISION AND DRAINAGE OF HEMATOMA SEROMA OR FLUID COLLECTION BUTTOCKS	Yes
10140	INCISION AND DRAINAGE OF HEMATOMA SEROMA OR FLUID COLLECTION FACE	Yes
10140	INCISION AND DRAINAGE OF HEMATOMA SEROMA OR FLUID COLLECTION KNEE	Yes
10140	INCISION AND DRAINAGE OF HEMATOMA SEROMA OR FLUID COLLECTION LOWER EXTREMITY	Yes
10140	INCISION AND DRAINAGE OF HEMATOMA SEROMA OR FLUID COLLECTION UPPER EXTREMITY	Yes
10140	INCISION AND DRAINAGE OF HEMATOMA SKIN LOWER EXTREMITY	Yes
10140	INCISION AND DRAINAGE OF HEMATOMA SKIN OF BREAST	Yes
10140	INCISION AND DRAINAGE OF HEMATOMA SKIN UPPER EXTREMITY	Yes
10140	INCISION AND DRAINAGE OF HEMATOMA, SEROMA OR FLUID COLLECTION HIP	Yes
10140	INCISION AND DRAINAGE OF SEROMA OF ABDOMINAL WALL	Yes
10140	INCISION AND DRAINAGE SEROMA GROIN	Yes
10180	INCISION AND DRAINAGE POST OP INFECTION	No
10180	INCISION AND DRAINAGE POST OP INFECTION ABDOMEN / TRUNK	No
10180	INCISION AND DRAINAGE POST OP INFECTION ABDOMEN / TRUNK PEDIATRIC	No
10180	INCISION AND DRAINAGE POST OP INFECTION BACK	No
10180	INCISION AND DRAINAGE POST OP INFECTION BUTTOCKS	No

MSBOS - All records

Individual patient parameters (i.e., anemia, coagulopathy, transfusion risk) should be taken into consideration and may warrant an individual TSCR when not generally recommended.
June 22, 2023 **Sorted by CPT**

Primary CPT	Procedure Name	TSCR (Type and Screen) Needed?
10180	INCISION AND DRAINAGE POST OP INFECTION FOOT UNILATERAL	No
10180	INCISION AND DRAINAGE POST OP INFECTION FROM CRANIAL SURGERY HEAD	No
10180	INCISION AND DRAINAGE POST OP INFECTION FROM SPINAL SURGERY BACK	No
10180	INCISION AND DRAINAGE POST OP INFECTION GROIN	No
10180	INCISION AND DRAINAGE POST OP INFECTION HAND UNILATERAL	No
10180	INCISION AND DRAINAGE POST OP INFECTION LOWER EXTREMITY	No
10180	INCISION AND DRAINAGE POST OP INFECTION UPPER EXTREMITY	No
10180	INCISION AND DRAINAGE POSTOPERATIVE WOUND COMPLEX	No
11004	DEBRIDEMENT GENITALIA AND PERINEUM	Yes
11005	DEBRIDE ABD WALL: SKIN,SUBQ,MUSCLE&FASCIA NECROTIZING INFECT W/CLOSURE	No
11005	DEBRIDE ABD WALL: SKIN,SUBQ,MUSCLE&FASCIA NECROTIZING INFECT W/OCLOSURE	No
11005	DEBRIDEMENT OF SKIN SUBCUTANEOUS TISSUE MUSCLE AND FASCIA FOR NECROTIZING SOFT TISSUE INFECTION ABDOMINAL WALL WITH OR WITHOUT FASCIAL CLOSURE	No
11005	DEBRIDEMENT WOUND	No
11005	DEBRIDEMENT WOUND ABDOMEN	No
11005	INCISION AND DRAINAGE ABSCESS ABDOMEN WITH OR WITHOUT FASCIAL CLOSURE	No
11012	DEBRIDE SITE OPEN FRACTURE/DISLOCATION	Yes
11012	DEBRIDE FOREIGN MATTER SITE OPEN FRACTURE/DISLOCATION SKIN/SUBQ TISSUE/MUSCLE FASCIA/MUSCLE/BONE	Yes
11012	IRRIGATION AND DEBRIDEMENT OF OPEN FRACTURE AND/OR DISLOCATION; SKIN, SUBCUTANEOUS TISSUE, FASCIA, MUSCLE, AND BONE	Yes
11042	DEBRIDEMENT SUBCUTANEOUS TISSUE BUTTOCK FIRST 20 SQ CM	Yes
11042	DEBRIDEMENT SUBCUTANEOUS TISSUE FIRST 20 SQ CM OR LESS	Yes
11042	DEBRIDEMENT SUBCUTANEOUS TISSUE FIRST 20 SQ CM OR LESS	Yes
11042	DEBRIDEMENT SUBCUTANEOUS TISSUE FIRST 20 SQ CM OR LESS ABDOMINAL WALL	Yes
11042	DEBRIDEMENT SUBCUTANEOUS TISSUE FIRST 20 SQ CM OR LESS BREAST	Yes
11042	DEBRIDEMENT SUBCUTANEOUS TISSUE FIRST 20 SQ CM OR LESS CHEST	Yes
11042	DEBRIDEMENT SUBCUTANEOUS TISSUE FIRST 20 SQ CM OR LESS FACE	Yes
11042	DEBRIDEMENT SUBCUTANEOUS TISSUE FIRST 20 SQ CM OR LESS HEEL	Yes
11042	DEBRIDEMENT SUBCUTANEOUS TISSUE FIRST 20 SQ CM OR LESS LOWER EXTREMITY	Yes
11042	DEBRIDEMENT SUBCUTANEOUS TISSUE FIRST 20 SQ CM OR LESS SACRUM	Yes
11042	DEBRIDEMENT SUBCUTANEOUS TISSUE FIRST 20 SQ CM OR LESS UPPER EXTREMITY	Yes
11042	DEBRIDEMENT SUBCUTANEOUS TISSUE FIRST 20 SQCM OR LESS PERINEAL	Yes
11043	DEBRIDEMENT MUSCLE/FASCIA FIRST 20 SQ CM OR LESS BACK	Yes
11043	DEBRIDEMENT MUSCLE/FASCIA FIRST 20 SQ CM OR LESS LOWER EXTREMITY	Yes
11043	DEBRIDEMENT MUSCLE/FASCIA FIRST 20 SQ CM OR LESS SACRUM	Yes
11043	DEBRIDEMENT MUSCLE/FASCIAFIRST 20 SQ CM OR LESS	Yes
11043	DEBRIDEMENT MUSCLE/FASCIAFIRST 20 SQ CM OR LESS ABDOMEN	Yes

MSBOS - All records

Individual patient parameters (i.e., anemia, coagulopathy, transfusion risk) should be taken into consideration and may warrant an individual TSCR when not generally recommended.

June 22, 2023 **Sorted by CPT**

Primary CPT	Procedure Name	TSCR (Type and Screen) Needed?
11043	DEBRIDEMENT MUSCLE/FASCIAFIRST 20 SQ CM OR LESS ABDOMINAL WALL	Yes
11043	DEBRIDEMENT MUSCLE/FASCIAFIRST 20 SQ CM OR LESS CHEST	Yes
11043	DEBRIDEMENT MUSCLE/FASCIAFIRST 20 SQ CM OR LESS FOOT	Yes
11043	DEBRIDEMENT MUSCLE/FASCIAFIRST 20 SQ CM OR LESS HEAD	Yes
11043	DEBRIDEMENT MUSCLE/FASCIAFIRST 20 SQ CM OR LESS HIP	Yes
11043	DEBRIDEMENT MUSCLE/FASCIAFIRST 20 SQ CM OR LESS NECK	Yes
11043	DEBRIDEMENT MUSCLE/FASCIAFIRST 20 SQ CM OR LESS TRUNK	Yes
11043	DEBRIDEMENT MUSCLE/FASCIAFIRST 20 SQ CM OR LESS UPPER EXTREMITY	Yes
11043	DEBRIDEMENT SKIN SUBCUTANEOUS TISSUE, AND MUSCLE	Yes
11044	DEBRIDEMENT BONE W/ EPIDERMIS/DERMIS/ SUBCUTANEOUS TISSUE/ MUSCLE/FASCIA FIRST 20 SQ CM OR LESS	No
11402	EXCISION BENIGN CYST/MASS LESION ABDOMINAL 1.1 TO 2.0 CM	No
11402	EXCISION BENIGN CYST/MASS LESION AXILLA 1.1 TO 2.0 CM	No
11402	EXCISION BENIGN CYST/MASS LESION CHEST 1.1 TO 2.0 CM	No
11402	EXCISION BENIGN CYST/MASS LESION LOWER EXTREMITY 1.1 TO 2.0 CM	No
11402	EXCISION BENIGN CYST/MASS LESION UPPER EXTREMITY 1.1 TO 2.0 CM	No
11402	EXCISION BENIGN LESION 0.6 -> 1.0CM	No
11402	EXCISION BENIGN LESION ABDOMINAL 1.1 TO 2.0 CM	No
11402	EXCISION BENIGN LESION ARM 1.1 TO 2.0 CM	No
11402	EXCISION BENIGN LESION BACK 1.1 TO 2.0 CM	No
11402	EXCISION BENIGN LESION BREAST 1.1 TO 2.0 CM	No
11402	EXCISION BENIGN LESION BUTTOCK 1.1 TO 2.0 CM	No
11402	EXCISION BENIGN LESION CHEST 1.1 TO 2.0 CM	No
11402	EXCISION BENIGN LESION LEG 1.1 TO 2.0 CM	No
11402	EXCISION BENIGN LESION TRUNK 1.1 TO 2.0 CM	No
11403	BIOPSY LESION ABDOMEN	No
11403	BIOPSY LESION EXTREMITY UPPER	No
11403	EXCISION BENIGN CYST/MASS LESION 2.1-3.0CM	No
11403	EXCISION BENIGN CYST/MASS LESION AXILLA 2.1-3.0CM	No
11403	EXCISION BENIGN CYST/MASS LESION CHEST 2.1-3.0CM	No
11403	EXCISION BENIGN CYST/MASS LESION LOWER EXTREMITY 2.1-3.0CM	No
11403	EXCISION BENIGN CYST/MASS LESION UPPER EXTREMITY 2.1-3.0CM	No
11403	EXCISION BENIGN LESION BACK 2.1 TO 3.0CM	No
11403	EXCISION BENIGN LESION INCLUDING MARGINS EXCEPT SKIN TAG TRUNK ARMS OR LEGS EXCISED DIAMETER 2.1 TO 3.0 CM	No
11403	EXCISION BENIGN LESION NOT SKIN TAG ABDOMEN 2.1 -> 3.0CM	No
11403	EXCISION BENIGN LESION NOT SKIN TAG TRUNK 2.1 -> 3.0CM	No
11403	EXCISION CYST SEBACEOUS BACK	No
11403	EXCISION CYST SEBACEOUS CHEST	No

MSBOS - All records

Individual patient parameters (i.e., anemia, coagulopathy, transfusion risk) should be taken into consideration and may warrant an individual TSCR when not generally recommended.

June 22, 2023 **Sorted by CPT**

Primary CPT	Procedure Name	TSCR (Type and Screen) Needed?
11403	EXCISION CYST SEBACEOUS EXTREMITY LOWER	No
11403	EXCISION CYST SEBACEOUS EXTREMITY UPPER	No
11403	EXCISION LESION BACK	No
11403	EXCISION LESION EXTREMITY UPPER	No
11403	EXCISION NEVUS ABDOMEN	No
11403	EXCISION NEVUS BACK	No
11403	EXCISION NEVUS EXTREMITY LOWER	No
11403	EXCISION NEVUS EXTREMITY UPPER	No
11404	EXCISION BENIGN CYST/MASS LESION ABDOMEN 3.1-> 4.0CM	No
11404	EXCISION BENIGN CYST/MASS LESION CHEST 3.1-> 4.0CM	No
11404	EXCISION BENIGN CYST/MASS LESION LOWER EXTREMITY 3.1-> 4.0CM	No
11404	EXCISION BENIGN CYST/MASS LESION UPPER EXTREMITY 3.1-> 4.0CM	No
11404	EXCISION BENIGN LESION 3.1 TO 4.0 CM	No
11404	EXCISION BENIGN LESION BACK 3.1 TO 4.0 CM	No
11404	EXCISION BENIGN LESION BUTTOCK 3.1 TO 4.0 CM	No
11404	EXCISION BENIGN LESION NOT SKIN TAG ARMS 3.1 -> 4.0CM	No
11404	EXCISION BENIGN LESION NOT SKIN TAG TRUNK 3.1 -> 4.0CM	No
11404	EXCISION LESION GROIN	No
11406	EXCISION BENIGN CYST/MASS LESION ABDOMEN OVER 4CM	No
11406	EXCISION BENIGN CYST/MASS LESION AXILLA OVER 4CM	No
11406	EXCISION BENIGN CYST/MASS LESION CHEST OVER 4CM	No
11406	EXCISION BENIGN CYST/MASS LESION LOWER EXTREMITY OVER 4CM	No
11406	EXCISION BENIGN CYST/MASS LESION UPPER EXTREMITY OVER 4CM	No
11406	EXCISION BENIGN LESION > 4.0CM	No
11406	EXCISION BENIGN LESION BACK > 4.0CM	No
11406	EXCISION BENIGN LESION BUTTOCK > 4.0CM	No
11406	EXCISION BENIGN LESION INCLUDING MARGIN EXCEPT SKIN TAG AXILLA EXCISED DIAMETER OVER 4.0 CM	No
11406	EXCISION BENIGN LESION NOT SKIN TAG ABDOMEN OVER 4CM LESION	No
11406	EXCISION BENIGN LESION NOT SKIN TAG ARMS OVER 4CM LESION	No
11406	EXCISION BENIGN LESION NOT SKIN TAG LEGS OVER 4CM LESION	No
11406	EXCISION BENIGN LESION NOT SKIN TAG TRUNK OVER 4CM LESION	No
11406	EXCISION LIPOMA ABDOMEN	No
11406	EXCISION LIPOMA BACK	No
11406	EXCISION LIPOMA CHEST	No
11406	EXCISION LIPOMA EXTREMITY LOWER	No
11406	EXCISION LIPOMA EXTREMITY UPPER	No
11406	REVISION SCAR ABDOMINAL > 4.0CM	No

MSBOS - All records

Individual patient parameters (i.e., anemia, coagulopathy, transfusion risk) should be taken into consideration and may warrant an individual TSCR when not generally recommended.

June 22, 2023 **Sorted by CPT**

Primary CPT	Procedure Name	TSCR (Type and Screen) Needed?
11406	REVISION SCAR BACK > 4.0CM	No
11406	REVISION SCAR BREAST > 4.0CM	No
11422	EXCISE PLANTAR WARTS FROM FOOT/FEET 1.1 - 2.0CM LESION(S)	No
11422	EXCISION BENIGN CYST FROM FEMALE GENITALS 1.1-2.0CM MARGIN	No
11422	EXCISION BENIGN CYST FROM MALE GENITALS 1.1-2.0CM MARGIN	No
11422	EXCISION BENIGN LESION 1.1 TO 2.0 CM	No
11422	EXCISION BENIGN LESION GENITALS 1.1 TO 2.0 CM	No
11422	EXCISION BENIGN LESION HANDS 1.1 TO 2.0 CM	No
11422	EXCISION BENIGN LESION NECK 1.1 TO 2.0 CM	No
11422	EXCISION BENIGN LESION SCALP 1.1 TO 2.0 CM	No
11422	EXCISION KERATOSIS HAND(S) 1.1 2.0 CM LESION	No
11422	EXCISION SCAR FEMALE PERINEUM 1.1 - 2.0CM	No
11423	EXCISION BENIGN CYST FROM FEMALE GENITALS 2.1-3.0CM MARGIN	No
11423	EXCISION BENIGN CYST FROM MALE GENITALS 2.1-3.0CM MARGIN	No
11423	EXCISION BENIGN LESION GENITALS 2.1 TO 3.0 CM	No
11423	EXCISION BENIGN LESION HANDS 2.1 TO 3.0 CM	No
11423	EXCISION BENIGN LESION NECK 2.1 TO 3.0 CM	No
11423	EXCISION BENIGN LESION SCALP 2.1 TO 3.0 CM	No
11423	EXCISION CYST / MASS	No
11423	EXCISION CYST / MASS HEAD	No
11423	EXCISION CYST SEBACEOUS SCALP	No
11423	EXCISION LESION HEAD	No
11423	EXCISION SCALP	No
11426	EXCISE PLANTAR WARTS FROM FOOT/FEET >4 LESION(S)	No
11426	EXCISION BENIGN CYST FROM FEMALE GENITALS >4.0CM MARGIN	No
11426	EXCISION BENIGN CYST FROM MALE GENITALS >4.0CM MARGIN	No
11426	EXCISION BENIGN LESION FEET OVER 4.0 CM	No
11426	EXCISION BENIGN LESION GENITALS OVER 4.0 CM	No
11426	EXCISION BENIGN LESION HANDS OVER 4.0 CM	No
11426	EXCISION BENIGN LESION NECK OVER 4.0 CM	No
11426	EXCISION BENIGN LESION OVER 4.0 CM	No
11426	EXCISION BENIGN LESION SCALP OVER 4.0 CM	No
11426	EXCISION KERATOSIS NECK > 4.0 CM LESION	No
11426	EXCISION OF CONDYLOMA GENITALIA OVER 4.0 CM	No
11426	REVISION SCAR HEAD > 4.0CM	No
11426	REVISION SCAR NECK > 4.0CM	No
11442	EXCISION BENIGN LESION 1.1-2.0CM	No

MSBOS - All records

Individual patient parameters (i.e., anemia, coagulopathy, transfusion risk) should be taken into consideration and may warrant an individual TSCR when not generally recommended.

June 22, 2023 **Sorted by CPT**

Primary CPT	Procedure Name	TSCR (Type and Screen) Needed?
11442	EXCISION BENIGN LESION 1.1-2.0CM	No
11442	EXCISION BENIGN LESION FROM EARS 1.1 - 2.0 CM	No
11442	EXCISION BENIGN LESION FROM EYELIDS 1.1 - 2.0 CM	No
11442	EXCISION BENIGN LESION FROM FACE 1.1 - 2.0 CM	No
11442	EXCISION BENIGN LESION FROM LIPS 1.1 - 2.0 CM	No
11442	EXCISION BENIGN LESION FROM NOSE 1.1 - 2.0 CM	No
11442	EXCISION OTHER BENIGN LESION INCLUDING MARGINS EXCEPT SKIN TAG FACE EARS EYELIDS NOSE LIPS MUCOUS MEMBRANE EXCISED DIAMETER 1.1 TO 2.0 CM	No
11442	EXCISION SKIN CYST EAR 1.1 2.0 LESION	No
11442	EXCISION SKIN CYST FACE 1.1 2.0 LESION	No
11606	EXCISION MALIGNANT LESION ABDOMEN OVER 4.0CM	No
11606	EXCISION MALIGNANT LESION ARM OVER 4.0 CM	No
11606	EXCISION MALIGNANT LESION BACK OVER 4.0CM	No
11606	EXCISION MALIGNANT LESION LEGS OVER 4.0 CM	No
11606	EXCISION MALIGNANT LESION OVER 4.0 CM	No
11606	EXCISION MALIGNANT LESION TRUNK OVER 4.0 CM	No
11606	EXCISION MALIGNANT LESION TRUNK OVER 4.0CM	No
11606	EXCISION MALIGNANT MELANOMA LESION BACK OVER 4.0 CM	No
11606	EXCISION MELANOMA EXTREMITY LOWER	No
11606	EXCISION MELANOMA EXTREMITY UPPER	No
11770	EXCISION OF PILONIDAL SINUS TRACT, SIMPLE, SINGLE LAYERED SUTURE	No
11770	EXCISION PILONIDAL CYST SIMPLE	No
11770	EXCISION PILONIDAL CYST SIMPLE	No
11771	EXCISION CYST PILONIDAL EXTENSIVE	No
11771	EXCISION CYST PILONIDAL EXTENSIVE	No
11771	EXCISION OF PILONIDAL SINUS TRACT, EXTENSIVE TO FASCIA W/ SUBQ SUTURE	No
11970	GLUTEAL AUGMENTATION REPLACE TISSUE EXPANDER W/ PERM IMPLANT BILAT	No
11970	REPLACE TISSUE EXPANDER BACK	No
11970	REPLACE TISSUE EXPANDER UPPER EXTREMITY	No
11970	REPLACE TISSUE EXPLANDER	No
11970	REPLACE TISSUE EXPLANDER ABDOMEN	No
11970	REPLACEMENT TISSUE EXPANDER W/ PERMANENT IMPLANT	No
11971	REMOVAL EXPANDER TISSUE BREAST	No
11971	REMOVAL OF TISSUE EXPANDER(S) W/O INSERTION OF IMPLANT	No
11971	REMOVAL OF TISSUE EXPANDER(S) W/O INSERTION OF IMPLANT	No
12031	INTERMED. REPAIR WOUND OF ABDOMEN 2.5 CM OR LESS	No
12031	INTERMEDIATE REPAIR < 2.6CM	No

MSBOS - All records

Individual patient parameters (i.e., anemia, coagulopathy, transfusion risk) should be taken into consideration and may warrant an individual TSCR when not generally recommended.

June 22, 2023 **Sorted by CPT**

Primary CPT	Procedure Name	TSCR (Type and Screen) Needed?
12031	INTERMEDIATE REPAIR BACK < 2.6CM	No
12031	INTERMEDIATE REPAIR CHEST < 2.6CM	No
12031	INTERMEDIATE REPAIR LOWER EXTREMITY < 2.6CM	No
12031	INTERMEDIATE REPAIR SCALP < 2.6CM	No
12031	INTERMEDIATE REPAIR TRUNK < 2.6CM	No
12031	INTERMEDIATE REPAIR UPPER EXTREMITY < 2.6CM	No
12031	REPAIR INTERMEDIATE WOUND OF TRUNK 2.5 CM OR LESS	No
12032	INTERMED. REPAIR WOUND BACK 2.6 CM TO 7.5 CM	No
12032	INTERMED. REPAIR WOUND OF ABDOMEN 2.6 CM TO 7.5 CM	No
12032	INTERMEDIATE REPAIR 2.6-7.5CM	No
12032	INTERMEDIATE REPAIR AXILLAE 2.6-7.5CM	No
12032	INTERMEDIATE REPAIR BACK 2.6-7.5CM	No
12032	INTERMEDIATE REPAIR CHEST 2.6-7.5CM	No
12032	INTERMEDIATE REPAIR LOWER EXTREMITY 2.6-7.5CM	No
12032	INTERMEDIATE REPAIR SCALP 2.6-7.5CM	No
12032	INTERMEDIATE REPAIR TRUNK 2.6-7.5CM	No
12032	INTERMEDIATE REPAIR UPPER EXTREMITY 2.6-7.5CM	No
13121	CLOSURE WOUND EXTREMITY	No
13121	CLOSURE WOUND EXTREMITY LOWER	No
13121	CLOSURE WOUND EXTREMITY UPPER	No
13121	CLOSURE WOUND HEAD	No
13121	EXCISION LESION SCALP 2.6 CM-7.5 CM	No
13121	EXCISION SCAR LOWER EXTREMITY 2.6 CM-7.5 CM	No
13121	EXCISION SCAR UPPER EXTREMITY 2.6 CM-7.5 CM	No
13121	REPAIR LACERATION EXTREMITY LOWER	No
13121	REPAIR SCALP 2.6 CM TO 7.5 CM COMPLEX	No
13160	CLOSURE WOUND ABDOMEN	No
13160	DELAYED PRIMARY CLOSURE WOUND ANKLE	No
13160	REPAIR WOUND DEHISCENCE ABDOMEN COMPLEX	No
13160	REPAIR/CLOSURE OF SURGICAL WOUND	No
13160	REPAIR/CLOSURE OF SURGICAL WOUND	No
13160	SECONDARY CLOSURE OF DEHISCENCE UPPER EXTREMITY	No
13160	SECONDARY CLOSURE OF SURGICAL WOUND OR DEHISCENCE,EXTENSIVE OR COMPLICATED	No
13160	SECONDARY CLOSURE OF WOUND DEHISENCE TRUNK / GROIN	No
13160	SECONDARY CLOSURE WOUND ABDOMEN	No
13160	SECONDARY CLOSURE WOUND BACK	No
13160	SECONDARY CLOSURE WOUND CHEST	No

MSBOS - All records

Individual patient parameters (i.e., anemia, coagulopathy, transfusion risk) should be taken into consideration and may warrant an individual TSCR when not generally recommended.

June 22, 2023 **Sorted by CPT**

Primary CPT	Procedure Name	TSCR (Type and Screen) Needed?
13160	SECONDARY CLOSURE WOUND EXTREMITY LOWER	No
13160	SECONDARY CLOSURE WOUND EXTREMITY UPPER	No
13160	SECONDARY WOUND CLOSURE UPPER EXTREMITY COMPLEX	No
14041	ADJACENT TISSUE TRANSFER/REARRANGEMENT FEET DEFECT 10.1 SQ CM TO 30.0 SQ CM	No
14041	TRANSFER / REARRANGEMENT ADJACENT TISSUE 10.1 SQ CM TO 30.0 SQ CM	No
14041	TRANSFER / REARRANGEMENT ADJACENT TISSUE FACE/NECK DEFECT 10.1 SQ CM TO 30.0 SQ CM	No
14041	TRANSFER / REARRANGEMENT ADJACENT TISSUE GENITALIA DEFECT 10.1 SQ CM TO 30.0 SQ CM	No
14060	TRANSFER / REARRANGEMENT ADJACENT TISSUE EYELIDS 10SQCM OR LESS	No
14060	TRANSFER / REARRANGEMENT ADJACENT TISSUE EYELIDS 10SQCM OR LESS	No
14060	TRANSFER / REARRANGEMENT ADJACENT TISSUE NOSE </= 10SQCM	No
14060	TRANSFER / REARRANGEMENT,ADJACENT TISSUE LIPS DEFECT 10 SQ CM OR LESS	No
14060	TRANSFER / REARRANGEMENT,ADJACENT TISSUE, EYELIDS, NOSE, EARS DEFECT 10 SQ CM OR LESS	No
14301	ADJACENT TISSUE TRANSFER / REARRANGEMENT 30.1-60.0 SQCM	No
14301	ADJACENT TISSUE TRANSFER / REARRANGEMENT ABDOMEN 30.1-60.0 SQCM	No
14301	ADJACENT TISSUE TRANSFER / REARRANGEMENT BACK 30.1-60.0 SQCM	No
14301	ADJACENT TISSUE TRANSFER / REARRANGEMENT BUTTOCKS 30.1-60.0 SQCM	No
14301	ADJACENT TISSUE TRANSFER / REARRANGEMENT CHEST 30.1-60.0 SQCM	No
14301	ADJACENT TISSUE TRANSFER / REARRANGEMENT HEAD / NECK 30.1-60.0 SQCM	No
14301	ADJACENT TISSUE TRANSFER / REARRANGEMENT LOWER EXTREMITY 30.1-60.0 SQCM	No
14301	ADJACENT TISSUE TRANSFER / REARRANGEMENT PELVIS / HIP 30.1-60.0 SQCM	No
14301	ADJACENT TISSUE TRANSFER / REARRANGEMENT UPPER EXTREMITY 30.1-60.0 SQCM	No
14301	ADJACENT TISSUE TRANSFER OR REARRANGEMENT TRUNK DEFECT 30.1 SQ CM TO 60.0 SQ CM	No
15002	CREATION RECIPIENT SITE VIA EXCISION BACK 1ST 100SQCM ADULT	Yes
15002	CREATION RECIPIENT SITE VIA EXCISION BUTTOCKS 1ST 100 SQCM ADULT	Yes
15002	CREATION RECIPIENT SITE VIA EXCISION; 1ST 100SQCM ADULT	Yes
15002	CREATION RECIPIENT SITE VIA EXCISION; LOWER EXTREMITY 1ST 100SQ CM ADULT	Yes
15002	CREATION RECIPIENT SITE VIA EXCISION; TRUNK 1ST 100SQCM ADULT	Yes
15002	CREATION RECIPIENT SITE VIA EXCISION; UPPER EXTREMITY 1ST 100SQCM ADULT	Yes
15002	SURGICAL PREPERATION OF RECIPENT SITE, TRUNK, ARMS, LEGS, FIRST 100 SQ CM OR 1% BODY AREA PEDIATRIC	Yes
15004	CREATION RECIPIENT SITE VIA EXCISION	No
15004	CREATION RECIPIENT SITE VIA EXCISION; FACE/EARS/EYES 1ST 100SQCM ADULT	No
15004	CREATION RECIPIENT SITE VIA EXCISION; FEET 1ST 100SQCM ADULT	No
15004	CREATION RECIPIENT SITE VIA EXCISION; GENITAL 1ST 100SQCM ADULT	No
15004	CREATION RECIPIENT SITE VIA EXCISION; HANDS 1ST 100SQCM ADULT	No
15004	SURGICAL PREPERATION RECIPENT SITE, FACE, SCALP, EYELIDS, MOUTH, NECK, EARS, ORBITS, FIRST 100 SQ CM OR 1% BODY AREA, PEDIATRIC	No
15004	SURGICAL PREPERATION RECIPENT SITE, FEET AND/ OR MULTIPLE DIGITS FIRST 100 SQ CM OR 1% BODY AREA, PEDIATRIC	No

MSBOS - All records

Individual patient parameters (i.e., anemia, coagulopathy, transfusion risk) should be taken into consideration and may warrant an individual TSCR when not generally recommended.
June 22, 2023 **Sorted by CPT**

Primary CPT	Procedure Name	TSCR (Type and Screen) Needed?
15100	GRAFT SKIN SPLIT THICKNESS AUTOGRAFT	No
15100	GRAFT SKIN SPLIT THICKNESS AUTOGRAFT KNEE, FIRST 100 SQ CM OR LESS, OR 1% BODY AREA	No
15100	GRAFT SKIN SPLIT THICKNESS AUTOGRAFT LEGS, FIRST 100 SQ CM OR LESS, OR 1% BODY AREA	No
15100	GRAFT SKIN SPLIT THICKNESS AUTOGRAFT, TRUNK FIRST 100 SQ CM OR LESS, OR 1% BODY AREA	No
15100	GRAFT SKIN SPLIT THICKNESS AUTOGRAFT, TRUNK, ARMS, LEGS, FIRST 100 SQ CM OR LESS, OR 1% BODY AREA OF PEDIATRIC	No
15100	GRAFT SKIN SPLIT THICKNESS AUTOGRAFT,TRUNK,ARMS,LEGS,FIRST 100 SQ CM OR LESS	No
15100	SPLIT-THICKNESS AUTOGRAFT W/ CLOSURE DONOR SITE BACK 1ST 100SQCM	No
15100	SPLIT-THICKNESS AUTOGRAFT W/ CLOSURE DONOR SITE BUTTOCK 1ST 100SQCM	No
15100	SPLIT-THICKNESS AUTOGRAFT, THIGH; FIRST 100 SQ CM OR LESS	No
15734	BILATERAL MUSCLE FLAP CLOSURE SACRUM	Yes
15734	FLAP MUSCLE MYOCUTANEOUS OR FASCIOCUTANEOUS	Yes
15734	FLAP MUSCLE MYOCUTANEOUS OR FASCIOCUTANEOUS, TRUNK	Yes
15738	FLAP GASTROCNEMIUS	Yes
15738	FLAP MUSCLE MYOCUTANEOUS OR FASCIOCUTANEOUS	Yes
15738	FLAP MUSCLE MYOCUTANEOUS OR FASCIOCUTANEOUS, LOWER EXTREMITY	Yes
15758	FREE FASCIAL FLAP W/ MICROVASCULAR ANASTOMOSIS LOWER EXTREMITY	No
15771	GRAFTING OF AUTOLOGOUS FAT BY LIPO 50 CC OR LESS	No
15771	GRAFTING OF AUTOLOGOUS FAT BY LIPO 50 CC OR LESS	No
15771	GRAFTING OF AUTOLOGOUS FAT BY LIPO 50 CC OR LESS TRUNK, BREAST, SCALP, ARMS AND/OR LEGS	No
15823	BLEPHAROPLASTY	No
15823	BLEPHAROPLASTY LOWER LID VIA LASER BILATERAL	No
15823	BLEPHAROPLASTY UPPER LID EXCESSIVE SKIN WEIGHTING DOWN LID	No
15823	BLEPHAROPLASTY UPPER LID FOR EXCESSIVE SKIN WEIGHTING LID VIA LASER BILATERAL	No
15823	BLEPHAROPLASTY UPPER LID FOR EXCESSIVE SKIN WEIGHTING LID VIA LASER UNILATERAL [15917]	No
15823	BLEPHAROPLASTY UPPER MEDICALLY NECESSARY	No
15823	UPPER BLEPHAROPLASTY W/ MEDIAL FAT REMOVAL	No
15828	CHEEKLIFT	No
15828	FACELIFT	No
15828	FACELIFT CHEEK, CHIN, AND NECK	No
15830	PANNICULECTOMY	No
15830	PANNICULECTOMY	No
15830	REVISION PANNICULECTOMY	No
15847	ABDOMINOPLASTY UMBILICAL TRANSPOSITION AND FASCIAL PLICATION	No
15877	LIPO HARVEST OF FAT FROM ABDOMEN	No
15877	LIPOSUCTION	No
15877	LIPOSUCTION ABDOMEN	No
15877	LIPOSUCTION AXILLARY FOLDS	No

MSBOS - All records

Individual patient parameters (i.e., anemia, coagulopathy, transfusion risk) should be taken into consideration and may warrant an individual TSCR when not generally recommended.
June 22, 2023 **Sorted by CPT**

Primary CPT	Procedure Name	TSCR (Type and Screen) Needed?
15877	LIPOSUCTION FLANK BILATERAL	No
15877	LIPOSUCTION FLANK UNILATERAL	No
15877	LIPOSUCTION HIPS	No
15877	LIPOSUCTION TORSO	No
15879	LIPOSUCTION EXTREMITY LOWER	No
15879	LIPOSUCTION EXTREMITY LOWER	No
15879	ULTRASONIC LIPOSUCTION EXTREMITY LOWER	No
19120	EXCISION CYST BREAST MALE OR FEMALE OPEN	No
19120	EXCISION CYST BREAST MALE OR FEMALE OPEN	No
19120	EXCISION CYST BREAST MALE OR FEMALE OPEN BILATERAL	No
19120	EXCISION DUCT, BREAST; MALE OR FEMALE, OPEN	No
19120	OPEN EXCISION OF ABERRANT BREAST TISSUE BILATERAL FEMALE BREAST	No
19120	OPEN EXCISION OF ABERRANT BREAST TISSUE UNILATERAL FEMALE BREAST	No
19120	OPEN EXCISION OF BREAST CYST UNILATERAL FEMALE BREAST	No
19120	OPEN EXCISION OF BREAST LESION UNILATERAL FEMALE BREAST	No
19120	OPEN EXCISION OF BREAST LESION UNILATERAL MALE BREAST	No
19120	RE-EXCISION CYST/MASS BREAST	No
19125	EXCISION BREAST LESION IDENTIFIED BY PREOPERATIVE PLACEMENT RADIOLOGICAL MARKER, OPEN, SINGLE LESION	No
19301	LUMPECTOMY BREAST	No
19301	LUMPECTOMY BREAST	No
19301	MASTECTOMY PARTIAL	No
19303	MASTECTOMY SIMPLE	No
19303	MASTECTOMY SIMPLE	No
19303	MASTECTOMY SIMPLE BILATERAL	No
19307	MASTECTOMY MODIFIED	No
19307	MASTECTOMY MODIFIED	No
19307	MASTECTOMY MODIFIED BILATERAL	No
19307	MASTECTOMY MODIFIED RADICAL	No
19316	MASTOPEXY	No
19316	MASTOPEXY	No
19316	MASTOPEXY BILATERAL	No
19318	REDUCTION BREAST	No
19318	REDUCTION BREAST	No
19318	REDUCTION BREAST BILATERAL	No
19325	AUGMENTATION BREAST	No
19325	AUGMENTATION BREAST TRANSAXILLARY W/IMPLANT BILATERAL	No
19325	AUGMENTATION BREAST W/IMPLANT	No

MSBOS - All records

Individual patient parameters (i.e., anemia, coagulopathy, transfusion risk) should be taken into consideration and may warrant an individual TSCR when not generally recommended.

June 22, 2023 **Sorted by CPT**

Primary CPT	Procedure Name	TSCR (Type and Screen) Needed?
19325	AUGMENTATION BREAST W/IMPLANT BILATERAL	No
19328	REMOVAL IMPLANT BREAST	No
19328	REMOVAL IMPLANT BREAST	No
19328	REMOVAL IMPLANT BREAST BILATERAL	No
19342	INSERTION OR REPLACEMENT OF BREAST IMPLANT ON SEPARATE DAY FROM MASTECTOMY	No
19342	INSERTION OR REPLACEMENT OF BREAST IMPLANT ON SEPARATE DAY FROM MASTECTOMY BILATERAL	No
19342	INSERTION OR REPLACEMENT OF BREAST IMPLANT ON SEPARATE DAY FROM MASTECTOMY UNILATERAL	No
19357	INSERTION TISSUE EXPANDER PLACEMENT IN BREAST	No
19357	INSERTION TISSUE EXPANDER PLACEMENT IN BREAST	No
19357	TISSUE EXPANDER PLACEMENT IN BREAST RECONSTRUCTION	No
19357	TISSUE EXPANDER PLACEMENT IN BREAST RECONSTRUCTION BILATERAL	No
19357	TISSUE EXPANDER PLACEMENT IN BREAST RECONSTRUCTION STAGE I	No
19357	TISSUE EXPANDER PLACEMENT IN BREAST RECONSTRUCTION UNILATERAL	No
19364	RECONSTRUCTION BREAST FREE FLAP	No
19370	REVISION OF IMPLANT CAPSULE,BREAST,INCLD CAPSULOTOMY,CAPSULORRHAPHY,AND/OR PARTIAL CAPSULECTOMY	No
19371	CAPSULECTOMY BREAST IMPLANT COMPLETE INCLD REMOVAL OF ALL INTRACAPSULAR CONTENTS	No
19380	ADJACENT TISSUE TRANSFER/REARRANGEMENT BREAST	No
19380	REVISE RECONSTRUCTED BREAST	No
19380	REVISE RECONSTRUCTED BREAST UNILATERAL	No
19380	REVISION OF RECONSTRUCTED BREAST BILATERAL	No
20680	REMOVAL ARCH BARS MAXILLA MANDIBLE	No
20680	REMOVAL BURIED HARDWARE	No
20680	REMOVAL BURIED HARDWARE CHEST /STERNUM	No
20680	REMOVAL BURIED HARDWARE CHEST /STERNUM PEDIATRIC	No
20680	REMOVAL EXTERNAL FIXATOR EXTREMITY LOWER	No
20680	REMOVAL HARDWARE	No
20680	REMOVAL HARDWARE ANKLE	No
20680	REMOVAL HARDWARE ANKLE PEDIATRIC	No
20680	REMOVAL HARDWARE ARM	No
20680	REMOVAL HARDWARE ARM PEDIATRIC	No
20680	REMOVAL HARDWARE BACK	No
20680	REMOVAL HARDWARE CLAVICLE	No
20680	REMOVAL HARDWARE ELBOW	No
20680	REMOVAL HARDWARE ELBOW PEDIATRIC	No
20680	REMOVAL HARDWARE FEMUR	No
20680	REMOVAL HARDWARE FEMUR PEDIATRIC	No
20680	REMOVAL HARDWARE FOOT	No

MSBOS - All records

Individual patient parameters (i.e., anemia, coagulopathy, transfusion risk) should be taken into consideration and may warrant an individual TSCR when not generally recommended.

June 22, 2023 **Sorted by CPT**

Primary CPT	Procedure Name	TSCR (Type and Screen) Needed?
20680	REMOVAL HARDWARE FOOT PEDIATRIC	No
20680	REMOVAL HARDWARE HAND	No
20680	REMOVAL HARDWARE HAND PEDIATRIC	No
20680	REMOVAL HARDWARE HIP ADULT	No
20680	REMOVAL HARDWARE HIP PEDIATRIC	No
20680	REMOVAL HARDWARE HIP TOTAL	No
20680	REMOVAL HARDWARE HUMERUS	No
20680	REMOVAL HARDWARE HUMERUS PEDIATRIC	No
20680	REMOVAL HARDWARE KNEE TOTAL	No
20680	REMOVAL HARDWARE MANDIBLE	No
20680	REMOVAL HARDWARE MANDIBLE PEDIATRIC	No
20680	REMOVAL HARDWARE PELVIS	No
20680	REMOVAL HARDWARE RADIUS	No
20680	REMOVAL HARDWARE SHOULDER	No
20680	REMOVAL HARDWARE TIBIA	No
20680	REMOVAL HARDWARE TIBIA PEDIATRIC	No
20680	REMOVAL HARDWARE ULNA	No
20680	REMOVAL HARDWARE WRIST	No
20680	REMOVAL HARDWARE WRIST PEDIATRIC	No
20680	REMOVAL IMPLANT DEEP	No
20680	REMOVAL IMPLANT DEEP PEDIATRIC	No
20680	REMOVAL IMPLANT HIP	No
20680	REMOVAL IMPLANT KNEE (HARDWARE)	No
20680	REMOVAL IMPLANT PECTUS BAR	No
20680	REMOVAL NAIL / ROD INTRAMEDULLARY	No
20680	REMOVAL OF IMPLANT DEEP PATELLA	No
20680	REMOVAL OF IMPLANT MAXILLA	No
20680	REMOVE HARDWARE FROM SKULL	No
20690	APPLICATION EXTERNAL FIXATOR	No
20690	APPLICATION EXTERNAL FIXATOR ANKLE	No
20690	APPLICATION EXTERNAL FIXATOR FOOT	No
20690	APPLICATION EXTERNAL FIXATOR HUMERUS	No
20690	APPLICATION EXTERNAL FIXATOR TIBIA	No
20690	APPLICATION EXTERNAL FIXATOR WRIST PEDIATRIC	No
20690	APPLICATION OF UNIPLANE EXTERNAL FIXATION UNILATERAL FINGER	No
20690	APPLICATION UNIPLANE EXTERNAL FIXATOR UNILATERAL FEMUR	No
20692	APPLICATION MULTIPLANE EXTERNAL FIXATION SYSTEM UNILATERAL	No

MSBOS - All records

Individual patient parameters (i.e., anemia, coagulopathy, transfusion risk) should be taken into consideration and may warrant an individual TSCR when not generally recommended.
June 22, 2023 **Sorted by CPT**

Primary CPT	Procedure Name	TSCR (Type and Screen) Needed?
20692	APPLICATION MULTIPLANE EXTERNAL FIXATION SYSTEM UNILATERAL	No
20692	APPLICATION MULTIPLANE EXTERNAL FIXATION SYSTEM UNILATERAL PELVIS	No
20694	REMOVAL DISTRACTOR MANDIBULAR	No
20694	REMOVAL EXTERNAL FIXATION SYSTEM UNDER ANESTHESIA	No
20694	REMOVAL EXTERNAL FIXATION SYSTEM UNDER ANESTHESIA	No
21320	CLOSED REDUCTION NASAL	No
21320	CLOSED REDUCTION NASAL	No
21320	CLSD TX OF NASAL BONE FX W/MUA W/STBLN	No
21501	INCISION AND DRAINAGE ABSCESS	Yes
21501	INCISION AND DRAINAGE ABSCESS CHEST	Yes
21501	INCISION AND DRAINAGE ABSCESS NECK	Yes
21501	INCISION AND DRAINAGE HEMATOMA SOFT TISSUE OF NECK	Yes
21501	INCISION AND DRAINAGE HEMATOMA SOFT TISSUE OF THORAX	Yes
21552	EXCISION SOFT TISSUE TUMOR SUB-Q = / > 3CM	No
21552	EXCISION SOFT TISSUE TUMOR SUB-Q OF ANTERIOR THORAX = / > 3CM	No
21552	EXCISION SOFT TISSUE TUMOR SUB-Q OF NECK = / > 3CM	No
21555	EXCISION CYST / MASS NECK SUBCUTANEOUS < 3CM	No
21555	EXCISION CYST / MASS NECK SUBCUTANEOUS < 3CM	No
21555	EXCISION TUMOR THORAX SUBCUTANEOUS < 3CM	No
21555	EXCISION, TUMOR, SOFT TISSUE OF NECK OR ANTERIOR THORAX, SUBCUTANEOUS; LESS THAN 3 CM	No
21615	EXCISION RIB FIRST AND/OR CERVICAL	No
21627	DEBRIDEMENT STERNUM	Yes
21750	CLOSURE MEDIAN STERNOTOMY SEPARATION W OR W/O DEBRIDEMENT	Yes
21750	MEDIASTERNOTOMY	Yes
21750	MEDIASTERNOTOMY	Yes
21750	REPAIR OR REWIRE STERNAL DEHISCENCE	Yes
21931	EXCISION SOFT TISSUE TUMOR SUB-Q = / >3CM	No
21931	EXCISION SOFT TISSUE TUMOR SUB-Q OF BACK = / >3CM	No
21931	EXCISION SOFT TISSUE TUMOR SUB-Q OF FLANK = / > 3CM	No
22551	ARTHRODESIS ANT DISC PREP DISCECTOMY OSTEOPHYTECTOMY DECOMPRES S CORD/NERVE ROOT C' BELOW C2	No
22551	ARTHRODESIS ANT DISC PREP DISCECTOMY OSTEOPHYTECTOMY DECOMPRES S CORD/NERVE ROOT C' BELOW C2	No
22551	ARTHRODESIS ANTERIOR DISC PREP DISCECTOMY OSTEOPHYTECTOMY & DECOMPRESS NERVE ROOTS C' BELOW C2	No
22551	ARTHRODESIS ANTERIOR DISC PREP DISCECTOMY OSTEOPHYTECTOMY & DECOMPRESS SPINAL CORD C' BELOW C2	No
22558	ALIF DECOMPRESSION LAMINECTOMY INTERBODY FUSION LUMBAR LEVEL 1	No
22558	ARTHRODESIS ANTERIOR W/ DISCECTOMYLUMBAR 1 INTERSPACE	No
22558	DECOMPRESSION LAMINECTOMY INTERBODY FUSION LUMBAR LEVEL 1	No
22558	DISCECTOMY LUMBAR ANTERIOR WITH INTERBODY FUSION LEVEL 1	No

MSBOS - All records

Individual patient parameters (i.e., anemia, coagulopathy, transfusion risk) should be taken into consideration and may warrant an individual TSCR when not generally recommended.
June 22, 2023 **Sorted by CPT**

Primary CPT	Procedure Name	TSCR (Type and Screen) Needed?
22558	FUSION LUMBAR INTERBODY ANTERIOR LEVEL 1	No
22558	LATERAL INTERBODY FUSION (LIF); LUMBAR	No
22600	ARTHRODESIS CERVICAL POSTEROLATERAL BELOW C2 1ST SINGLE LEVEL	Yes
22600	ARTHRODESIS CERVICAL POSTERIOR, BELOW C2 1ST SINGLE LEVEL	Yes
22600	FUSION CERVICAL, BELOW C2, SINGLE LEVEL	Yes
22600	FUSION CERVICAL, BELOW C2, SINGLE LEVEL	Yes
22610	ARTHRODESIS THORACIC POSTERIOR TECHNIQUE 1ST LEVEL	Yes
22610	ARTHRODESIS THORACIC POSTEROLATERAL TECHNIQUE 1ST LEVEL	Yes
22610	FUSION THORACIC LEVEL 1	Yes
22610	FUSION THORACIC LEVEL 1	Yes
22610	FUSION THORACIC POSTERIOR LEVEL 1	Yes
22612	FUSION LUMBAR POSTERIOR LEVEL 1	No
22612	FUSION LUMBAR POSTERIOR LEVEL 1	No
22612	FUSION LUMBAR POSTERIOR LEVEL 1 WITH C ARM	No
22612	FUSION LUMBAR POSTERIOR WITH GRAFT BONE LEVEL 1	No
22612	NON-INSTRUMENTED FUSION LUMBAR POSTERIOR LEVEL 1	No
22630	DECOMPRESSION LAMINECTOMY INTERBODY FUSION LUMBAR POSTERIOR (PLIF) LEVEL 1	No
22630	DECOMPRESSION LAMINECTOMY INTERBODY FUSION LUMBAR POSTERIOR (PLIF) LEVEL 1	No
22630	FUSION LUMBAR	No
22630	FUSION LUMBAR INTERBODY POSTERIOR LEVEL 1	No
22630	MINIMALLY INVASIVE DECOMPRESSION LAMINECTOMY INTERBODY FUSION LUMBAR POSTERIOR (PLIF) LEVEL 1	No
22630	TLIF DECOMPRESSION LAMINECTOMY INTERBODY FUSION LUMBAR POSTERIOR (PLIF) LEVEL 1	No
22633	ARTHDSIS POST/POSTEROLATERAL TECH W/POST INTERBODY TECH LAM AND/OR DISCECT SUFF TO PREP INTERSPACE SINGLE INTERSPACE & SEG LUMBAR	No
22840	MINIMALLY INVASIVE POSTERIOR NONSEGMENTAL INSTRUMENTATION	Yes
22840	POSTERIOR NON-SEGMENTAL INSTRUMENTATION FOLLOWING FUSION 1 LEVEL PDFI	Yes
22840	POSTERIOR NON-SEGMENTAL INSTRUMENTATION FOLLOWING LUMBAR FUSION 1 LEVEL PDFI	Yes
22840	POSTERIOR NON-SEGMENTATAL INSTRUMENTATION FOLLOWING CERVICAL FUSION 1 LEVEL PDFI	Yes
22842	INSERT SPINE FIXATION DEVICE	Yes
22842	POSTERIOR SEGMENTAL INSTRUMENTATION FOLLOWING CERVICAL FUSION 3-6 LEVELS	Yes
22842	POSTERIOR SEGMENTAL INSTRUMENTATION FOLLOWING LUMBAR FUSION 3-6 LEVELS	Yes
22842	POSTERIOR SEGMENTAL INSTRUMENTATION FOLLOWING THORACIC FUSION 3-6 LEVELS	Yes
22842	POSTERIOR SEGMENTAL INSTRUMENTATION FOLLOWING THORACIC FUSION 3-6 LEVELS	Yes
22843	MIS INSERT SPINE FIXATION DEVICE 1 LEVEL	Yes
22843	POSTERIOR SEGMENTAL INSTRUMENTATION 7 TO 12 VERTEBRAL SEGMENTS	Yes
22843	POSTERIOR SEGMENTAL INSTRUMENTATION 7 TO 12 VERTEBRAL SEGMENTS	Yes
22843	POSTERIOR SEGMENTAL INSTRUMENTATION FOLLOWING CERVICAL FUSION 7-12 LEVELS	Yes

MSBOS - All records

Individual patient parameters (i.e., anemia, coagulopathy, transfusion risk) should be taken into consideration and may warrant an individual TSCR when not generally recommended.

June 22, 2023 **Sorted by CPT**

Primary CPT	Procedure Name	TSCR (Type and Screen) Needed?
22843	POSTERIOR SEGMENTAL INSTRUMENTATION FOLLOWING LUMBAR FUSION 7-12 LEVELS	Yes
22849	REINSERT SPINAL FIX DEVICE	Yes
22849	REPLACEMENT PEDICLE SCREW FIXATION DEVICE	Yes
22849	REVISION PEDICLE SCREW FIXATION DEVICE	Yes
22849	REVISION PEDICLE SCREW FIXATION DEVICE	Yes
22852	REMOVAL POSTERIOR SEGMENTAL INSTRUMENTATION	No
22853	INSERTION INTERBODY BIOMED DEVICE(S) W/ANT INSTR ANCHORING TO DISC SPACE W/INTERBODY FUSION,EA INTERSPACE	Yes
22856	TOTAL CERV DISK REPLACEMENT	No
22903	EXCISION SOFT TISSUE TUMOR SUB-Q OF ABDOMEN = / > 3CM	No
23412	OPEN REPAIR SHOULDER ROTATOR CUFF OPEN; CHRONIC	No
23472	ARTHROPLASTY REPLACE JOINT TOTAL SHOULDER	No
23472	ARTHROPLASTY SHOULDER	No
23472	ARTHROPLASTY SHOULDER	No
23472	ARTHROPLASTY TOTAL SHOULDER; W/ GLENOID & PROXIMAL HUMERAL REPLACEMENT	No
23472	REVERSE TOTAL SHOULDER ARTHROPLASTY	No
23472	REVISION JOINT TOTAL SHOULDER	No
23474	REVISE TOTAL SHOULDER ARTHROPLASTY	Yes if Hgb less than 9
23515	ORIF CLAVICLE	No
23515	ORIF CLAVICLE	No
23515	ORIF SHOULDER CLAVICULAR FRACTURE,	No
23615	OPEN REDUCTION PROXIMAL HUMERAL FRACTURE W/ INTERNAL FIXATION INCL.REPAIR TUBEROSITY(S)	No
23615	ORIF HUMERUS	No
23615	ORIF PROXIMAL HUMERUS	No
24071	EXCISION SOFT TISSUE TUMOR SUB-Q = / > 3CM	No
24071	EXCISION SOFT TISSUE TUMOR SUB-Q OF ELBOW = / > 3CM	No
24071	EXCISION SOFT TISSUE TUMOR SUB-Q OF UPPER ARM = / > 3CM	No
24342	REINSERT BICEP TENDON	No
24342	REINSERTION TRICEPS TENDON, DISTAL,	No
24342	REPAIR RUPTURED TENDON	No
24342	REPAIR RUPTURED TENDON BICEPS	No
24342	REPAIR RUPTURED TENDON TRICEPS	No
24515	ORIF HUMERUS FRACTURE WITH OR WITHOUT CERCLAGE	Yes
24515	ORIF HUMERUS FRACTURE WITH OR WITHOUT CERCLAGE	Yes
24515	ORIF HUMERUS FRACTURE WITH OR WITHOUT CERCLAGE WITH C-ARM	Yes
24538	PERCUTANEOUS SKELETAL FIX SUPRACONDYLAR HUMERAL FX	No
24538	PERCUTANEOUS SKELETAL FIX SUPRACONDYLAR HUMERAL FX W/INTERCONDYLAR EXTENSION	No
24538	PERCUTANEOUS SKELETAL FIX SUPRACONDYLAR HUMERAL FX W/INTERCONDYLAR EXTENSION PEDIATRIC	No

MSBOS - All records

Individual patient parameters (i.e., anemia, coagulopathy, transfusion risk) should be taken into consideration and may warrant an individual TSCR when not generally recommended.
June 22, 2023 **Sorted by CPT**

Primary CPT	Procedure Name	TSCR (Type and Screen) Needed?
24538	PERCUTANEOUS SKELETAL FIX SUPRACONDYLAR HUMERAL FX W/O INTERCONDYLAR EXTENSION	No
24538	PERCUTANEOUS SKELETAL FIX SUPRACONDYLAR HUMERAL FX W/O INTERCONDYLAR EXTENSION PEDIATRIC	No
24685	OPEN REDUCTION ULNAR FRACTURE	No
25000	INCISION EXTENSOR TENDON SHEATH WRIST	No
25000	INCISION EXTENSOR TENDON SHEATH WRIST	No
25000	RELEASE CONTRACTURE DEQUERVAINS	No
25111	EXCISION GANGLION WRIST	No
25111	EXCISION GANGLION WRIST	No
25111	EXCISION GANGLION WRIST PEDIATRIC	No
25447	ARTHROPLASTY CARPOMETACARPAL JOINTS	No
25447	ARTHROPLASTY WRIST	No
25447	ARTHROPLASTY WRIST INTERPOSITION INTERCARPAL OR CARPOMETACARPAL	No
25575	ORIF RADIUS AND ULNA	No
25606	FIXATION EPIPHYSEAL SEPARATION EXTERNAL SKELTAL	No
25606	FIXATION WRIST FRACTURE	No
25606	FIXATION WRIST FRACTURE EXTERNAL SKELTAL	No
25606	PERC SKELETAL FIXATION OF DISTAL RADIAL FRACTURE PEDIATRIC	No
25606	PERCUTANEOUS FIXATION DISTAL RADIAL FRACTURE OR EPIPHYSEAL SEPERATION	No
25606	PERCUTANEOUS PINNING WRIST WITH C-ARM	No
25607	ORIF DISTAL RADIAL EXTRA-ARTICULAR FRACTURE PEDIATRIC	No
25607	ORIF RADIUS DISTAL	No
25607	ORIF RADIUS DISTAL	No
25609	ORIF DISTAL RADIUS THREE OR MORE FRAGMENTS	No
26055	RELEASE TRIGGER FINGER	No
26055	RELEASE TRIGGER FINGER	No
26055	RELEASE TRIGGER THUMB	No
26160	EXCISION GANGLION	No
26160	EXCISION GANGLION FINGER	No
26160	EXCISION GANGLION HAND	No
26160	EXCISION LESION TENDON FINGER	No
26160	EXCISION LESION TENDON SHEATH HAND	No
26615	ORIF HAND	No
26615	ORIF HAND	No
26615	ORIF METACARPAL	No
26727	PERCUTANEOUS SKELETAL FIXATION UNSTABLE PHALANGEAL SHAFT FRACTURE, PROXIMAL OR MIDDLE PHALANX, FINGER OR THUMB, W/ MANIPULATION, EACH	No
26735	ORIF MIDDLE PHALANX	No

MSBOS - All records

Individual patient parameters (i.e., anemia, coagulopathy, transfusion risk) should be taken into consideration and may warrant an individual TSCR when not generally recommended.

June 22, 2023 **Sorted by CPT**

Primary CPT	Procedure Name	TSCR (Type and Screen) Needed?
26735	ORIF PHALANX	No
26735	ORIF PROXIMAL PHALANX	No
26735	ORIF THUMB WITH C-ARM	No
26951	AMPUTATION FINGER W/ NEURECTOMY	No
26951	AMPUTATION FINGER(S)	No
26951	AMPUTATION FINGER(S)	No
26951	AMPUTATION THUMB W/ NEURECTOMY	No
26990	INCISION AND DRAINAGE ABCESS HIP	Yes
26990	INCISION AND DRAINAGE DEEP PELVIS / HIP JOINT AREA ABSCESS	Yes
26990	INCISION AND DRAINAGE DEEP PELVIS / HIP JOINT AREA ABSCESS	Yes
26990	INCISION AND DRAINAGE HEMATOMA HIP	Yes
26990	INCISION AND DRAINAGE HEMATOMA PELVIC AREA	Yes
27030	ARTHROTOMY W/DRAINAGE HIP PEDIATRIC	Yes
27030	ARTHROTOMY HIP W/ DRAINAGE	Yes
27030	ARTHROTOMY HIP W/ DRAINAGE	Yes
27091	REMOVAL HIP PROSTHESIS COMPLICATED INCL. TOTAL HIP PROSTHESIS	Yes
27125	HEMIARTHROPLASTY HIP	Yes
27125	HEMIARTHROPLASTY REPLACE JOINT PARTIAL HIP	Yes
27125	REPAIR HEMIARTHROPLASTY HIP BIPOLAR	Yes
27125	REPAIR HEMIARTHROPLASTY HIP UNIPOLAR	Yes
27130	ARTHROPLASTY ACETABULAR AND PROX FEM PROSTH TOTAL HIP ANTERIOR APPROACH W/W/O AUTOGRAFT	No
27130	ARTHROPLASTY ACETABULAR AND PROX FEM PROSTH TOTAL HIP W/O AUTOGRAFT	No
27130	ARTHROPLASTY ACETABULAR AND PROX FEM PROSTHETIC TOTAL HIP W/ AUTOGRAFT	No
27130	ARTHROPLASTY HIP	No
27130	ARTHROPLASTY HIP	No
27130	ARTHROPLASTY REPLACE JOINT TOTAL HIP	No
27130	ARTHROPLASTY REPLACE JOINT TOTAL HIP BILATERAL	No
27130	ROBOTIC ASSISTED ANTERIOR TOTAL HIP ARTHROPLASTY	No
27130	ROBOTIC ASSISTED TOTAL HIP ARTHROPLASTY	No
27130	TOTAL HIP ARTHROPLASTY ACETABULAR & PROX FEM PROSTHETIC W/ AUTOGRAFT UNILAT	No
27130	TOTAL HIP ARTHROPLASTY ACETABULAR & PROX FEM PROSTHETIC W/O AUTOGRFT UNILAT	No
27132	ARTHROPLASTY TOTAL HIP CONVERSION AFTER PREVIOUS HIP SURG	Yes
27132	ARTHROPLASTY TOTAL HIP CONVERSION AFTER PREVIOUS HIP SURG	Yes
27132	ARTHROTOMY WITH REMOVAL AND/OR EXCHANGE OF HIP SPACER COMPLICATED	Yes
27134	REVISION JOINT TOTAL HIP	Yes
27134	REVISION JOINT TOTAL HIP BOTH COMPONENTS	Yes
27134	REVISION JOINT TOTAL HIP BOTH COMPONENTS UNILATERAL HIP	Yes

MSBOS - All records

Individual patient parameters (i.e., anemia, coagulopathy, transfusion risk) should be taken into consideration and may warrant an individual TSCR when not generally recommended.
June 22, 2023 **Sorted by CPT**

Primary CPT	Procedure Name	TSCR (Type and Screen) Needed?
27137	REVISION JOINT TOTAL HIP ACETABULAR COMPONENT ONLY	Yes
27138	REVISION JOINT TOTAL HIP FEMORAL COMPONENT ONLY	Yes
27235	PERCUTANEOUS FIXATION FEMORAL FRACTURE, PROXIMAL END, NECK	No
27236	IMPLANT SCREW CANNULATED HIP WITH C-ARM	No
27236	OPEN TREATMENT OF FEMORAL FRACTURE PROXIMAL END NECK INTERNAL FIXATION OR PROSTHETIC REPLACEMENT	No
27236	ORIF FEMUR	No
27236	ORIF FEMUR NECK	No
27236	ORIF HIP PROXIMAL END, NECK	No
27245	INSERTION NAIL / ROD INTERMEDULLARY FEMUR WITH C-ARM	No
27245	INSERTION NAIL HIP TK2 WITH C-ARM	No
27245	OPEN REDUCTION FEMUR W/ INTRAMEDULLARY IMPLANT	No
27245	OPEN TX INTERTROCHANTERIC / SUBTROCHANTERIC FX HIP/FEMUR INTRAMEDULARY W/ INTERLOCKING SCREWS OR CERCLAGE	No
27245	OPEN TX INTERTROCHANTERIC / SUBTROCHANTERIC FX HIP/FEMUR W/INTRAMEDULLARY ROD ONLY	No
27245	OPEN TX OF INTERTROCHANTERIC FX	No
27245	OPEN TX OF INTERTROCHANTERIC FX W/ INTRAMEDULLARY IMPLANT W/ SCREWS	No
27245	ORIF HIP WITH OR WITHOUT INTERLOCKING SCREWS/CERCLAGE	No
27266	CLOSED REDUCTION HIP DISLOCATION	No
27266	CLOSED REDUCTION HIP DISLOCATION POST ARTHROPLASTY	No
27266	CLOSED REDUCTION OF PERIPROSTHETIC HIP DISLOCATION	No
27299	DECOMPRESSION CORE HIP	Yes
27299	OSTEOARTICULAR TRANSFER PROCEDURE HIP	Yes
27299	RECONSTRUCTION OF HIP ABDUCTOR W/ALLOGRAFT AND TRANSFER OF GLUTEUS MAXIMUS TENDON	Yes
27299	REVISION OF TOTAL HIP ARTHROPLASTY ACETABULAR COMPONENT ONLY POLYETHYLENE EXCHANGE	Yes
27299	REVISION OF TOTAL HIP ARTHROPLASTY ACETABULAR COMPONENT ONLY POLYETHYLENE EXCHANGE	Yes
27299	SUTURE ABDUCTOR TENDON HIP	Yes
27299	SUTURE REPAIR GLUTEUS MEDIUS	Yes
27301	INCISION AND DRAINAGE ABSCESS , THIGH OR KNEE REGION	Yes
27301	INCISION AND DRAINAGE BURSA KNEE	Yes
27301	INCISION AND DRAINAGE DEEP ABSCESS KNEE REGION	Yes
27301	INCISION AND DRAINAGE DEEP ABSCESS THIGH	Yes
27301	INCISION AND DRAINAGE DEEP HEMATOMA	Yes
27301	INCISION AND DRAINAGE DEEP HEMATOMA KNEE REGION	Yes
27301	INCISION AND DRAINAGE DEEP HEMATOMA THIGH	Yes
27310	ARTHROTOMY KNEE W/ EXPLORATION	Yes
27310	ARTHROTOMY KNEE W/ EXPLORATION	Yes
27310	ARTHROTOMY KNEE W/ EXPLORATION AND DRAINAGE	Yes

MSBOS - All records

Individual patient parameters (i.e., anemia, coagulopathy, transfusion risk) should be taken into consideration and may warrant an individual TSCR when not generally recommended.

June 22, 2023 **Sorted by CPT**

Primary CPT	Procedure Name	TSCR (Type and Screen) Needed?
27310	ARTHROTOMY KNEE W/ EXPLORATION AND REMOVAL OF FOREIGN BODY	Yes
27310	ARTHROTOMY KNEE W/ EXPLORATION AND REMOVAL OF FOREIGN BODY PEDIATRIC	Yes
27337	EXCISION SOFT TISSUE TUMOR SUB-Q = / > 3CM	No
27337	EXCISION SOFT TISSUE TUMOR SUB-Q OF KNEE AREA = / > 3CM	No
27337	EXCISION SOFT TISSUE TUMOR SUB-Q OF THIGH = / > 3CM	No
27385	SUTURE OF HAMSTRING MUSCLE RUPTURE PRIMARY	No
27385	SUTURE OF MUSCLE RUPTURE PRIMARY	No
27385	SUTURE OF QUADRICEPS MUSCLE RUPTURE PRIMARY	No
27446	ARTHROPLASTY KNEE CONDYLE & PLATEAU LATERAL COMPARTMENT	No
27446	ARTHROPLASTY KNEE CONDYLE & PLATEAU MEDIAL OR LATERAL	No
27446	ARTHROPLASTY REPLACE JOINT TOTAL KNEE UNI	No
27446	ARTHROPLASTY REPLACE JOINT TOTAL KNEE UNI	No
27446	ROBOTIC ASSISTED UNI KNEE ARTHROPLASTY	No
27447	ARTHROPLASTY KNEE CONDYLE AND PLATEAU MEDIAL AND LATERAL COMPARTMENTS W/O PATELLA RESURFACING	No
27447	ARTHROPLASTY REPLACE JOINT TOTAL KNEE	No
27447	ARTHROPLASTY REPLACE JOINT TOTAL KNEE	No
27447	ARTHROPLASTY REPLACE JOINT TOTAL KNEE BILATERAL	No
27447	ROBOTIC ASSISTED TOTAL KNEE ARTHROPLASTY	No
27447	ROBOTIC ASSISTED TOTAL KNEE ARTHROPLASTY BILATERAL	No
27486	REVISION JOINT TOTAL KNEE ONE COMPONENT	No
27487	REVISION JOINT TOTAL KNEE	No
27487	REVISION JOINT TOTAL KNEE FEMORAL AND ENTIRE TIBIAL COMPONENT	No
27487	REVISION JOINT TOTAL KNEE INFECTION	No
27488	REMOVAL PROSTHESIS JOINT KNEE	Yes
27506	INSERTION NAIL / ROD	Yes
27506	INSERTION NAIL / ROD INTRAMEDULLARY OPEN REDUCTION FEMUR	Yes
27506	INSERTION NAIL/ ROD FEMORAL ANTEGRADE	Yes
27506	INSERTION NAIL/ROD FEMORAL RETROGRADE	Yes
27507	ORIF FEMUR WITH PLATING	Yes
27511	OPEN TX FEMORAL SUPRACONDYLAR FX WO/ INTERCONDYLAR EXTENSION	Yes
27511	OPEN TX FEMORAL SUPRACONDYLAR FX WO/ INTERCONDYLAR EXTENSION W/INT FIXATION	Yes
27511	OPEN TX FEMORAL SUPRACONDYLAR FX WO/ INTERCONDYLAR EXTENSION WO/INT FIXATION	Yes
27511	ORIF SUPRACONDYLAR OR TRANSCONDYLAR DISTAL FEMUR FRACTURE WITHOUT INTERCONDYLAR EXTENSION	Yes
27513	ORIF FEMUR DISTAL (SUPRACONDYLAR)	Yes
27513	ORIF FEMUR DISTAL (SUPRACONDYLAR)	Yes
27513	ORIF INTERCONDYLAR DISTAL FEMUR FRACTURE	Yes
27524	ORIF OF PATELLAR FRACTURE AND/OR PARTIAL OR COMPLETE PATELLECTOMY AND SOFT TISSUE REPAIR	No

MSBOS - All records

Individual patient parameters (i.e., anemia, coagulopathy, transfusion risk) should be taken into consideration and may warrant an individual TSCR when not generally recommended.

June 22, 2023 **Sorted by CPT**

Primary CPT	Procedure Name	TSCR (Type and Screen) Needed?
27524	ORIF PATELLA	No
27524	ORIF PATELLA	No
27524	ORIF PATELLA FRACTURE	No
27535	ORIF TIBIAL PLATEAU	No
27536	OPEN REDUCTION TIBIAL FRACTURE, PROXIMAL (PLATEAU) BICONDYLAR	No
27570	EXAM UNDER ANESTHESIA, KNEE	No
27570	MANIPULATION KNEE JOINT UNDER GENERAL ANES	No
27570	MANIPULATION KNEE JOINT UNDER GENERAL ANES	No
27590	AMPUTATION ABOVE KNEE EXTREMITY LOWER	Yes
27590	AMPUTATION ABOVE KNEE EXTREMITY LOWER	Yes
27590	AMPUTATION REVISION CONVERSION BKA TO ABOVE THE KNEE AMPUTATION	Yes
27603	INCISION AND DRAINAGE ABSCESS LEG OR ANKLE	No
27603	INCISION AND DRAINAGE ABSCESS LEG OR ANKLE	No
27603	INCISION AND DRAINAGE DEEP ABSCESS ANKLE	No
27603	INCISION AND DRAINAGE DEEP ABSCESS LEG	No
27650	REPAIR TENDON ACHILLES	No
27759	INSERTION NAIL / ROD INTRAMEDULLARY TIBIA	No
27759	INSERTION NAIL / ROD INTRAMEDULLARY TIBIA	No
27759	INSERTION NAIL / ROD INTRAMEDULLARY TIBIA PEDIATRIC	No
27759	RODDING TIBIAL WITH C-ARM	No
27792	OPEN REDUCTION DISTAL FIBULAR FX. W/ INTERNAL FIXATION WHEN PERFORMED	No
27814	ORIF ANKLE BIMALLEOLAR	No
27822	ORIF ANKLE TRIMALLEOLAR, WITHOUT FIXATION POSTERIOR LIP	No
27823	ORIF ANKLE TRIMALLEOLAR, WITH FIXATION POSTERIOR LIP	No
27827	OPEN REDUCTION ANKLE DISTAL TIBIA W/ INTERNAL FIXATION	No
27827	OPEN REDUCTION ANKLE DISTAL TIBIA W/ INTERNAL FIXATION	No
27827	ORIF PILON ANKLE FRACTURE OF WEIGHT BEARING ARTICULAR SURFACE/PORTION OF DISTAL TIBIA, TIBIA ONLY	No
27828	ORIF DISTAL TIBIA W/ TIB/FIB FX BILATERAL	No
27828	ORIF PILON ANKLE FRACTURE OF WEIGHT BEARING ARTICULAR SURFACE/PORTION OF DISTAL TIBIA, TIBIA AND FIBULA	No
27828	ORIF TIBIA AND FIBULA	No
27828	ORIF TIBIA AND FIBULA	No
27829	OPEN REDUCTION JOINT DISTAL TIBIOFIBULAR	No
27829	ORIF ANKLE SYNDESMOSIS	No
27829	ORIF SYNDESMOSIS LOWER EXTREMITY	No
27829	ORIF SYNDESMOSIS LOWER EXTREMITY	No
27870	ARTHRODESIS ANKLE	No
27870	ARTHRODESIS ANKLE	No

MSBOS - All records

Individual patient parameters (i.e., anemia, coagulopathy, transfusion risk) should be taken into consideration and may warrant an individual TSCR when not generally recommended.

June 22, 2023 **Sorted by CPT**

Primary CPT	Procedure Name	TSCR (Type and Screen) Needed?
27870	FUSION ANKLE	No
27880	AMPUTATION BELOW KNEE EXTREMITY LOWER	Yes
27882	AMPUTATION LEG THROUGH TIBIA AND FIBULA OPEN CIRCULAR	Yes
27882	AMPUTATION LEG THROUGH TIBIA AND FIBULA OPEN CIRCULAR	Yes
27882	AMPUTATION REVISION, AMPUTATE LEG THRU TIBIA & FIBULA OPEN CIRCULAR	Yes
27886	AMPUTATION REVISION BELOW KNEE	Yes
28001	INCISION AND DRAINAGE FOOT, BURSA	No
28002	INCISION AND DRAINAGE FOOT, BELOW FASCIA, BURSAL SPACE, SINGLE	No
28003	INCISION AND DRAINAGE FOOT, BELOW FASCIA, MULTIPLE AREAS	No
28005	INCISION BONE CORTEX FOOT	No
28120	EXCISION BONE CALCANEAL TUBEROSITY	No
28120	EXCISION BONE CALCANEAL TUBEROSITY	No
28120	EXCISION BONE TALUS CALCANEUS	No
28122	EXCISION BONE METATARSAL	No
28285	CORRECTION HAMMER TOE ONE TOE UNILATERAL	No
28285	RECONSTRUCTION TOE HAMMER	No
28285	RECONSTRUCTION TOE HAMMER	No
28289	CORRECT HALLUX RIGIDUS W/ CHEILECTOMY,DEBRIDEMENT, CAPSULAR RELEASE 1ST MTJ	No
28296	BUNIONECTOMY W/DISTAL METATARSAL OSTEOTOMY,ANY METHOD	No
28296	BUNIONECTOMY W/DISTAL METATARSAL OSTEOTOMY,ANY METHOD	No
28296	BUNIONECTOMY W/DISTAL METATARSAL OSTEOTOMY,ANY METHOD BILATERAL	No
28485	ORIF METATARSAL	No
28485	ORIF METATARSAL	No
28485	ORIF TOE(S)	No
28615	ORIF FOOT TARSOMETATARSAL INCLUDES INTERNAL FIXATION	No
28615	ORIF FOOT TARSOMETATARSAL INCLUDES INTERNAL FIXATION	No
28615	ORIF TARSOMETATARSAL JOINT OR LISFRANC FRACTURE DISLOCATION	No
28725	ARTHRODESIS SUBTALAR	No
28730	ARTHRODESIS MIDTARSAL MULTIPLE/TRANSVERSE	No
28730	ARTHRODESIS MIDTARSAL MULTIPLE/TRANSVERSE	No
28730	ARTHRODESIS MIDTARSAL OR TARSOMETATARSAL, MULTIPLE OR TRANSVERSE	No
28730	ARTHRODESIS TARSOMETATARSAL MULTIPLE/TRANSVERSE	No
28750	ARTHRODESIS GREAT TOE METATARSOPHALANGEAL JOINT	No
28750	ARTHRODESIS GREAT TOE METATARSOPHALANGEAL JOINT	No
28750	ARTHRODESIS SINGLE JOINT TOE(S)	No
28805	AMPUTATION FOOT TRANSMETATARSAL	No
28805	AMPUTATION FOOT TRANSMETATARSAL	No

MSBOS - All records

Individual patient parameters (i.e., anemia, coagulopathy, transfusion risk) should be taken into consideration and may warrant an individual TSCR when not generally recommended.

June 22, 2023 **Sorted by CPT**

Primary CPT	Procedure Name	TSCR (Type and Screen) Needed?
28805	AMPUTATION REVISION FOOT TRANSMETATARSAL	No
28805	AMPUTATION TRANSMETATARSAL	No
28810	AMPUTATION REVISION TOE(S)	No
28810	AMPUTATION TOE(S)	No
28810	AMPUTATION TOE(S)	No
28810	AMPUTATION TRANSMETATARSAL W/ TOE	No
28820	AMPUTATION TOE METATARSOPHALANGEAL JOINT	No
28825	AMPUTATION TOE INTERPHALANGEAL JOINT	No
29806	ARTHROSCOPY SHOULDER W/ CAPSULORRHAPHY	No
29807	ARTHROSCOPIC DECOMPRESSION SHOULDER AND SLAP REPAIR	No
29807	ARTHROSCOPIC DECOMPRESSION SHOULDER AND SLAP REPAIR	No
29807	SHOULDER ARTHROSCOPY W/ REPAIR SLAP LESION	No
29822	ARTHROSCOPY SHOULDER W/ DEBRIDEMENT LIMITED 1 OR 2 DISCRETE STRUCTURES	No
29823	ARTHROSCOPY SHOULDER W/ DEBRIDEMENT EXTENSIVE 3 OR MORE DISCRETE STRUCTURES	No
29827	ARTHROSCOPY SHOULDER ROTATOR CUFF	No
29828	ARTHROSCOPY SHOULDER BICEPS TENODESIS	No
29848	ENDOSCOPIC RELEASE TUNNEL CARPAL	No
29848	ENDOSCOPIC RELEASE TUNNEL CARPAL	No
29848	ENDOSCOPY WRIST W/ RELEASE OF TRANSVERSE CARPAL LIGAMENT	No
29877	ARTHROSCOPY KNEE ARTICULAR CARTILAGE SHAVING OR DEBRIDEMENT	No
29877	ARTHROSCOPY KNEE ARTICULAR CARTILAGE SHAVING OR DEBRIDEMENT	No
29877	CHONDROPLASTY KNEE	No
29880	ARTHROSCOPY KNEE MENISCECTOMY MEDIAL AND LATERAL WITH MENISCAL SHAVING	No
29881	ARTHROSCOPY KNEE MENISCECTOMY MEDIAL OR LATERAL	No
29881	ARTHROSCOPY KNEE MENISCECTOMY MEDIAL OR LATERAL	No
29881	ARTHROSCOPY KNEE MENISCECTOMY MEDIAL OR LATERAL BILATERAL	No
29881	ARTHROSCOPY KNEE W/ MENISCECTOMY LATERAL	No
29881	ARTHROSCOPY KNEE W/ MENISCECTOMY MEDIAL	No
29881	ARTHROSCOPY KNEE WITH MENISCECTOMY MEDIAL OR LATERAL AND MENISCAL SHAVING	No
29882	ARTHROSCOPY KNEE W/ MENISCUS REPAIR LATERAL	No
29882	ARTHROSCOPY KNEE W/ MENISCUS REPAIR MEDIAL	No
29882	ARTHROSCOPY, KNEE MENISCUS REPAIR MEDIAL OR LATERAL	No
29882	ARTHROSCOPY, KNEE MENISCUS REPAIR MEDIAL OR LATERAL	No
29888	ARTHROSCOPIC REPAIR LIGAMENT ANTERIOR CRUCIATE	No
29888	ARTHROSCOPIC REPAIR LIGAMENT ANTERIOR CRUCIATE	No
29888	ARTHROSCOPIC REPAIR LIGAMENT ANTERIOR CRUCIATE WITH GRAFT HAMSTRING	No
29888	ARTHROSCOPY KNEE ANTERIOR CRUCIATE LIGAMENT REPAIR AUGMENTATION OR RECONSTRUCTION	No

MSBOS - All records

Individual patient parameters (i.e., anemia, coagulopathy, transfusion risk) should be taken into consideration and may warrant an individual TSCR when not generally recommended.
June 22, 2023 **Sorted by CPT**

Primary CPT	Procedure Name	TSCR (Type and Screen) Needed?
29888	ARTHROSCOPY KNEE ANTERIOR CRUCIATE LIGAMENT REPAIR AUGMENTATION OR RECONSTRUCTION PEDIATRIC	No
29916	ARTHROSCOPY HIP W/ LABRAL REPAIR	No
30140	RESECTION SUBMUCOSAL TURBINATES	No
30140	RESECTION SUBMUCOSAL TURBINATES	No
30140	TURBINECTOMY	No
30420	RHINOPLASTY OPEN	No
30420	RHINOPLASTY PRIMARY INCLUDING MAJOR SEPTAL REPAIR	No
30420	RHINOPLASTY PRIMARY INCLUDING MAJOR SEPTAL REPAIR	No
30420	SEPTORHINOPLASTY	No
30420	SEPTORHINOPLASTY PEDIATRIC	No
30465	REPAIR NASAL VESTIBULAR STENOSIS	No
30520	ENDOSCOPIC SEPTOPLASTY	No
30520	ENDOSCOPIC SEPTOPLASTY PEDIATRIC	No
30520	SEPTOPLASTY	No
30520	SEPTOPLASTY	No
30520	SEPTOPLASTY PEDIATRIC	No
30520	SUBMUCOUS RESECTION NASAL SEPTUM W/ GRAFT	No
30520	SUBMUCOUS RESECTION NASAL SEPTUM W/O GRAFT	No
31237	ENDOSCOPY NASAL/SINUS W/ BIOPSY	No
31237	ENDOSCOPY NASAL/SINUS W/ BIOPSY	No
31237	ENDOSCOPY NASAL/SINUS W/ DEBRIDEMENT	No
31237	NASAL SINUS ENDOSCOPY SURGICAL WITH BIOPSY POLYPECTOMY OR DEBRIDEMENT	No
31237	POLYPECTOMY NASAL	No
31239	ENDOSCOPY NASAL/SINUS W/ DACROCYSTORHINOSTOMY	No
31259	NASAL/SINUS ENDOSCOPY,SURGICAL,W/ETHMOIDECTOMY TOTAL,INCLUDING SPHENOIDOTOMY W/SPHENOID SINIUS TISSUE REMOVAL	No
31267	ENDOSCOPY NASAL/SINUS W/ SPHENOIDOTOMY AND REMOVAL MAXILLARY SINUS TISSUE	No
31267	NASAL/SINUS ENDOSCOPY SURGICAL W/ MAXILLARY ANTROSTOMY W/ REMOVAL OF TISSUE FROM MAXILLARY SINUS	No
31267	NASAL/SINUS ENDOSCOPY SURGICAL W/ MAXILLARY ANTROSTOMY W/ REMOVAL OF TISSUE FROM MAXILLARY SINUS	No
31276	ENDOSCOPY NASAL/SINUS W/ FRONTAL SINUS EXPLORATION	No
31276	ENDOSCOPY NASAL/SINUS W/ FRONTAL SINUS EXPLORATION	No
31276	ENDOSCOPY NASAL/SINUS W/ FRONTAL SINUS EXPLORATION PEDIATRIC	No
31525	LARYNGOSCOPY DIRECT	No
31525	LARYNGOSCOPY DIRECT	No
31525	LARYNGOSCOPY DIRECT PEDIATRIC	No
31526	LARYNGOSCOPY MICRO	No
31526	LARYNGOSCOPY MICRO PEDIATRIC	No
31526	LARYNGOSCOPY MICRO PEDIATRIC	No

MSBOS - All records

Individual patient parameters (i.e., anemia, coagulopathy, transfusion risk) should be taken into consideration and may warrant an individual TSCR when not generally recommended.

June 22, 2023 **Sorted by CPT**

Primary CPT	Procedure Name	TSCR (Type and Screen) Needed?
31528	LARYNGOSCOPY DIRECT, WITHOUT TRACHEOSCOPY; WITH DILATION, INITIAL	No
31528	LARYNGOSCOPY DIRECT, WITH TRACHEOSCOPY; WITH DILATION, INITIAL	No
31528	LARYNGOSCOPY DIRECT, WITH TRACHEOSCOPY; WITH DILATION, INITIAL	No
31529	LARYNGOSCOPY DIRECT, WITH TRACHEOSCOPY; WITH DILATION, SUBSEQUENT	No
31529	LARYNGOSCOPY DIRECT, WITHOUT TRACHEOSCOPY; WITH DILATION, SUBSEQUENT	No
31529	LARYNGOSCOPY DIRECT, WITHOUT TRACHEOSCOPY; WITH DILATION, SUBSEQUENT	No
31535	LARYNGOSCOPY W/ BIOPSY	No
31536	LARYNGOSCOPY DIRECT W/ BIOPSY	No
31541	LARYNGOSCOPY DIRECT W/ EXCISION OF TUMOR	No
31541	LARYNGOSCOPY DIRECT W/ EXCISION OF TUMOR & STRIP EPIGLOTTIS W/ MICROSCOPE	No
31541	LARYNGOSCOPY DIRECT W/ EXCISION OF TUMOR & STRIP VOCAL CORD & EPIGLOTTIS W/ MICROSCOPE	No
31541	LARYNGOSCOPY DIRECT W/ EXCISION OF TUMOR & STRIP VOCAL CORDS W/ MICROSCOPE	No
31541	LARYNGOSCOPY DIRECT W/ EXCISION OF TUMOR W/ MICROSCOPE	No
31541	LARYNGOSCOPY DIRECT W/ STRIPPING EPIGLOTTIS W/ MICROSCOPE	No
31541	MICROLARYNGOSCOPY DIRECT W/ EXCISION OF VOCAL CORD POLYP	No
31545	LARYNGOSCOPY, DIRECT, OPERATIVE	No
31571	LARYNGOSCOPY DIRECT W/ VOCAL CORD INJECTION	No
31591	LARYNGOPLASTY,MEDIALIZATION,UNILATERAL	No
31600	TRACHEOSTOMY	No
31600	TRACHEOSTOMY ADULT	No
31600	TRACHEOSTOMY PEDIATRIC	No
32608	ROBOTIC THORACOSCOPY W/ DIAGNOSTIC BIOPSY(IES) OF LUNG INFILTRATE(S) UNILATERAL	No
32608	ROBOTIC THORACOSCOPY W/ DIAGNOSTIC BIOPSY(IES) OF LUNG NODULE(S) OR MASS(ES) UNILATERAL	No
32608	THORACOSCOPY W/ DIAGNOSTIC BIOPSY(IES)	No
32608	THORACOSCOPY W/ DIAGNOSTIC BIOPSY(IES) OF LUNG NODULE(S) OR MASS(ES) UNILATERAL	No
32652	THORACOSCOPY W/ TOTAL PULMONARY DECORTICATION	Yes
32655	ROBOTIC THORACOSCOPY SURGICAL W/RESECTION-PLICATION OF BULLAE	No
32655	THORACOSCOPY W/ EXCISION OF BULLAE	No
32655	THORACOSCOPY W/ EXCISION OF BULLAE INCLUDING PLEURAL PROCEDURE	No
32655	XI ROBOTIC THORACOSCOPY SURGICAL W/RESECTION-PLICATION OF BULLAE	No
32663	LOBECTOMY LUNG WITH THORACOSCOPY	Yes
32666	ROBOTIC THORASCOPY SURGICAL W/THERAPEUTIC WEDGE RESECTION INITIAL UNILATERAL	No
32666	SI ROBOTIC THORASCOPY SURGICAL W/THERAPEUTIC WEDGE RESECTION INITIAL UNILATERAL	No
32666	THORASCOPY SURGICAL W/THERAPEUTIC WEDGE RESECTION	No
32666	THORASCOPY SURGICAL W/THERAPEUTIC WEDGE RESECTION INITIAL UNILATERAL	No
32666	XI ROBOTIC THORASCOPY SURGICAL W/THERAPEUTIC WEDGE RESECTION INITIAL UNILATERAL	No
32669	THORACOSCOPY SURGICAL W/REMOVAL OF A SINGLE LUNG SEGMENT	Yes

MSBOS - All records

Individual patient parameters (i.e., anemia, coagulopathy, transfusion risk) should be taken into consideration and may warrant an individual TSCR when not generally recommended.

June 22, 2023 **Sorted by CPT**

Primary CPT	Procedure Name	TSCR (Type and Screen) Needed?
32673	ROBOTIC THORACOSCOPY SURGICAL W/ RESECTION OF THYMUS UNILATERAL OR BILATERAL	No
32673	ROBOTIC THORACOSCOPY SURGICAL W/ RESECTION OF THYMUS UNILATERAL OR BILATERAL	No
32673	THORACOSCOPY SURGICAL W/RESECTION OF THYMUS BILATERAL	No
32673	THORACOSCOPY SURGICAL W/RESECTION OF THYMUS UNILATERAL	No
32673	XI ROBOTIC THORACOSCOPY SURGICAL W/ RESECTION OF THYMUS UNILATERAL OR BILATERAL	No
32854	LUNG TRANSPLANT BILATERAL W/ CARDIOPULMONARY BYPASS	Yes
33025	CREATION OF PERICARDIAL WINDOW OR PARTIAL RESECTION FOR DRAINAGE ANTEROLATERAL	Yes
33025	WINDOW PERICARDIAL	Yes
33025	WINDOW PERICARDIAL	Yes
33031	PERICARDIECTOMY W/BYPASS	Yes
33120	EXCISION OF INTRACARDIAC TUMOR VIA RESECTION W/ BY-PASS	Yes
33120	EXCISION OF INTRACARDIAC TUMOR VIA RESECTION W/ BY-PASS	Yes
33120	ROBOTIC EXCISION OR RESECTION, INTRACARDIAC TUMOR, WITH CARDIOPULMONARY BYPASS	Yes
33120	XI ROBOTIC EXCISION OR RESECTION, INTRACARDIAC TUMOR, WITH CARDIOPULMONARY BYPASS	Yes
33259	FULL MAZE DONE W/ CABG W/ CP-BYPASS	Yes
33259	FULL MAZE DONE W/ VALVE REPAIR W/ CP-BYPASS	Yes
33259	FULL MAZE DONE W/ VALVE REPLACEMENT W/ CP-BYPASS	Yes
33259	RECONSTRUCTION ATRIA W/ABLATION EXTENSIVE, W/ OTHER CARDIAC PROCEDURE, W/ BYPASS	Yes
33259	RECONSTRUCTION ATRIA W/ABLATION EXTENSIVE, W/ OTHER CARDIAC PROCEDURE, W/ BYPASS	Yes
33391	OPEN COMPLEX AORTIC VALVULOPLASTY W/CP BYPASS	Yes
33391	OPEN COMPLEX AORTIC VALVULOPLASTY W/CP BYPASS	Yes
33391	OPEN COMPLEX VALVULOPLASTY W/CP BYPASS	Yes
33391	OPEN SIMPLE AORTIC COMPLEX VALVULOPLASTY W/CP BYPASS 10-24.9 KG	Yes
33405	AVR W/ CARDIOPULMONARY BYPASS W/ PROSTHETIC OTHER THAN HOMOGRAFT/ STENTLESS TISSUE VALVE	Yes
33405	AVR W/ CARDIOPULMONARY BYPASS W/ PROSTHETIC OTHER THAN HOMOGRAFT/ STENTLESS TISSUE VALVE	Yes
33405	AVR W/ CP BYPASS W/ PROSTHETIC VALVE OTHER THAN HOMOFRAFT <2.5KG	Yes
33405	AVR W/ CP BYPASS W/ PROSTHETIC VALVE OTHER THAN HOMOGRAFT 5.0-9.9KG	Yes
33411	AVR W/ AORTIC ANNULUS ENLARGEMENT	Yes
33416	VENTRICULOMYOMECTOMY FOR IDIOPATHIC HYPERTROPHIC SUBAORTIC STENOSIS	Yes
33416	VENTRICULOMYOTOMY FOR IDEOPATHIC HYPERTROPHIC SUBAORTIC STENOSIS	Yes
33416	VENTRICULOMYOTOMY FOR IDEOPATHIC HYPERTROPHIC SUBAORTIC STENOSIS	Yes
33418	TRANSCATHETER MITRAL VALVE REPAIR PERCUTANEOUS INCLUDE TRANSSEPTAL PUNCTURE WHEN PERFORMED INITIAL PROSTHESIS	No
33426	MIN INVASIVE MITRAL VALVOPLASTY W/ CP BYPASS W/ PROSTHETIC RING	Yes
33426	ROBOTIC MIN INVASIVE MITRAL VALVOPLASTY W/ CP BYPASS W/ PROSTHETIC RING	Yes
33426	VALVULOPLASTY MITRAL W/ RING AND BYPASS	Yes
33426	VALVULOPLASTY MITRAL W/ RING AND BYPASS	Yes
33427	VALVULOPLASTY MITRAL RADICAL W/ BYPASS	Yes

MSBOS - All records

Individual patient parameters (i.e., anemia, coagulopathy, transfusion risk) should be taken into consideration and may warrant an individual TSCR when not generally recommended.

June 22, 2023 **Sorted by CPT**

Primary CPT	Procedure Name	TSCR (Type and Screen) Needed?
33427	VALVULOPLASTY MITRAL RADICAL W/ BYPASS	Yes
33427	VALVULOPLASTY MITRAL RADICAL W/ BYPASS PEDIATRIC	Yes
33430	MINIMALLY INVASIVE MITRAL VALVE REPLACEMENT W/ CP BYPASS PEDIATRIC	Yes
33430	REPLACEMENT MITRAL VALVE W/ CARDIOPULMONARY BYPASS	Yes
33430	REPLACEMENT MITRAL VALVE W/ CARDIOPULMONARY BYPASS	Yes
33464	MINIMALLY INVASIVE VALVULOPLASTY TRICUSPID VALVE W/ RING INSERTION W/ CPB	Yes
33464	TVR VALVULOPLASTY TRICUSPID VALVE W/ RING INSERTION	Yes
33464	TVR VALVULOPLASTY TRICUSPID VALVE W/ RING INSERTION	Yes
33464	VALVULOPLASTY TRICUSPID W/ RING INSERTION, PEDIATRIC	Yes
33465	REPLACEMENT TRICUSPID W/ BYPASS	Yes
33465	REPLACEMENT TRICUSPID W/ BYPASS	Yes
33465	REPLACEMENT TRICUSPID W/ BYPASS PEDIATRIC	Yes
33507	RPR OF ANOMALOUS AORTIC ORIGIN OF CORONARY ARTERY	No
33507	RPR OF ANOMALOUS AORTIC ORIGIN OF CORONARY ARTERY BY TRANSLOCATION	No
33507	RPR OF ANOMALOUS AORTIC ORIGIN OF CORONARY ARTERY BY UNROOFING	No
33507	RPR OF ANOMOLOUS AORTIC ORIGIN OF CORONARY ARTERY BY TRANSLOCATION PEDIATRIC	No
33507	RPR OF ANOMOLOUS AORTIC ORIGIN OF CORONARY ARTERY BY UNROOFING PEDIATRIC	No
33508	ENDOSCOPIC HARVEST VEIN FOR CORONARY ARTERY BYPASS PROCEDURE	Yes
33508	ENDOSCOPIC HARVEST VEIN FOR CORONARY ARTERY BYPASS PROCEDURE	Yes
33508	HARVEST VEIN, VIDEO-ASSISTED VIA ENDOSCOPY FOR BYPASS, SEPARATE PROCEDURE	Yes
33517	BYPASS GRAFT ARTERY CORONARY OFF PUMP VENOUS GRAFT(S) AND ARTERIAL GRAFT(S) SINGLE VEIN GRAFT	Yes
33517	BYPASS GRAFT ARTERY CORONARY ON-PUMP USING VENOUS GRAFT(S) AND ARTERIAL GRAFT(S) SINGLE VEIN GRAFT	Yes
33517	BYPASS GRAFT ARTERY CORONARY USING VENOUS GRAFT(S) AND ARTERIAL GRAFT(S) SINGLE VEIN GRAFT	Yes
33518	BYPASS GRAFT ARTERY CORONARY OFF PUMP VENOUS GRAFT(S) AND ARTERIAL GRAFT(S) TWO VENOUS GRAFTS	Yes
33518	BYPASS GRAFT ARTERY CORONARY ON-PUMP USING VENOUS GRAFT(S) AND ARTERIAL GRAFT(S); TWO VENOUS GRAFTS	Yes
33518	BYPASS GRAFT ARTERY CORONARY USING VENOUS GRAFT(S) AND ARTERIAL GRAFT(S); TWO VENOUS GRAFTS	Yes
33519	BYPASS GRAFT ARTERY CORONARY USING VENOUS GRAFT(S) AND ARTERIAL GRAFT(S) THREE VENOUS GRAFTS	Yes
33519	BYPASS GRAFT ARTERY CORONARY OFF PUMP VENOUS GRAFT(S) AND ARTERIAL GRAFT(S) THREE VENOUS GRAFTS	Yes
33519	BYPASS GRAFT ARTERY CORONARY ON-PUMP USING VENOUS GRAFT(S) AND ARTERIAL GRAFT(S) THREE VENOUS GRAFTS	Yes
33530	BYPASS ARTERY CORONARY REDO ON PUMP	Yes
33530	BYPASS ARTERY CORONARY REDO ON PUMP	Yes
33530	REDO AORTIC VALVE REPAIR/REPLACEMENT-ON PUMP	Yes
33530	REDO OFF PUMP CORONARY ARTERY BYPASS PROCEDURE OR VALVE PROCEDURE	Yes
33533	BYPASS GRAFT ARTERY CORONARY SINGLE CORONARY ARTERIAL GRAFT	Yes
33533	BYPASS GRAFT ARTERY CORONARY OFF PUMP SINGLE ARTERIAL GRAFT	Yes
33533	BYPASS GRAFT ARTERY CORONARY OFF PUMP SINGLE CORONARY VENOUS GRAFT	Yes
33533	BYPASS GRAFT ARTERY CORONARY ON-PUMP SINGLE CORONARY ARTERIAL GRAFT	Yes

MSBOS - All records

Individual patient parameters (i.e., anemia, coagulopathy, transfusion risk) should be taken into consideration and may warrant an individual TSCR when not generally recommended.

June 22, 2023 **Sorted by CPT**

Primary CPT	Procedure Name	TSCR (Type and Screen) Needed?
33534	BYPASS GRAFT ARTERY CORONARY OFF PUMP TWO ARTERIAL GRAFTS	Yes
33534	BYPASS GRAFT ARTERY CORONARY ON-PUMP TWO CORONARY ARTERIAL GRAFTS	Yes
33534	BYPASS GRAFT ARTERY CORONARY TWO CORONARY ARTERIAL GRAFTS	Yes
33858	ASCENDING AORTA GRAFT WITH CARDIOPULMONARY BYPASS VIA "J" INCISION (MIS) FOR DISSECTION	Yes
33858	GRAFT ASCENDING AORTA W/VALVE SUSPENSION W/CARDIOPULMONARY BYPASS FOR AORTIC DISSECTION	Yes
33858	GRAFT ASCENDING AORTA W/VALVE SUSPENSION W/CARDIOPULMONARY BYPASS FOR AORTIC DISSECTION	Yes
33859	GRAFT ASCENDING AORTA W/VALVE SUSPENSION W/CARDIOPULMONARY BYPASS FOR AORTIC DISEASE OTHER THAN DISSECTION	Yes
33863	GRAFT ASCENDING AORTA, AORTIC ROOT REPLACEMENT W/ PROSTHESIS & CORONARY RECONSTRUCT W/ CARDIOPULMONARY BYPASS	Yes
33864	ASCENDING AORTA GRAFT, W/CPB W/VALVE SUSPENSION	Yes
33871	TRANSVERSE ARCH GRAFT,W/ CARDIOPULMONARY BYPASS	Yes
33877	REPAIR THORACOABDOMINAL AORTIC ANEURYSM W/ GRAFT	Yes
33880	ENDOVASC RPR DESCEND THORC AORTA W/ LT SUBCLAV ART W/ INI ENDOPROSTHESIS	Yes
33881	ENDOVASCULAR REPAIR DESCENDING THORACIC AORTA W/ ENDOPROSTHESIS	Yes
33886	PLACE DISTAL EXTENSION PROSTHESIS IN DESCENDING THORACIC AORTA DELAYED AFTER ENDOVASC REPAIR	Yes
33886	PLACE DISTAL EXTENSION PROSTHESIS IN DESCENDING THORACIC AORTA DELAYED AFTER ENDOVASC REPAIR	Yes
33886	THORACIC AORTA DISTAL GRAFT EXTENSION PLACEMENT,DELAYED	Yes
33916	PULM. ENDARTERECTOMY W/EMBOLECTOMY, W/CPB PEDIATRIC	Yes
33916	PULMONARY ENDARTERECTOMY W/ CARDIOPULMONARY BYPASS	Yes
33916	PULMONARY ENDARTERECTOMY W/ CARDIOPULMONARY BYPASS	Yes
33945	HEART TRANSPLANT W/ RECIPIENT CARDIECTOMY	Yes
33945	HEART TRANSPLANT W/ RECIPIENT CARDIECTOMY	Yes
33945	HEART TRANSPLANT W/ RECIPIENT CARDIECTOMY 10KG - 24.9KG	Yes
33945	HEART TRANSPLANT W/O RECIPIENT CARDIECTOMY 10KG - 24.9KG	Yes
33952	PERC INSERTION PERIPHERAL CANNULA(E) FOR ECMO/ECLS 6 YEARS AND OLDER (INCLUDES FLUORO WHEN PERFORMED)	Yes
33979	INSERTION VAD, IMPLANTABLE INTRACORPOREAL, SINGLE VENTRICLE	Yes
33990	INSERTION OF VENTRICULAR ASSIST DEVICE PERCUTANEOUS W/ RADIOLOGICAL SUPERVISION AND INTERPRETATION LEFT HEART ARTERIAL ACCESS ONLY	Yes
33992	REMOVAL OF PERC LEFT HEART VENTRICULAR ASSIST DEVICE ARTERIAL OR ARTERIAL AND VENOUS CANNULA(S)	Yes
34201	EMBOLECTOMY AORTOILIAC ARTERY VIA LEG INCISION	Yes
34201	EMBOLECTOMY FEMORAL	Yes
34201	EMBOLECTOMY FEMORAL	Yes
34201	EMBOLECTOMY FEMORAL ILIAC	Yes
34201	EMBOLECTOMY FEMORAL POPLITEAL	Yes
34201	EMBOLECTOMY FEMOROPOPLITEAL ARTERY VIA LEG INCISION	Yes
34201	EVACUATION CLOT ARTERIAL INCISION FEMOROPOPLITEAL AORTOILIAC	Yes
34201	THROMBECTOMY AORTOILIAC ARTERY VIA LEG INCISION	Yes
34201	THROMBECTOMY FEMOROPOPLITEAL ARTERY VIA LEG INCISION	Yes

MSBOS - All records

Individual patient parameters (i.e., anemia, coagulopathy, transfusion risk) should be taken into consideration and may warrant an individual TSCR when not generally recommended.

June 22, 2023 **Sorted by CPT**

Primary CPT	Procedure Name	TSCR (Type and Screen) Needed?
34705	ENDOVASCULAR REPAIR RENAL TO ILIAC LEVEL,W/AORTO-BILATERAL-ILIAC ENDOGRAFT,WITHOUT RUPTURE	Yes
35081	REPAIR ANEURYSM AORTIC ABDOMINAL	Yes
35081	REPAIR ANEURYSM AORTIC ABDOMINAL	Yes
35081	RESECTION ANEURYSM AORTIC ABDOMINAL	Yes
35091	DIRECT REPAIR OF ABDOMINAL AORTIC ANEURYSM	Yes
35091	DIRECT REPAIR OF ABDOMINAL AORTIC ANEURYSM INVOLVING CELIAC ARTERY	Yes
35091	DIRECT REPAIR OF ABDOMINAL AORTIC ANEURYSM INVOLVING MESENTERIC ARTERY	Yes
35091	DIRECT REPAIR OF ABDOMINAL AORTIC ANEURYSM INVOLVING RENAL ARTERY	Yes
35091	DIRECT REPAIR OF SUPRARENAL ABDOMINAL AORTIC ANEURYSM	Yes
35301	ENDARTERECTOMY CAROTID ADULT	Yes
35301	ENDARTERECTOMY CAROTID ADULT	Yes
35301	ENDARTERECTOMY CAROTID VEIN PATCH GRAFT	Yes
35301	ENDARTERECTOMY CAROTID WITH PATCH ANGIOPLASTY	Yes
35355	THROMBOENDARTERECTOMY W/O GRAFT; ILIOFEMORAL	Yes
35355	THROMBOENDARTERECTOMY, ILIOFEMORAL	Yes
35355	THROMBOENDARTERECTOMY,W/ PATCH GRAFT; ILIOFEMORAL	Yes
35371	ENDARTERECTOMY FEMORAL	Yes
35371	ENDARTERECTOMY FEMORAL	Yes
35371	ENDARTERECTOMY FEMORAL WITH ANGIOPLASTY	Yes
35371	ENDARTERECTOMY FEMORAL WITH VEIN PATCH ANGIOPLASTY	Yes
35372	ENDARTERECTOMY FEMORAL PROFUNDA	Yes
35556	BYPASS ARTERY FEMORAL	Yes
35556	BYPASS ARTERY FEMORAL POPLITEAL	Yes
35556	BYPASS ARTERY FEMORAL POPLITEAL WITH SAPHENOUS VEIN	Yes
35556	BYPASS ARTERY FEMORAL TIBIAL	Yes
35556	BYPASS GRAFT REVERSE VEIN FEMORAL POPLITEAL	Yes
35566	BYPASS ARTERY FEMORAL DORSALIS PEDIS	Yes
35566	BYPASS ARTERY FEMORAL PERONEAL	Yes
35566	BYPASS ARTERY POSTERIOR TIBIAL WITH CRYOVEIN SAPHENOUS VEIN	Yes
35566	BYPASS ARTERY POSTERIOR TIBIAL WITH EVH (ENDOSCOPIC VEIN HARVEST)	Yes
35566	BYPASS ARTERY POSTERIOR TIBIAL WITH VEIN	Yes
35566	BYPASS GRAFT REVERSE VEIN FEMORAL PERONEAL	Yes
35566	BYPASS GRAFT REVERSE VEIN FEMORAL TIBIAL	Yes
35566	BYPASS GRAFT REVERSE VEIN FEMORAL TIBIAL	Yes
35606	BYPASS SUBCLAVIAN-CAROTID ARTERY W/ SYNTHETIC GRAFT	No
35703	EXPLORATION ARTERY	No
35703	EXPLORATION ARTERY FEMORAL	No

MSBOS - All records

Individual patient parameters (i.e., anemia, coagulopathy, transfusion risk) should be taken into consideration and may warrant an individual TSCR when not generally recommended.

June 22, 2023 **Sorted by CPT**

Primary CPT	Procedure Name	TSCR (Type and Screen) Needed?
35703	EXPLORATION ARTERY POPLITEAL	No
35703	EXPLORATION NOT FOLLOWED BY SURGICAL REPAIR ARTERY LOWER EXTREMITY	No
35800	EXPLORE POST OPERATIVE NECK	Yes
35820	EXPLORATION FOR POSTOPERATIVE HEMORRHAGE/THROMBOSIS/INFECTION CHEST	Yes
35840	EXPLORATION ABDOMINAL VESSELS	Yes
35840	EXPLORATION FOR POSTOPERATIVE HEMORRHAGE, THROMBOSIS OR INFECTION ABDOMEN	Yes
35840	EXPLORATION FOR POSTOPERATIVE HEMORRHAGE, THROMBOSIS OR INFECTION ABDOMEN	Yes
35860	EXPLORATION FOR POSTOPERATIVE HEMORRHAGE, THROMBOSIS OR INFECTION; EXTREMITY	Yes
35903	REMOVAL BYPASS GRAFT AXILLARY BRACHIAL SAPHENOUS	Yes
35903	REMOVAL BYPASS GRAFT AXILLO FEMORAL	Yes
35903	REMOVAL BYPASS GRAFT ILEO FEMORAL	Yes
35903	REMOVAL GRAFT	Yes
35903	REMOVAL GRAFT ARTERIOVENOUS EXTREMITY LOWER	Yes
35903	REMOVAL GRAFT ARTERIOVENOUS EXTREMITY UPPER	Yes
36221	NON-SELECTIVE CATH PLACEMENT THORACIC AORTA W/ ANGIOGRAPHY OF THE EXTRACRANIAL CAROTID VERTEBRAL AND/OR INTRACRANIAL VESSELS BILATERAL W/ ANGIOGRAPHY OF THE CERVICOCEREBRAL ARCH	No
36221	NON-SELECTIVE CATH PLACEMENT THORACIC AORTA W/ ANGIOGRAPHY OF THE EXTRACRANIAL CAROTID VERTEBRAL AND/OR INTRACRANIAL VESSELS UNILATERAL W/ ANGIOGRAPHY OF THE CERVICOCEREBRAL ARCH	No
36221	NON-SELECTIVE CATH PLACEMENT THORACIC AORTA W/ ANGIOGRAPHY OF THE EXTRACRANIAL CAROTID VERTEBRAL AND/OR INTRACRANIAL VESSELS UNILATERAL W/ ANGIOGRAPHY OF THE CERVICOCEREBRAL ARCH	No
36222	SELECTIVE CATH PLACEMENT COMMON CAROTID OR INNOMINATE ARTERY BILATERAL W/ ANGIOGRAPHY OF THE IPSILATERAL EXTRACRANIAL CAROTID CIRCULATION AND ALL ASSOCIATED W/ ANGIOGRAPHY OF THE CERVICOCEREBRAL ARCH	No
36222	SELECTIVE CATH PLACEMENT COMMON CAROTID OR INNOMINATE ARTERY BILATERAL W/ ANGIOGRAPHY OF THE IPSILATERAL EXTRACRANIAL CAROTID CIRCULATION AND ALL ASSOCIATED W/ ANGIOGRAPHY OF THE CERVICOCEREBRAL ARCH	No
36222	SELECTIVE CATH PLACEMENT COMMON CAROTID OR INNOMINATE ARTERY UNILATERAL W/ ANGIOGRAPHY OF THE IPSILATERAL EXTRACRANIAL CAROTID CIRCULATION AND ALL ASSOCIATED W/ ANGIOGRAPHY OF THE CERVICOCEREBRAL ARCH	No
36245	ANGIOGRAM RENAL	No
36245	ANGIOGRAM SUBCLAVIAN	No
36245	ARTERIOGRAM CELIAC	No
36245	ARTERIOGRAM INFERIOR MESENTRIC	No
36245	ARTERIOGRAM SUPERIOR MESENTRIC	No
36245	SELECTIVE CATHETER PLACEMENT 1ST ORD LOWER VASC FAMILY FOR ARTERIOGRAM	No
36245	SELECTIVE CATHETER PLACEMENT ARTERIAL SYSTEM EA 1ST ORDER ABD/PELVIC/LOWER EXTREMITY ARTERY BRANCH	No
36245	SELECTIVE CATHETER PLACEMENT ARTERIAL SYSTEM EA 1ST ORDER ABD/PELVIC/LOWER EXTREMITY ARTERY BRANCH	No
36246	ANGIOGRAM EXTREMITY LOWER	Yes
36246	ANGIOGRAM EXTREMITY LOWER	Yes
36246	ANGIOGRAM MESENTERIC	Yes

MSBOS - All records

Individual patient parameters (i.e., anemia, coagulopathy, transfusion risk) should be taken into consideration and may warrant an individual TSCR when not generally recommended.
June 22, 2023 **Sorted by CPT**

Primary CPT	Procedure Name	TSCR (Type and Screen) Needed?
36246	SELECTIVE CATHETER PLACEMENT 2ND ORD LOWER VASC FAMILY FOR ARTERIOGRAM	Yes
36246	SELECTIVE CATHETER PLACEMENT ARTERIAL SYSTEM INITIAL 2ND ORDER ABD/PELVIC/LOWER EXTREMITY ARTERY BRANCH	Yes
36247	EMBOLIZATION COIL EXTREMITY LOWER	No
36247	SELECTIVE CATHETER PLACEMENT 3RD ORDER LOWER VASC FAMILY FOR ARTERIOGRAM	No
36247	SELECTIVE CATHETER PLACEMENT ARTERIAL SYSTEM INITIAL 3RD ORDER OR MORE SELECTIVE ABD/PELVIC/LOWER EXTREMITY ARTERY BRANCH	No
36247	SELECTIVE CATHETER PLACEMENT ARTERIAL SYSTEM INITIAL 3RD ORDER OR MORE SELECTIVE ABD/PELVIC/LOWER EXTREMITY ARTERY BRANCH	No
36558	INSERTION CATHETER BROVIAC WITH C-ARM	No
36558	INSERTION CATHETER HICKMAN	No
36558	INSERTION OF TUNNELED CENTRALLY INSERTED CENTRAL VENOUS CATHETER W/O SUBQ PORT OR PUMP AGE 5YEARS OR OLDER	No
36558	INSERTION OF TUNNELED CENTRALLY INSERTED CENTRAL VENOUS CATHETER W/O SUBQ PORT OR PUMP AGE 5YEARS OR OLDER	No
36558	INSERTION TUNNELED CV CATHETER	No
36561	INSERTION CATHETER PORT-A-CATH WITH C-ARM	No
36561	INSERTION CATHETER PORT-A-CATH WITH C-ARM	No
36561	INSERTION CV CATHETER WITH PORT, OVER 4 YRS.	No
36561	INSERTION PORT VENOUS ACCESS ADULT	No
36561	INSERTION TUNNELED CENTRAL VENOUS ACCESS DEVICE W/ SUBCUTANEOUS PORT >OR =5 YEARS OF AGE	No
36581	REPLACE TUNNEL CV CATHETER	No
36589	REMOVAL CATHETER BROVIAC	No
36589	REMOVAL CATHETER BROVIAC PEDIATRIC	No
36589	REMOVAL CATHETER HEMODIALYSIS	No
36589	REMOVAL CATHETER HICKMAN	No
36589	REMOVAL CATHETER PERMCATH	No
36589	REMOVAL CATHETER PORT-A-CATH	No
36589	REMOVAL CATHETER VENOUS ACCESS ADULT	No
36589	REMOVAL CATHETER VENOUS ACCESS PEDIATRIC	No
36589	REMOVE TUNNELED CV CATHETER	No
36589	REMOVE TUNNELED CV CATHETER	No
36589	REMOVE TUNNELED CV CATHETER PEDIATRIC	No
36590	REMOVE MEDIPORT	No
36590	REMOVE MEDIPORT	No
36590	REMOVE TUNNELED CV CATHETER W/ SQ PORT	No
36819	CREATION ARTERIOVENOUS ANASTOMOSIS BY UPPER ARM CEPHALIC VEIN TRANSPOSITION OPEN	No
36821	CREATION ARTERIOVENOUS ANASTOMOSIS DIRECT ANY SITE OPEN	No
36821	CREATION ARTERIOVENOUS ANASTOMOSIS DIRECT LOWER EXTREMITY OPEN	No

MSBOS - All records

Individual patient parameters (i.e., anemia, coagulopathy, transfusion risk) should be taken into consideration and may warrant an individual TSCR when not generally recommended.

June 22, 2023 **Sorted by CPT**

Primary CPT	Procedure Name	TSCR (Type and Screen) Needed?
36821	CREATION ARTERIOVENOUS ANASTOMOSIS DIRECT UPPER EXTREMITY OPEN	No
36821	CREATION ARTERIOVENOUS ANASTOMOSIS DIRECT UPPER EXTREMITY OPEN	No
36830	CREATE ARTERIOVENOUS GRAFT W/ NON-AUTOGENOUS GRAFT	No
36830	CREATE AV FISTULA VIA OTHER THAN DIRECT AV ANASTOMOSIS W/ NON-AUTOGENOUS GRAFT	No
36830	CREATION AV FISTULA W/ NONAUTOGENOUS GRAFT LOWER EXTREMITY	No
36830	CREATION OF ARTERIOVENOUS FISTULA; NONAUTOGENOUS GRAFT; UPPER EXTREMITY	No
36830	CREATION OF ARTERIOVENOUS FISTULA; NONAUTOGENOUS GRAFT; UPPER EXTREMITY	No
36830	IMPLANT GRAFT ARTERIOVENOUS	No
36832	REVISION FISTULA ARTERIOVENOUS EXTREMITY LOWER	No
36832	REVISION FISTULA ARTERIOVENOUS EXTREMITY UPPER	No
36832	REVISION FISTULA ARTERIOVENOUS EXTREMITY UPPER	No
36832	REVISION GRAFT ARTERIOVENOUS EXTREMITY LOWER	No
36832	REVISION GRAFT ARTERIOVENOUS EXTREMITY UPPER	No
36832	SUPERFICIALIZATION OF AUTOGENOUS DIALYSIS GRAFT UPPER EXTREMITY	No
36901	INTRO NEEDLE/CATH FOR ANGIO ARTERIOVENOUS SHUNT,FLUORO GUIDED,INCLUSICE OF RAD S&I	No
36901	INTRO NEEDLE/CATH FOR ANGIO ARTERIOVENOUS SHUNT,FLUORO GUIDED,INCLUSICE OF RAD S&I	No
36901	INTRO NEEDLE/CATH FOR ANGIO DIALYSIS CIRCUIT UPPER EXTREMITY,FLUORO GUIDED,INCLUSIVE OF RAD S&I	No
36901	INTRO NEEDLE/CATH FOR ANGIO DIALYSIS CIRCUIT,FLUORO GUIDED,INCLUSIVE OF RAD S&I	No
36902	AV SHUNTOGRAM AND ANGIOPLASTY OF PERIPHERAL DIALYSIS SEGMENT	No
36902	INTRO NEEDLE/CATH FOR PTA AV FISTULA LOWER EXTREMITY VENOUS SIDE W/ANGIO,FLUORO GUIDED,INCLUSIVE OF RAD S&I	No
36902	INTRO NEEDLE/CATH FOR PTA AV FISTULA UPPER EXTREMITY VENOUS SIDE W/ANGIO,FLUORO GUIDED,INCLUSIVE OF RAD S&I	No
36902	INTRO NEEDLE/CATH FOR PTA AV FISTULA UPPER EXTREMITY VENOUS SIDE W/ANGIO,FLUORO GUIDED,INCLUSIVE OF RAD S&I	No
37191	INSERT INTRAVASCULAR VENA CAVA FILTER ENDOVASCULAR APPROACH W/ VASCULAR ACCESS VESSEL SELECTION & RADIOLOGICAL SUPERVISION/INTERPRETATION	No
37193	RETRIEVAL INTRAVASCULAR VENA CAVA FILTER ENDOVASCULAR APPROACH W/ VASCULAR ACCESS VESSEL SELECTION/RADIOLOGICAL SUPERVISION/INTERPRETATION INTRAPROCEDURAL ROADMAPPING/IMAGING GUIDANCE	No
37215	OPEN TRANSCATH PLACEMENT INTRAVASCULAR STENT(S) CERV CAROTID ARTERY INCLUDING ANGIOPLASTY WHEN PERFORMED AND RAD S&I W/DISTAL EMBOLIC PROTECTION	No
37215	PERC TRANSCATH PLACEMENT INTRAVASCULAR STENT(S) CERV CAROTID ARTERY INCLUDING ANGIOPLASTY WHEN PERFORMED AND RAD S&I W/DISTAL EMBOLIC PROTECTION	No
37215	PERC TRANSCATH PLACEMENT INTRAVASCULAR STENT(S) CERV CAROTID ARTERY INCLUDING ANGIOPLASTY WHEN PERFORMED AND RAD S&I W/DISTAL EMBOLIC PROTECTION	No
37221	ENDOVASCULAR REVASCULARIZE	Yes
37221	ENDOVASCULAR REVASCULARIZE OPEN ILIAC ARTERY UNILAT W/ TRANSLUMINAL STENT AND ANGIOPLASTY	Yes
37221	ENDOVASCULAR REVASCULARIZE OPEN ILIAC ARTERY UNILAT W/ TRANSLUMINAL STENT AND ANGIOPLASTY WITH IVL	Yes
37221	ENDOVASCULAR REVASCULARIZE PERCUTANEOUS ILIAC ARTERY UNILAT W/TRANSLUMINAL STENT & ANGIOPLASTY	Yes
37221	ENDOVASCULAR REVASCULARIZE PERCUTANEOUS ILIAC ARTERY UNILAT W/TRANSLUMINAL STENT & ANGIOPLASTY WITH IVL	Yes

MSBOS - All records

Individual patient parameters (i.e., anemia, coagulopathy, transfusion risk) should be taken into consideration and may warrant an individual TSCR when not generally recommended.

June 22, 2023 **Sorted by CPT**

Primary CPT	Procedure Name	TSCR (Type and Screen) Needed?
37224	ANGIOPLASTY ONLY,FEMORAL/POPLITEAL ARTERY,UNILATERAL	No
37224	OPEN TRANSLUMINAL ANGIOPLASTY FEMORAL/POPLITEAL ARTERY UNILATERAL	No
37224	OPEN TRANSLUMINAL ANGIOPLASTY FEMORAL/POPLITEAL ARTERY(S) UNILATERAL	No
37224	OPEN TRANSLUMINAL ANGIOPLASTY FEMORAL/POPLITEAL ARTERY(S) UNILATERAL	No
37224	PERCUTANEOUS TRANSLUMINAL ANGIOPLASTY FEMORAL ARTERY UNILATERAL	No
37224	PERCUTANEOUS TRANSLUMINAL ANGIOPLASTY FEMORAL/POPLITEAL ARTERY(S) UNILATERAL	No
37224	PERCUTANEOUS TRANSLUMINAL ANGIOPLASTY POPLITEAL ARTERY UNILATERAL	No
37226	REVASC ENDOVASC OPEN FEM/POP ART W/TRANS STENT PLACT W/ANGIO/W/IN THE SAME VESSEL WHEN PERFORMED	No
37226	REVASC ENDOVASC OPEN FEM/POP ART W/TRANS STENT PLACT W/ANGIO/W/IN THE SAME VESSEL WHEN PERFORMED	No
37226	REVASC ENDOVASC PERCUT FEM/POP ART W/TRANS STENT PLACT W/ANGIO/W/IN THE SAME VESSEL WHEN PERFORM	No
37228	OPEN TRANSLUMINAL ANGIOPLASTY TIBIAL/PERONEAL ARTERY UNILATERAL INITIAL VESSEL	No
37228	OPEN TRANSLUMINAL ANGIOPLASTY TIBIAL/PERONEAL ARTERY UNILATERAL INITIAL VESSEL	No
37228	OPEN TRANSLUMINAL ANGIOPLASTY TIBIAL/PERONEAL ARTERY UNILATERAL INITIAL VESSEL WITH IVL	No
37228	PERCUTANEOUS TRANSLUMINAL ANGIOPLASTY TIBIAL/PERONEAL ARTERY UNILATERAL INITIAL VESSEL	No
37228	PERCUTANEOUS TRANSLUMINAL ANGIOPLASTY TIBIAL/PERONEAL ARTERY UNILATERAL INITIAL VESSEL WITH IVL	No
37236	OPEN TRANSCATH PLACEMENT INTRAVASCULAR STENT(S) INITIAL ARTERY (EXCEPT LOWER EXTREM,CERV CAROTID,EXTRACRANIAL VERT, INTRATHOR CAROTID,INTRACRANIAL,CORONARY) INCLUDING RAD S&I	No
37236	OPEN TRANSCATH PLACEMENT INTRAVASCULAR STENT(S) INITIAL ARTERY (EXCEPT LOWER EXTREM,CERV CAROTID,EXTRACRANIAL VERT, INTRATHOR CAROTID,INTRACRANIAL,CORONARY) INCLUDING RAD S&I	No
37236	PERCUTAEOUS TRANSCATH PLACEMENT INTRAVASCULAR STENT(S) INCLUDING RAD S&I, AORTA	No
37236	PERCUTAEOUS TRANSCATH PLACEMENT INTRAVASCULAR STENT(S) INCLUDING RAD S&I, BRACHIAL ARTERY	No
37236	PERCUTANEOUS TRANSCATH PLACEMENT INTRAVASCULAR STENT(S),INCLUDING RAD S&I SUBCLAVIAN ARTERY	No
37236	PERCUTANEOUS TRANSCATH PLACEMENT INTRAVASCULAR STENT(S) INITIAL ARTERY (EXCEPT LOWER EXTREM,CERV CAROTID,EXTRACRANIAL VERT, INTRATHOR CAROTID,INTRACRANIAL,CORONARY),INCLUDING RAD S&I	No
37242	VASCULAR EMBOLIZATION OR OCCLUSION, INCLUSIVE OF ALL RADIOLOGICAL S&I; ARTERIAL, OTHER THAN HEMORRHAGE	No
37607	LIGATION FISTULA ARTERIOVENOUS EXTREMITY LOWER	No
37607	LIGATION FISTULA ARTERIOVENOUS EXTREMITY UPPER	No
37607	LIGATION FISTULA ARTERIOVENOUS EXTREMITY UPPER	No
37607	LIGATION OR BANDING OF ANGIOACCESS ARTERIOVENOUS FISTULA	No
37609	BIOPSY ARTERY TEMPORAL	No
37785	LIGATION / EXCISION VARICOSE VEIN CLUSTER 1 LEG	No
37799	ANEURYSMORRHAPHY POST ENDOVASCULAR ABDOMINAL AORTIC ANEURYSM	No
37799	ARTERY EXPOSURE	No
37799	ATHRECTOMY OF AV FISTUAL	No
37799	BYPASS GRAFT W/OTHER THAN VEIN AXILLARY-BRACHIAL	No
37799	BYPASS GRAFT WITH VEIN COMMON ILIAC CONTRALATERAL COMMON ILIAC	No
37799	CREATION ARTERIOVENOUS ANASTOMOSIS BY SUPERFICIAL FEMORAL VEIN TRANSPOSITION OPEN	No

MSBOS - All records

Individual patient parameters (i.e., anemia, coagulopathy, transfusion risk) should be taken into consideration and may warrant an individual TSCR when not generally recommended.

June 22, 2023 **Sorted by CPT**

Primary CPT	Procedure Name	TSCR (Type and Screen) Needed?
37799	ENDOVASC RPR ASCENDING THORACIC AORTA W/ INITIAL ENDOPROSTHESIS RADIOLOGICAL SUPERVISE/INTERPRET	No
37799	EVACUATION HEMATOMA FISTULA PERIVASCULAR	No
37799	EXCISION OF THROMBOPHLEBITIC VEIN(S)	No
37799	EXCISION THROMBOSED VEIN, ARM	No
37799	EXCISION/LIGATION ABDOMINAL VEINS	No
37799	LAPAROSCOPIC LIGATION PORTAL VEIN	No
37799	OPEN DIRECT REPAIR OF PORTAL BLOOD VESSEL W THROMBECTOMY	No
37799	OPEN EVACUATION OF SUBCLAVIAN ARTERY	No
37799	OPEN RELEASE OF MEDIAN ARCUATE LIGAMENT	No
37799	OPEN RELEASE OF MEDIAN ARCUATE LIGAMENT	No
37799	REMOVAL CATHETER DIALYSIS	No
37799	REMOVAL OF MALFUNCTIONING GRAFT	No
37799	REMOVE AV FISTULA	No
37799	RESECT VENOUS ANEURYSM UPPER EXTREMITY	No
37799	RESECTION FALSE ANEURYSM UPPER EXTREMITY	No
37799	RESECTION INFERIOR VENA CAVA, KIDNEY	No
37799	RESECTION VARICOSE VEIN UNSPECIFIED	No
37799	REVISION BYPASS GRAFT, W/ OTHER THAN VEIN: FEMORAL	No
37799	SCLEROTHERAPY OF VENOUS MALFORMATION	No
37799	SHUNT; CORONARY-CAVAL	No
37799	STAB PHLEBECTOMY VARICOSE VEINS 1 EXTREMITY; <10 INCISIONS	No
37799	TRANSPOSITION OF BRACHIAL ARTERY TO THE PROFUNDA BRACHII ARTERY	No
38120	LAPAROSCOPIC SPLENECTOMY	Yes
38120	LAPAROSCOPIC SPLENECTOMY	Yes
38120	ROBOTIC LAPAROSCOPIC SPLENECTOMY	Yes
38500	BIOPSY NODE SENTINEL NECK	No
38500	BIOPSY OR EXCISION LYMPH NODE(S); OPEN, SUPERFICIAL	No
38500	BIOPSY OR EXCISION LYMPH NODE(S); OPEN, SUPERFICIAL	No
38510	BIOPSY NODE CERVICAL NECK	No
38510	BIOPSY OR EXCISION LYMPH NODE(S) OPEN, DEEP CERVICAL	No
38510	BIOPSY OR EXCISION LYMPH NODE(S) OPEN, DEEP CERVICAL	No
38510	BIOPSY OR EXCISION LYMPH NODE(S) OPEN, DEEP CERVICAL PEDIATRIC	No
38510	EXCISION NODE CERVICAL	No
38525	BIOPSY AXILLARY NODE OPEN DEEP	No
38525	BIOPSY NODE AXILLARY	No
38525	BIOPSY NODE SENTINEL AXILLARY	No
38525	EXCISION LYMPH NODE OPEN DEEP	No

MSBOS - All records

Individual patient parameters (i.e., anemia, coagulopathy, transfusion risk) should be taken into consideration and may warrant an individual TSCR when not generally recommended.
June 22, 2023 **Sorted by CPT**

Primary CPT	Procedure Name	TSCR (Type and Screen) Needed?
38525	EXCISION LYMPH NODE OPEN DEEP AXILLARY NODE	No
38525	EXCISION NODE AXILLARY	No
38531	BIOPSY OR EXCISION INGUINALFEMORAL LYMPH NODE PEDIATRIC,OPEN	No
38531	OPEN BIOPSY/EXCISION INGUINOFEMORAL NODES	No
38531	OPEN BIOPSY/EXCISION INGUINOFEMORAL NODES	No
38724	CERVICAL LYMPHADENECTOMY	No
38724	DISSECTION NECK MODIFIED RADICAL	No
38724	DISSECTION NECK MODIFIED RADICAL	No
38724	DISSECTION NECK RADICAL	No
38724	DISSECTION NECK RADICAL PEDIATRIC	No
38900	INTRAOPERATIVE ID OF SENTINEL LYMPH NODE(S) INCL'D INJECTION OF NON-RAD DYE WHEN PERFORMED	No
38999	ASPIRATION BONE MARROW W/ BX.	No
38999	CONTROL OF LYMPHATIC LEAK	No
38999	EXCISION GENITAL LYMPHADEMA < 10CM DIAMETER	No
38999	EXCISION GENITAL LYMPHADEMA < 10CM DIAMETER	No
38999	IMMEDIATE LYMPHOVENOUS BYPASS/ANASTOMOSIS (LVA)	No
38999	LYMPHOVENOUS BYPASS/ ANASTOMOSIS	No
42415	EXCISION PAROTID GLAND LATERAL LOBE W/ DISSECTION & NERVE PRES	No
42415	EXCISION PAROTID GLAND LATERAL LOBE W/ DISSECTION & NERVE PRES PEDIATRIC	No
42415	EXCISION PAROTID TUMOR LATERAL LOBE W/ DISSECTION & NERVE PRES	No
42415	PAROTIDECTOMY	No
42415	PAROTIDECTOMY	No
42415	PAROTIDECTOMY PEDIATRIC	No
42699	ENDOSCOPIC REMOVAL STONES SALIVARY DUCTS	No
42699	ENDOSCOPY PAROTID/SALIVARY DUCT	No
42699	ENDOSCOPY PAROTID/SALIVARY DUCT	No
42699	SIALOLITHOTOMY SUBMANDIBULAR INTRAORAL CO2 LASER	No
42820	TONSILLECTOMY AND ADENOIDECTOMY, YOUNGER THAN AGE 12	No
42820	TONSILLECTOMY AND ADENOIDECTOMY, YOUNGER THAN AGE 12	No
42820	TONSILLECTOMY AND ADENOIDECTOMY, YOUNGER THAN AGE 12 PEDIATRIC	No
42821	COBLATION TONSILLECTOMY AGE 12 OR OVER	No
42821	TONSILLECTOMY AND ADENOIDECTOMY, AGE 12 OR OVER	No
42821	TONSILLECTOMY AND ADENOIDECTOMY, AGE 12 OR OVER	No
42826	TONSILLECTOMY ADULT	No
42826	TONSILLECTOMY ADULT	No
42826	TONSILLECTOMY VIA CAUTERIZATION AGE 12 OR OVER	No
42826	TONSILLECTOMY, AGE 12 OR OVER	No

MSBOS - All records

Individual patient parameters (i.e., anemia, coagulopathy, transfusion risk) should be taken into consideration and may warrant an individual TSCR when not generally recommended.

June 22, 2023 **Sorted by CPT**

Primary CPT	Procedure Name	TSCR (Type and Screen) Needed?
42830	ADENOIDECTOMY PRIMARY YOUNGER THAN AGE 12	No
42962	SURGICAL INTERVENTION TO CONTROL ORORPHARYNGEAL HEMORRHAGE	No
43117	ESOPHAGECTOMY DISTAL WITH THORACIC ESOPHAGOGASTROSTOMY VIA THORACOTOMY AND ABDOMEN	Yes
43246	ENDOSCOPIC PERCUTANEOUS INSERTION TUBE GASTROSTOMY	No
43279	LAPAROSCOPIC ESOPHAGOMYOTOMY W/ FUNDOPLASTY HELLER TYPE	No
43279	LAPAROSCOPIC ESOPHAGOMYOTOMY W/ FUNDOPLASTY HELLER TYPE	No
43279	LAPAROSCOPIC ESOPHAGOMYOTOMY W/O FUNDOPLASTY HELLER TYPE	No
43279	ROBOTIC ESOPHAGOMYOTOMY W/ FUNDOPLASTY HELLER TYPE	No
43280	LAPAROSCOPIC ESOPHAGOGASTRIC FUNDOPLASTY PEDIATRIC	No
43280	LAPAROSCOPIC FUNDOPLICATION NISSEN	No
43280	LAPAROSCOPIC FUNDOPLICATION NISSEN	No
43280	LAPAROSCOPIC FUNDOPLICATION PARTIAL	No
43280	ROBOTIC LAPAROSCOPIC FUNDOPLICATION NISSEN	No
43281	LAPAROSCOPIC RPR PARAESOHAGEAL HERNIA W/ FUNDOPLASTY W/O MESH	No
43281	LAPAROSCOPIC RPR PARAESOHAGEAL HERNIA W/ FUNDOPLASTY W/O MESH	No
43281	LAPAROSCOPIC RPR PARAESOPHAGEAL HERNIA W/O FUNDOPLASTY W/O MESH	No
43281	ROBOTIC LAPAROSCOPIC REPAIR PARAESOPHAGEAL HERNIA W/FUNDOPLASTY W/O IMPLANTATION OF MESH	No
43281	SI ROBOTIC LAPAROSCOPIC REPAIR PARAESOPHAGEAL HERNIA W/FUNDOPLASTY W/O IMPLANTATION OF MESH	No
43281	XI ROBOTIC LAPAROSCOPIC REPAIR PARAESOPHAGEAL HERNIA W/FUNDOPLASTY W/O IMPLANTATION OF MESH	No
43282	LAPAROSCOPIC RPR PARAESOHAGEAL HERNIA W/ FUNDOPLASTY W/ MESH	No
43282	LAPAROSCOPIC RPR PARAESOHAGEAL HERNIA W/ FUNDOPLASTY W/ MESH	No
43282	LAPAROSCOPIC RPR PARAESOPHAGEAL HERNIA W/MESH	No
43282	LAPAROSCOPIC RPR PARAESOPHAGEAL HERNIA W/O FUNDOPLASTY W/ MESH	No
43282	ROBOTIC LAPAROSCOPIC RPR PARAESOPHAGEAL HERNIA W/MESH	No
43282	ROBOTIC LAPAROSCOPIC RPR PARAESOPHAGEAL HERNIA W/O FUNDOPLASTY W/ MESH	No
43497	TRANSORAL LOWER ESOPHAGEAL MYOTOMY	No
43644	LAPAROSCOPIC GASTRIC RESTRICTIVE SURG W/ BYPASS & ROUX-EN-Y 150CM OR LESS	No
43644	LAPAROSCOPIC GASTRIC RESTRICTIVE SURG W/ BYPASS & ROUX-EN-Y 150CM OR LESS	No
43644	ROBOTIC LAPAROSCOPIC GASTRIC RESTRICTIVE SURG W/ BYPASS & ROUX-EN-Y </= 150CM	No
43644	XI ROBOTIC LAPAROSCOPIC GASTRIC RESTRICTIVE SURG W/ BYPASS & ROUX-EN-Y </= 150CM	No
43653	LAPAROSCOPIC GASTROSTOMY W/O CONSTRUCTION OF GASTRIC TUBE ADULT	No
43653	LAPAROSCOPIC PERCUTANEOUS GASTROSTOMY	No
43653	LAPAROSCOPIC PERCUTANEOUS GASTROSTOMY	No
43653	LAPAROSCOPIC PERCUTANEOUS INSERTION TUBE GASTROSTOMY	No
43659	LAPAROSCOPIC ANTECOLIC ANTEGASTRIC GASTRECTOMY,PARTIAL,WITH ROUX-EN-Y RECONSTRUCTION	No
43659	LAPAROSCOPIC ASSISTED ENDOSCOPIC RESECTION OF DUODENAL MASS	No
43659	LAPAROSCOPIC CURVATURE PLICATION	No

MSBOS - All records

Individual patient parameters (i.e., anemia, coagulopathy, transfusion risk) should be taken into consideration and may warrant an individual TSCR when not generally recommended.

June 22, 2023 **Sorted by CPT**

Primary CPT	Procedure Name	TSCR (Type and Screen) Needed?
43659	LAPAROSCOPIC EXCISION OF BENIGN GIST TUMOR	No
43659	LAPAROSCOPIC GASTRECTOMY	No
43659	LAPAROSCOPIC GASTRECTOMY GASTRIC RESTRICTIVE PARTIAL (50 TO 100 CM COMMON CHANNEL) TO LIMIT ABSORPTION	No
43659	LAPAROSCOPIC GASTRECTOMY TOTAL W/ ROUX-EN-Y RECONSTRUCTION	No
43659	LAPAROSCOPIC GASTRECTOMY, PARTIAL; WITH GASTROJEJUNOSTOMY	No
43659	LAPAROSCOPIC GASTRIC ULCER REPAIR - VISCERAL PERFORATION	No
43659	LAPAROSCOPIC GASTROJEJUNOSTOMY W/O VAGOTOMY	No
43659	LAPAROSCOPIC GASTROPLASTY	No
43659	LAPAROSCOPIC GASTROTOMY W/ EXPLORATION OR FOREIGN BODY REMOVAL	No
43659	LAPAROSCOPIC PYLOROMYOTOMY PEDIATRIC	No
43659	LAPAROSCOPIC PYLOROPLASTY	No
43659	LAPAROSCOPIC REPAIR PERFORATED ULCER	No
43659	LAPAROSCOPIC REVISE GASTRIC RESTRICTIVE PROCEDURE OTHER THAN ADJUSTABLE GASTRIC RESTRICT DEVICE	No
43659	LAPAROSCOPIC REVISION OF GASTROJEJUNAL ANASTOMOSIS (GASTROJEJUNOSTOMY) WITH RECONSTRUCTION,WITH OR WITHOUT PARTIAL GASTRECTOMY OR INTESTINE RESECTION; WITHOUT VAGOTOMY	No
43659	LAPAROSCOPIC REVISION OF GASTROJEJUNAL ANASTOMOSIS (GASTROJEJUNOSTOMY) WITH RECONSTRUCTION,WITH OR WITHOUT PARTIAL GASTRECTOMY OR INTESTINE RESECTION; WITHOUT VAGOTOMY	No
43659	LAPAROSCOPIC WEDGE RESECTION OF MALIGNANT TUMOR OF THE STOMACH	No
43659	LAPAROSCOPY EXCISION MALIGNANT TUMOR OF STOMACH	No
43659	LAPAROSCOPY SURGICAL GASTRIC RESTRICTIVE PROCEDURE BILIOPANCREATIC DIVERSION WITH DUODENAL SWITCH	No
43659	LAPAROSCOPY SURGICAL W/GASTROPEXY	No
43659	ROBOTIC LAPAROSCOPIC CLOSURE GASTRIC FISTULA CUTANEOUS	No
43659	ROBOTIC LAPAROSCOPIC GASTRECTOMY PARTIAL W/GASTROJEJUNOSTOMY	No
43659	XI ROBOTIC LAPAROSCOPIC GASTRECTOMY PARTIAL W/GASTROJEJUNOSTOMY	No
43775	LAPAROSCOPIC LONGITUDINAL GASTRECTOMY, GASTRIC RESTRICTIVE PROCEDURE	No
43775	LAPAROSCOPIC LONGITUDINAL GASTRECTOMY, GASTRIC RESTRICTIVE PROCEDURE	No
43775	ROBOTIC LONGITUDINAL GASTRECTOMY,GASTRIC RESTRICTIVE PROCEDURE	No
43775	XI ROBOTIC LONGITUDINAL GASTRECTOMY,GASTRIC RESTRICTIVE PROCEDURE	No
43840	GASTRORRHAPHY, SUTURE PERFORATED DUODENAL OR GASTRIC ULCER, WOUND, OR INJURY	Yes
43840	GASTRORRHAPHY, SUTURE PERFORATED DUODENAL OR GASTRIC ULCER, WOUND, OR INJURY	Yes
43840	GASTRORRHAPHY, SUTURE PERFORATED DUODENAL ULCER	Yes
43999	ENDOSCOPIC CLOSURE OF GASTRIC FISTULA	No
43999	ENDOSCOPIC PER-ORAL PYLOROMYOTOMY	No
43999	ENDOSCOPIC PER-ORAL PYLOROMYOTOMY	No
43999	GASTRECTOMY PARTIAL PROXIMAL	No
43999	GASTRIC REPAIR, WITH OR WITHOUT OMENTAL PATCH	No
43999	REVISION OF NISSEN FUNDOPLASTY	No

MSBOS - All records

Individual patient parameters (i.e., anemia, coagulopathy, transfusion risk) should be taken into consideration and may warrant an individual TSCR when not generally recommended.
June 22, 2023 **Sorted by CPT**

Primary CPT	Procedure Name	TSCR (Type and Screen) Needed?
44005	ENTEROLYSIS	No
44005	ENTEROLYSIS ADULT	No
44005	ENTEROLYSIS PEDIATRIC	No
44120	EXCISION CYST / MASS BOWEL SMALL	Yes
44120	RESECTION BOWEL SMALL	Yes
44120	RESECTION BOWEL SMALL	Yes
44125	ENTERECTOMY, RESECTION SMALL INTESTINE W/ ENTEROSTOMY	Yes
44130	ENTEROENTEROSTOMY, ANASTOMOSIS OF INTESTINE	Yes
44140	COLECTOMY - LITHOTOMY	Yes
44140	COLECTOMY - SUPINE	Yes
44140	COLECTOMY EXTENDED RIGHT W/ ILEOCOLIC ANASTAMOSIS (ICA)	Yes
44140	COLECTOMY HEMI-RIGHT W/ ILEOCOLIC ANASTAMOSIS (ICA)	Yes
44140	COLECTOMY SIGMOID	Yes
44140	COLECTOMY SIGMOID W/ COLORECTAL ANASTOMOSIS	Yes
44140	COLECTOMY TRANSVERSE	Yes
44140	HEMICOLECTOMY PARTIAL WITH ANASTOMOSIS	Yes
44140	HEMICOLECTOMY PARTIAL WITH ANASTOMOSIS	Yes
44140	RESECTION COLON ADULT	Yes
44140	RESECTION COLON SIGMOID	Yes
44143	HARTMANN PROCEDURE	Yes
44143	HEMICOLECTOMY PARTIAL WITH END COLOSTOMY AND CLOSURE DISTAL SEGMENT	Yes
44143	HEMICOLECTOMY PARTIAL WITH END COLOSTOMY AND CLOSURE DISTAL SEGMENT	Yes
44145	COLECTOMY, ANASTOMOSIS ANTERIOR LOW	Yes
44145	HEMICOLECTOMY PARTIAL WITH COLOPROCTOSTOMY	Yes
44145	HEMICOLECTOMY PARTIAL WITH COLOPROCTOSTOMY	Yes
44145	RESECTION COLON LOW ANTERIOR	Yes
44150	COLECTOMY ABDOMINAL W/O PROCTECTOMY W/ ILEOSTOMY/ILEOPROCTOSTOMY, TOTAL	Yes
44150	COLECTOMY ABDOMINAL W/O PROCTECTOMY W/ ILEOSTOMY/ILEOPROCTOSTOMY, TOTAL	Yes
44150	COLECTOMY SUBTOTAL	Yes
44150	CREATION OR REVISION ILEORECTAL ANASTAMOSIS	Yes
44160	COLECTOMY PARTIAL	Yes
44160	COLECTOMY PARTIAL OPEN RIGHT WITH ILEOCOLOSTOMY	Yes
44160	COLECTOMY PARTIAL OPEN WITH ILEOCOLOSTOMY	Yes
44160	COLECTOMY PARTIAL W/REMOVAL TERMINAL ILEUM W/ILEOCOLOSTOMY	Yes
44160	ILEOCOLECTOMY	Yes
44160	LAPAROSCOPIC ILEOCECECTOMY	Yes
44180	LAPAROSCOPY DIAGNOSTIC WITH LYSIS ADHESIONS INTESTINAL	No

MSBOS - All records

Individual patient parameters (i.e., anemia, coagulopathy, transfusion risk) should be taken into consideration and may warrant an individual TSCR when not generally recommended.

June 22, 2023 **Sorted by CPT**

Primary CPT	Procedure Name	TSCR (Type and Screen) Needed?
44180	LAPAROSCOPY ENTEROLYSIS	No
44180	LAPAROSCOPY ENTEROLYSIS	No
44180	LAPAROSCOPY ENTEROLYSIS ADHESION REMOVAL PELVIC	No
44180	LAPAROSCOPY ENTEROLYSIS PEDIATRIC	No
44186	LAPAROSCOPIC JEJUNOSTOMY	No
44187	LAPAROSCOPY SURGICAL ILEOSTOMY NON-TUBE	No
44187	LAPAROSCOPY SURGICAL ILEOSTOMY NON-TUBE	No
44187	LAPAROSCOPY SURGICAL JEJUNOSTOMY NON-TUBE	No
44187	SINGLE PORT LAPAROSCOPY SURGICAL ILEOSTOMY NON-TUBE	No
44188	LAPAROSCOPIC CECOSTOMY SKIN LEVEL	No
44188	LAPAROSCOPIC COLOSTOMY	No
44188	LAPAROSCOPIC COLOSTOMY	No
44188	ROBOTIC LAPAROSCOPIC COLOSTOMY CREATION	No
44188	SINGLE PORT LAPAROSCOPIC COLOSTOMY OR SKIN LEVEL CECOSTOMY	No
44202	LAPAROSCOPY ENTERECTOMY RESECTION SMALL INTESTINE, SINGLE RESECTION AND ANASTOMOSIS	No
44204	LAPAROSCOPIC COLECTOMY RESECTION OF SMALL INTESTINE PARTIAL W/ ANASTOMOSIS	No
44204	LAPAROSCOPIC COLECTOMY SIGMOID COLON W/ COLORECTAL ANASTOMOSIS	No
44204	LAPAROSCOPIC HEMICOLECTOMY WITH ANASTOMOSIS	No
44204	LAPAROSCOPIC HEMICOLECTOMY WITH ANASTOMOSIS	No
44204	LAPAROSCOPIC RIGHT HEMICOLECTOMY, W/ICA	No
44204	ROBOTIC LAPAROSCOPIC COLECTOMY PARTIAL WITH ANASTOMOSIS	No
44204	ROBOTIC LAPAROSCOPIC COLECTOMY SIGMOID COLON W/ COLORECTAL ANASTOMOSIS	No
44204	XI ROBOTIC LAPAROSCOPIC COLECTOMY PARTIAL WITH ANASTOMOSIS	No
44204	XI ROBOTIC LAPAROSCOPIC COLECTOMY SIGMOID COLON W/ COLORECTAL ANASTOMOSIS	No
44205	LAPAROSCOPY COLECTOMY, PARTIAL, W/ REMOVAL TERMINAL ILEUM W/ ILEOCOLOSTOMY	No
44205	LAPAROSCOPY COLECTOMY, PARTIAL, W/ REMOVAL TERMINAL ILEUM W/ ILEOCOLOSTOMY	No
44205	ROBOTIC LAPAROSCOPIC COLECTOMY PARTIAL W/ REMOVAL OF TERMINAL ILEUM W/ ILEOCOLOSTOMY	No
44205	XI ROBOTIC LAPAROSCOPIC COLECTOMY PARTIAL W/ REMOVAL OF TERMINAL ILEUM W/ ILEOCOLOSTOMY	No
44206	COLECTOMY PARTIAL W/ END COLOSTOMY AND CLOSURE DISTAL SEGMENT, LAPAROSCOPIC	No
44206	COLECTOMY PARTIAL W/ END COLOSTOMY AND CLOSURE DISTAL SEGMENT, LAPAROSCOPIC	No
44206	LAPAROSCOPIC REVERSAL HARTMANN'S PROCEDURE	No
44207	COLECTOMY PARTIAL W/ END COLOSTOMY AND CLOSURE DISTAL SEGMENT, LAPAROSCOPIC	No
44207	COLECTOMY PARTIAL W/ END COLOSTOMY AND CLOSURE DISTAL SEGMENT, LAPAROSCOPIC	No
44207	LAPAROSCOPIC HAND ASSISTED COLECTOMY SIGMOID	No
44207	LAPAROSCOPIC HEMICOLECTOMY ANASTOMOSIS, WITH COLOPROCTOSTOMY	No
44207	ROBOTIC LAPAROSCOPY SURGICAL COLECTOMY PARTIAL W/ ANASTOMOSIS W/ COLOPROCTOSTOMY	No
44207	SINGLE PORT LAPAROSCOPY SURGICAL COLECTOMY PARTIAL W/ ANASTOMOSIS W/ COLOPROCTOSTOMY	No

MSBOS - All records

Individual patient parameters (i.e., anemia, coagulopathy, transfusion risk) should be taken into consideration and may warrant an individual TSCR when not generally recommended.

June 22, 2023 **Sorted by CPT**

Primary CPT	Procedure Name	TSCR (Type and Screen) Needed?
44207	XI ROBOTIC LAPAROSCOPIC RESECTION COLON LOW ANTERIOR W/ COLORECTAL ANASTOMOSIS	No
44207	XI ROBOTIC LAPAROSCOPY SURGICAL COLECTOMY PARTIAL W/ ANASTOMOSIS W/ COLOPROCTOSTOMY	No
44210	LAPAROSCOPIC ASSISTED COLECTOMY	Yes
44210	LAPAROSCOPIC TOTAL COLECTOMY LITHOTOMY	Yes
44210	LAPAROSCOPIC TOTAL COLECTOMY W/ ILEOPROCTOSTOMY	Yes
44210	LAPAROSCOPIC TOTAL COLECTOMY W/ ILEOSTOMY	Yes
44210	LAPAROSCOPIC TOTAL COLECTOMY W/ ILEOSTOMY	Yes
44213	COLECTOMY PARTIAL, LAPAROSCOPIC	No
44213	COLECTOMY PARTIAL, LAPAROSCOPIC	No
44213	LAPAROSCOPIC TAKE-DOWN OF SPLENIC FLEXURE PERFORMED W/ PARTIAL COLECTOMY	No
44213	ROBOTIC LAPAROSCOPIC TAKE-DOWN OF SPLENIC FLEXURE PERFORMED W/ PARTIAL COLECTOMY	No
44213	XI ROBOTIC LAPAROSCOPIC TAKE-DOWN OF SPLENIC FLEXURE PERFORMED W/ PARTIAL COLECTOMY	No
44227	LAP CLOSURE OF ENTEROSTOMY W/ RESECTION & ANASTOMOSIS	No
44227	LAP CLOSURE OF ENTEROSTOMY W/ RESECTION & ANASTOMOSIS	No
44227	LAPAROSCOPIC RESECTION BOWEL	No
44227	LAPAROSCOPIC RESECTION COLON	No
44227	ROBOTIC LAPAROSCOPIC END COLOSTOMY TAKEDOWN W/ RESECTION & ANASTOMOSIS	No
44227	ROBOTIC LAPAROSCOPIC END ILEOSTOMY TAKEDOWN	No
44238	COMBINED CO2 COLONOSCOPY AND LAPAROSCOPY	No
44238	LAPAROSCOPIC ASSISTED COLOTOMY	No
44238	LAPAROSCOPIC CLOSURE OF ENTEROVESICAL FISTULA W/O INTESTINAL OR BLADDER RESECTION	No
44238	LAPAROSCOPIC CORRECTION MALROTATION BY LYSIS OF DUODENAL BANDS AND/OR REDUCTION MIDGUT VOLVULUS	No
44238	LAPAROSCOPIC ENTEROENTEROSTOMY ANASTOMOSIS OF INTESTINE	No
44238	LAPAROSCOPIC ENTEROTOMY EXCISION OF SINGLE INTESTINAL LESION	No
44238	LAPAROSCOPIC ENTEROTOMY FOR EXPLORATION OF SMALL BOWEL	No
44238	LAPAROSCOPIC ENTEROTOMY FOR EXPLORATION OF SMALL BOWEL & REMOVE FOREIGN BODY	No
44238	LAPAROSCOPIC ENTEROTOMY SMALL BOWEL EXPLORATION AND BIOPSY	No
44238	LAPAROSCOPIC EXCISION OF MECKEL'S DIVERTICULUM	No
44238	LAPAROSCOPIC FISTULA CLOSURE	No
44238	LAPAROSCOPIC HAND ASSISTED COLOSTOMY REVERSE	No
44238	LAPAROSCOPIC INTESTINAL PLICATION	No
44238	LAPAROSCOPIC PROCTOSIGMOIDECTOMY W/ COLOANAL ANASTOMOSIS W/ ILEOSTOMY CREATION	No
44238	LAPAROSCOPIC REDUCTION INTERNAL HERNIA	No
44238	LAPAROSCOPIC REPAIR INTESTINAL CUTANEOUS FISTULA	No
44238	LAPAROSCOPIC REPAIR VOLVULUS INTUSSUSCEPTION	No
44238	LAPAROSCOPIC ROUX-EN-Y DUODENOJEJUNOSTOMY W/O GASTRECTOMY	No
44238	LAPAROSCOPIC STRICTUROPLASTY W/ DILATION FOR OBSTRUCTION 1ST	No

MSBOS - All records

Individual patient parameters (i.e., anemia, coagulopathy, transfusion risk) should be taken into consideration and may warrant an individual TSCR when not generally recommended.
June 22, 2023 **Sorted by CPT**

Primary CPT	Procedure Name	TSCR (Type and Screen) Needed?
44238	LAPAROSCOPIC SUTURE OF SMALL INTESTINE FOR PERFORATED ULCER DIVERTICULUM WOUND INJURY OR RUPTURE SINGLE PERFORATION	No
44238	LAPAROSCOPIC TAKEDOWN OF FISTULA	No
44238	ROBOTIC LAPAROSCOPIC PROCTOSIGMOIDECTOMY ANTERIO RIGHT W/ LOW PELVIC ANASTAMOSIS W/OSTOMY	No
44238	ROBOTIC LAPAROSCOPIC PROCTOSIGMOIDECTOMY ANTERIO RIGHT W/ LOW PELVIC ANASTAMOSIS W/OSTOMY	No
44238	XI ROBOTIC LAPAROSCOPIC PROCTOSIGMOIDECTOMY ANTERIO RIGHT W/ LOW PELVIC ANASTAMOSIS W/OSTOMY	No
44310	ILEOSTOMY	Yes
44310	ILEOSTOMY	Yes
44310	ILEOSTOMY NON-TUBE	Yes
44312	REVISION ILEOSTOMY SIMPLE	No
44320	CECOSTOMY SKIN LEVEL	Yes
44320	COLOSTOMY ADULT	Yes
44320	COLOSTOMY ADULT	Yes
44320	COLOSTOMY LOOP	Yes
44320	COLOSTOMY SKIN LEVEL	Yes
44320	OPEN LOOP COLOSTOMY OF SIGMOID COLON	Yes
44346	REVISION COLOSTOMY W/ REPAIR PARACOLOSTOMY HERNIA	Yes
44385	ENDOSCOPY SMALL INTESTINE POUCH DX W/ BRUSHING	No
44385	ENDOSCOPY SMALL INTESTINE POUCH DX W/ BRUSHING	No
44385	ENDOSCOPY SMALL INTESTINE POUCH DX W/O SPEC COLLECTION	No
44386	ENDO EVAL OF SMALL INTESTINE POUCH W/ BIOPSY	No
44386	ENDO EVAL OF SMALL INTESTINE POUCH W/ BIOPSY	No
44386	ENDO EVAL SMALL INTESTINAL POUCH (EG, KOCK POUCH, ILEAL RESERVOIR	No
44602	EXPLORATORY LAPAROTOMY REPAIR PERFORATED VISCUS	Yes
44602	SUTURE OF SMALL INTESTINE FOR PERFORATED ULCER DIVERTICULUM WOUND INJURY OR RUPTURE SINGLE PERFORATION	Yes
44602	SUTURE OF SMALL INTESTINE FOR PERFORATED ULCER DIVERTICULUM WOUND INJURY OR RUPTURE SINGLE PERFORATION	Yes
44620	CLOSURE COLOSTOMY PEDIATRIC	No
44620	CLOSURE COLOSTOMY SUPINE ADULT	No
44620	CLOSURE ENTEROSTOMY LARGE INTESTINE	No
44620	CLOSURE ENTEROSTOMY SMALL INTESTINE	No
44620	CLOSURE ILEOSTOMY	No
44620	CLOSURE ILEOSTOMY	No
44620	CLOSURE ILEOSTOMY PEDIATRIC	No
44620	CLOSURE JEJUNOSTOMY	No
44620	TAKEDOWN COLOSTOMY	No
44625	CLOSURE ENTEROSTOMY, LARGE OR SMALL INTESTINE W/ RESECTION & ANASTOMOSIS OTHER THAN COLORECTAL	No
44626	CLOSURE COLOSTOMY LITHOTOMY ADULT	No

MSBOS - All records

Individual patient parameters (i.e., anemia, coagulopathy, transfusion risk) should be taken into consideration and may warrant an individual TSCR when not generally recommended.
June 22, 2023 **Sorted by CPT**

Primary CPT	Procedure Name	TSCR (Type and Screen) Needed?
44626	CLOSURE ENTEROSTOMY LARGE OR SMALL INTESTINE W/ RESECTION AND COLORECTAL ANASTOMOSIS	No
44626	CLOSURE ENTEROSTOMY LARGE OR SMALL INTESTINE W/ RESECTION AND COLORECTAL ANASTOMOSIS	No
44640	CLOSE INTESTINAL CUTANEOUS FISTULA	Yes
44799	APPENDICOSTOMY MALONE	Yes
44799	PANCREAS PRESERVING DUODENECTOMY	Yes
44799	POUCHOSCOPY WITH BALLOON DILATION	Yes
44799	POUCHOSCOPY WITH SNARE	Yes
44799	POUCHOSCOPY WITH SNARE	Yes
44799	REANASTOMOSIS SMALL INTESTINE	Yes
44799	SERIAL TRANSVERSE ENTEROPLASTY	Yes
44799	UNLISTED PROCEDURE,SMALL INTESTINE	Yes
44970	LAPAROSCOPIC APPENDECTOMY	No
44970	LAPAROSCOPIC APPENDECTOMY ADULT	No
44970	LAPAROSCOPIC APPENDECTOMY PEDIATRIC	No
44970	SINGLE PORT LAPAROSCOPIC APPENDECTOMY PEDIATRIC	No
45100	BIOPSY ANORECTAL WALL ANAL APPROACH	No
45100	BIOPSY ANORECTAL WALL ANAL APPROACH ADULT	No
45100	BIOPSY ANORECTAL WALL ANAL APPROACH PEDIATRIC	No
45110	PROCTECTOMY	Yes
45110	PROCTECTOMY	Yes
45110	PROCTECTOMY COMPLETE W/ ABDOMINOPERINEAL W/ COLOSTOMY	Yes
45110	PROCTECTOMY COMPLEX W/ ABDOMINOPERINEAL W/ COLOSTOMY	Yes
45136	EXCISION OF ILEOANAL RESEVOIR W/ ILEOSTOMY	Yes
45136	EXCISION OF ILEOANAL RESEVOIR W/ ILEOSTOMY	Yes
45136	REVISION PELVIC POUCH (IPAA) W/ ILEOSTOMY	Yes
45171	EXCISE RECTAL TUMOR VIA TRANSANAL APPROACH	No
45171	EXCISE RECTAL TUMOR VIA TRANSANAL APPROACH	No
45171	EXCISE RECTAL TUMOR VIA TRANSANAL APPROACH W/O MUSCULARIS PROPRIA	No
45330	SIGMOIDOSCOPY FLEXIBLE	No
45330	SIGMOIDOSCOPY FLEXIBLE	No
45330	SIGMOIDOSCOPY, FLEXIBLE; DIAGNOSTIC,INCLUDING COLLECTION OF SPECIMEN(S) BY BRUSHING OR WASHING	No
45331	SIGMOIDOSCOPY FLEXIBLE WITH BIOPSY	No
45378	COLONOSCOPY	No
45378	COLONOSCOPY	No
45378	COLONOSCOPY HIGH RISK SCREENING FLEXIBLE	No
45378	COLONOSCOPY LOW RISK SCREENING FLEXIBLE	No
45378	COLONOSCOPY,FLEXIBLE; DIAGNOSTIC,INCLUDING COLLECTION OF SPECIMEN(S) BY BRUSHING OR WASHING	No

MSBOS - All records

Individual patient parameters (i.e., anemia, coagulopathy, transfusion risk) should be taken into consideration and may warrant an individual TSCR when not generally recommended.
June 22, 2023 **Sorted by CPT**

Primary CPT	Procedure Name	TSCR (Type and Screen) Needed?
45380	COLONOSCOPY FLEXIBLE W/ BIOPSY	No
45380	COLONOSCOPY FLEXIBLE W/ BIOPSY PEDIATRIC	No
45380	COLONOSCOPY WITH BIOPSY	No
45380	COLONOSCOPY WITH BIOPSY	No
45385	COLONOSCOPY, FLEXIBLE W/REMOVAL TUMOR(S), POLYP(S), OR OTHER LESION(S) BY SNARE TECHNIQUE	No
45395	LAPAROSCOPIC PROCTECTOMY COMPLETE W/ ABDOMINOPERINEAL W/ COLOSTOMY	Yes
45395	LAPAROSCOPIC PROCTECTOMY COMPLETE W/ ABDOMINOPERINEAL W/ COLOSTOMY	Yes
45395	ROBOTIC LAPAROSCOPIC ABDOMINOPERINEAL RESECTION WITH COLOSTOMY	Yes
45395	ROBOTIC LAPAROSCOPIC PROCTECTOMY COMPLETE W/ ABDOMINOPERINEAL W/COLOSTOMY	Yes
45395	XI ROBOTIC LAPAROSCOPIC ABDOMINOPERINEAL RESECTION WITH COLOSTOMY	Yes
45395	XI ROBOTIC LAPAROSCOPIC PROCTECTOMY COMPLETE W/ ABDOMINOPERINEAL W/COLOSTOMY	Yes
45399	CHROMOENDOSCOPY	No
45399	COLONOSCOPY RIGID OR FLEXIBLE TRANSABDOMINAL VIA COLOTOMY SINGLE OR MULTIPLE	No
45399	COLONOSCOPY THROUGH COLOSTOMY	No
45399	ENDOSCOPIC SUBMUCOSAL DISSECTION,COLON	No
45399	ENDOSCOPIC SUBMUCOSAL DISSECTION,COLON	No
45400	PROCTOPEXY LAPAROSCOPIC	No
45400	ROBOTIC LAPAROSCOPY PROCTOPEXY	No
45400	ROBOTIC LAPAROSCOPY PROCTOPEXY	No
45400	XI ROBOTIC LAPAROSCOPY PROCTOPEXY	No
45990	EXAM UNDER ANESTHESIA RECTAL	No
45999	2ND STAGE TURNBULL CUTAIT	No
45999	ENDOSCOPIC SUBMUCOSAL DISSECTION,RECTUM	No
45999	ENDOSCOPIC SUBMUCOSAL DISSECTION,RECTUM	No
45999	EXCISION CYST / MASS PERIRECTAL	No
45999	INFLATION OF ARTIFICIAL BOWEL SPHINCTER DEVICE	No
45999	REPAIR RECTUM W/ SUTURE	No
45999	RESECTION NEO-RECTUM ANUS W/COLOSTOMY	No
45999	RESECTION OF EXCESSIVE MUCOSA RECTUM	No
45999	REVISION ILEOANAL RESERVOIR STOMA W/ILEOSTOMY	No
46020	PLACEMENT OF SETON	No
46040	INCISION AND DRAINAGE OF ISCHIORECTAL ABSCESS	No
46040	INCISION AND DRAINAGE OF ISCHIORECTAL AND PERIRECTAL ABSCESS	No
46040	INCISION AND DRAINAGE OF PERIRECTAL ABSCESS	No
46040	INCISION AND DRAINAGE OF PERIRECTAL ABSCESS	No
46050	I&D PERIANAL ABSCESS SUPERFICIAL	No
46080	SPHINCTEROTOMY ANAL	No

MSBOS - All records

Individual patient parameters (i.e., anemia, coagulopathy, transfusion risk) should be taken into consideration and may warrant an individual TSCR when not generally recommended.
June 22, 2023 **Sorted by CPT**

Primary CPT	Procedure Name	TSCR (Type and Screen) Needed?
46255	HEMORRHOIDECTOMY, INTERNAL AND EXTERNAL SINGLE COLUMN/GROUP	No
46260	HEMORRHOIDECTOMY EXTERNAL AND INTERNAL 2 OR MORE COLUMNS/GROUPS	No
46270	FISTULECTOMY ANAL SUBCUTANEOUS	No
46270	FISTULECTOMY ANAL SUBCUTANEOUS	No
46270	FISTULOTOMY ANAL SUBCUTANEOUS	No
46270	FISTULOTOMY ANAL SUBCUTANEOUS PEDIATRIC	No
46275	ANAL FISTULECTOMY INTERSPHINCTERIC	No
46275	ANAL FISTULECTOMY INTERSPHINCTERIC	No
46275	ANAL FISTULOTOMY INTERSPHINCTERIC	No
46275	LIGATION OF INTERSPHINCTERIC FISTULA TRACT	No
46280	ANAL FISTULECTOMY	No
46280	ANAL FISTULECTOMY EXTRASPHINCTERIC W/ SETON DRAIN	No
46280	ANAL FISTULECTOMY EXTRASPHINCTERIC W/O SETON	No
46280	ANAL FISTULECTOMY SUPRASPHINCTERIC W/ SETON DRAIN	No
46280	ANAL FISTULECTOMY SUPRASPHINCTERIC W/O SETON	No
46280	ANAL FISTULECTOMY TRANSSPHINCTERIC W/ SETON DRAIN	No
46280	ANAL FISTULECTOMY TRANSSPHINCTERIC W/O SETON	No
46280	ANAL FISTULOTOMY EXTRASPHINCTERIC W/ SETON DRAIN	No
46280	ANAL FISTULOTOMY EXTRASPHINCTERIC W/O SETON	No
46280	ANAL FISTULOTOMY SUPRASPHINCTERIC W/ SETON DRAIN	No
46280	ANAL FISTULOTOMY TRANSSPHINCTERIC W/ SETON DRAIN	No
46280	ANAL FISTULOTOMY TRANSSPHINCTERIC W/O SETON	No
46288	CLOSURE OF ANAL FISTULA	No
46288	CLOSURE OF ANAL FISTULA W/RECTAL ADVANCEMENT FLAP	No
46288	CLOSURE OF ANAL FISTULA WITH RECTAL ADVANCEMENT FLAP ADULT	No
46505	CHEMODENERVATION OF INTERNAL ANAL SPHINCTER	No
46922	DESTRUCTION HERPETIC VESICLE ANUS ADULT	No
46922	DESTRUCTION LESION(S) ANUS PEDIATRIC	No
46922	DESTRUCTION OF CONDYLOMA ANUS ADULT	No
46922	DESTRUCTION OF LESION(S) ANUS	No
46922	DESTRUCTION OF LESION(S) ANUS ADULT	No
46922	DESTRUCTION OF PAPILLOMA ANUS ADULT	No
46922	EXCISION CONDYLOMA RECTAL	No
46922	SURGICAL EXCISION LESION ANAL	No
46948	HEMORRHOIDECTOMY INTERNAL	No
46948	HEMORRHOIDECTOMY INTERNAL TRANSANAL HEMORRHOIDAL DEARTERIALIZATION, 2 OR MORE HEMORRHOID COLUMNS/GROUPS INCL ULTRASOUND GUIDANCE W/MUCOPEXY WHEN PERFORMED	No

MSBOS - All records

Individual patient parameters (i.e., anemia, coagulopathy, transfusion risk) should be taken into consideration and may warrant an individual TSCR when not generally recommended.
June 22, 2023 **Sorted by CPT**

Primary CPT	Procedure Name	TSCR (Type and Screen) Needed?
46948	INT HEMORRHOIDECTOMY TRANSANAL HROID DARTLZJ 2+ W/US GDN	No
47120	HEPATECTOMY RESECTION OF LIVER, PARTIAL LOBECTOMY	Yes
47120	HEPATECTOMY RESECTION OF LIVER, PARTIAL LOBECTOMY	Yes
47120	RESECTION LIVER PARTIAL	Yes
47135	TRANSPLANT LIVER ORTHOTOPIC	Yes
47135	TRANSPLANT LIVER ORTHOTOPIC PARTIAL	Yes
47135	TRANSPLANT LIVER ORTHOTOPIC PARTIAL PEDIATRIC	Yes
47135	TRANSPLANT LIVER ORTHOTOPIC WHOLE	Yes
47370	LAPAROSCOPY W/ RF ABLATION OF LIVER TUMOR	Yes
47379	LAPAROSCOPIC BIOPSY LIVER	No
47379	LAPAROSCOPIC BIOPSY LIVER	No
47379	LAPAROSCOPIC DONOR HEPATECTOMY,FROM LIVING DONOR; TOTAL LEFT LOBECTOMY	No
47379	LAPAROSCOPIC DONOR HEPATECTOMY,LIVING DONOR; TOTAL RIGHT LOBECTOMY	No
47379	LAPAROSCOPIC HEPATECTOMY RESECTION OF LIVER PARTIAL	No
47379	LAPAROSCOPIC REMOVAL OR UNROOFING CYST(S), HEPATIC	No
47379	LAPAROSCOPIC RESECTION LIVER	No
47379	ROBOTIC LAPAROSCOPIC HEPATECTOMY RESECTION OF LIVER, PARTIAL LOBECTOMY	No
47379	XI ROBOTIC LAPAROSCOPIC HEPATECTOMY RESECTION OF LIVER, PARTIAL LOBECTOMY	No
47562	LAPAROSCOPIC CHOLECYSTECTOMY	No
47562	LAPAROSCOPIC CHOLECYSTECTOMY	No
47562	LAPAROSCOPIC CHOLECYSTECTOMY PEDIATRIC	No
47562	LAPAROSCOPIC CHOLECYSTECTOMY POSSIBLE OPEN	No
47562	ROBOTIC LAPAROSCOPIC CHOLECYSTECTOMY	No
47562	ROBOTIC SINGLE PORT LAPAROSCOPIC CHOLECYSTECTOMY	No
47562	XI ROBOTIC LAPAROSCOPIC CHOLECYSTECTOMY	No
47563	LAPAROSCOPIC CHOLECYSTECTOMY WITH GRAMS	No
47563	LAPAROSCOPIC CHOLECYSTECTOMY WITH GRAMS	No
47563	ROBOTIC LAPAROSCOPIC CHOLECYSTECTOMY WITH GRAMS	No
47563	XI ROBOTIC LAPAROSCOPIC CHOLECYSTECTOMY WITH GRAMS	No
47600	CHOLECYSTECTOMY OPEN	Yes
47605	CHOLECYSTECTOMY WITH CHOLANGIOGRAMS	No
48140	PANCREATECTOMY PARTIAL WITHOUT PANCREATICOJEJUNOSTOMY	Yes
48150	WHIPPLE PROCEDURE, WITH PANCREATOJEJUNOST	Yes
48153	PANCREATECTOMY, SUBTOTAL W/DUODENECTOMY, CHOLEDOCHOENTEROSTOMY, DUODENOJEJUNOSTOMY, PANCREATOJEJUNOSTOMY	Yes
48999	ABLATION PANCREAS WITH IRREVERSIBLE ELECTROPORTATION	Yes
48999	EXPLORATION PANCREAS	Yes
48999	LAP WHIPPLE W/ NEAR TOTAL DUODENECTOMY W/O PANCREATICOJEJUNOSTOMY	Yes

MSBOS - All records

Individual patient parameters (i.e., anemia, coagulopathy, transfusion risk) should be taken into consideration and may warrant an individual TSCR when not generally recommended.
June 22, 2023 **Sorted by CPT**

Primary CPT	Procedure Name	TSCR (Type and Screen) Needed?
48999	LAP WHIPPLE W/ TOTAL DUODENECTOMY W/O PANCREATICOJEJUNOSTOMY	Yes
48999	LAPAROSCOPIC BIOPSY PANCREAS	Yes
48999	LAPAROSCOPIC DEBRIDEMENT OF NECROTIZING PANCREATITIS	Yes
48999	LAPAROSCOPIC DISTAL NEAR-TOTAL PANCREATECTOMY W/ PRESERVE DUODENUM	Yes
48999	LAPAROSCOPIC EXCISION LESION PANCREAS	Yes
48999	LAPAROSCOPIC PANCREATECTOMY W/O PANCREATICOJEJUNOSTOMY W/ SPLENECTOMY	Yes
48999	LAPAROSCOPIC PANCREATECTOMY W/O PANCREATICOJEJUNOSTOMY W/ SPLENECTOMY	Yes
48999	LAPAROSCOPIC RESECTION PANCREAS UNCINATE 1.4 CM MASS	Yes
48999	LAPAROSCOPIC SUBTOTAL PANCREATECTOMY W/ SPLENECTOMY	Yes
48999	LAPAROSCOPIC SUBTOTAL PANCREATECTOMY W/ SPLENECTOMY & JEJUNOSTOMY	Yes
48999	LAPAROSCOPIC SUBTOTAL PANCREATECTOMY W/O SPLENECTOMY	Yes
48999	LAPAROSCOPIC TOTAL PANCREATECTOMY	Yes
48999	OPEN CENTRAL PANCREATECTOMY	Yes
48999	PANCREAS STONE SHOCK WAVE LITHOTRIPSY	Yes
48999	ROBOTIC LAPAROSCOPIC WHIPPLE W/PANCREATOJEJUNOSTOMY	Yes
48999	XI ROBOTIC LAPAROSCOPIC WHIPPLE W/PANCREATOJEJUNOSTOMY	Yes
49000	DEBRIDEMENT ABDOMINAL WALL VIA LAPAROTOMY	Yes
49000	EVACUATION CLOT ABDOMINAL	Yes
49000	EXCISION ABDOMINAL WALL LESION VIA LAP	Yes
49000	EXPLORATOR CELIOTOMY W/O BIOPSY	Yes
49000	EXPLORATORY LAPAROTOMY	Yes
49000	EXPLORATORY LAPAROTOMY	Yes
49000	EXPLORATORY LAPAROTOMY ADULT	Yes
49000	EXPLORATORY LAPAROTOMY W/ BIOPSY	Yes
49000	EXPLORATORY LAPAROTOMY, EXPLORATORY CELIOTOMY W/ BIOPSY	Yes
49000	LAPAROTOMY MINI	Yes
49002	LAPAROTOMY SECOND LOOK	Yes
49002	LAPAROTOMY SECOND LOOK ADULT	Yes
49002	LAPAROTOMY SECOND LOOK PEDIATRIC	Yes
49002	LAPAROTOMY, REOPENING	Yes
49002	LAPAROTOMY, REOPENING	Yes
49002	LAPAROTOMY, REOPENING, PEDIATRIC	Yes
49203	EXCISION RETROPERITONEAL CYST / MASS < / = 5 CM DIAMETER [18171]	Yes
49203	EXCISION RETROPERITONEAL CYST / MASS < / = 5 CM DIAMETER [18171]	Yes
49203	EXCISION/DESTRUCTION OPEN ABDOMINAL TUMOR 5 CM/<	Yes
49205	EXCISION RETROPERITONEAL CYST / MASS > 10 CM DIAMETER [18174]	Yes
49320	INTRAOP LAPAROSCOPIC HERNIA EXAM ON OPPOSITE SIDE CHILD	Yes

MSBOS - All records

Individual patient parameters (i.e., anemia, coagulopathy, transfusion risk) should be taken into consideration and may warrant an individual TSCR when not generally recommended.
June 22, 2023 **Sorted by CPT**

Primary CPT	Procedure Name	TSCR (Type and Screen) Needed?
49320	LAPAROSCOPIC DIAGNOSTIC LOOK ABDOMEN / PERITONEUM / OMENTUM [18289]	Yes
49320	LAPAROSCOPY	Yes
49320	LAPAROSCOPY DIAGNOSTIC	Yes
49320	LAPAROSCOPY DIAGNOSTIC	Yes
49320	LAPAROSCOPY DIAGNOSTIC ABDOMEN, PERITONEUM AND OMENTUM	Yes
49320	LAPAROSCOPY DIAGNOSTIC PEDIATRIC	Yes
49320	PERITONEAL LAPAROSCOPY W/ SPECIMEN COLLECTION VIA BRUSHING	Yes
49320	ROBOTIC DIAGNOSTIC LAPAROSCOPY ABDOMEN, PERITONEUM AND OMENTUM	Yes
49320	SINGLE PORT LAPAROSCOPY, ABDOMEN, PERITONEUM, AND OMENTUM, DIAGNOSTIC WITHOUT COLLECTION OF SPECIMEN(S)	Yes
49320	SINGLE PORT LAPAROSCOPY, ABDOMEN, PERITONEUM, AND OMENTUM, DIAGNOSTIC, WITH COLLECTION OF SPECIMEN(S) BY BIOPSY	Yes
49320	SINGLE PORT LAPAROSCOPY, ABDOMEN, PERITONEUM, AND OMENTUM, DIAGNOSTIC, WITH COLLECTION OF SPECIMEN(S) BY WASHING	Yes
49321	LAPAROSCOPIC BIOPSY ABDOMEN	No
49321	LAPAROSCOPIC BIOPSY ABDOMEN	No
49321	LAPAROSCOPIC BIOPSY BOWEL SMALL	No
49321	LAPAROSCOPIC BIOPSY COLON	No
49321	LAPAROSCOPIC BIOPSY PELVIS	No
49321	LAPAROSCOPY OF PERITONEAL CAVITY W/ BIOPSY	No
49321	LAPAROSCOPY WITH BIOPSY	No
49322	ASPIRATION CYST OVARIAN	Yes
49322	LAPAROSCOPIC EXCISION CYST OVARIAN	Yes
49322	LAPAROSCOPIC EXCISION CYST OVARIAN	Yes
49322	LAPAROSCOPY ASPIRATION OF OVARIAN CYST	Yes
49322	LAPAROSCOPY W/ ASPIRATION CYST	Yes
49322	LAPAROSCOPY W/ ASPIRATION OF CAVITY	Yes
49324	LAPAROSCOPIC INSERTION OF INTRAPERITONEAL CANNULA OR CATHETER, PERMANENT	No
49324	LAPAROSCOPY W/ INSERTION OF TUNNELED INTRAPERITONEAL CATHETER	No
49324	LAPAROSCOPY W/ INSERTION OF TUNNELED INTRAPERITONEAL CATHETER	No
49329	LAP PANCREATECTOMY W/O PANCREATICOJEJUNOSTOMY W/O SPLENECTOMY	Yes
49329	LAPAROSCOPIC CREATION OF OMENTAL FLAP INTRA-ABDOMINAL	Yes
49329	LAPAROSCOPIC EVACUATION OF BLOOD CLOT ABDOMINAL	Yes
49329	LAPAROSCOPIC EXCISION OF LESION OF MESENTERY	Yes
49329	LAPAROSCOPIC EXCISION OR DESTRUCTION INTRA-ABDOMINAL TUMOR PRIMARY OR SECONDARY TUMORS LARGEST TUMOR 5 CM DIAMETER OR LESS	Yes
49329	LAPAROSCOPIC EXCISION PERITONEAL MASS	Yes
49329	LAPAROSCOPIC EXCISION SOFT SUBFACIAL TUMOR OF ABDOMINAL WALL	Yes

MSBOS - All records

Individual patient parameters (i.e., anemia, coagulopathy, transfusion risk) should be taken into consideration and may warrant an individual TSCR when not generally recommended.

June 22, 2023 **Sorted by CPT**

Primary CPT	Procedure Name	TSCR (Type and Screen) Needed?
49329	LAPAROSCOPIC EXISION OF OMENTAL CYST	Yes
49329	LAPAROSCOPIC EXPLORATION FOR POSTOPERATIVE HEMORRHAGE, THROMBOSIS OR INFECTION ABDOMEN	Yes
49329	LAPAROSCOPIC INGUINAL MESH EXPLANATION/REMOVAL	Yes
49329	LAPAROSCOPIC INSERTION CATHETER PERITONEAL DIALYSIS TEMPORARY	Yes
49329	LAPAROSCOPIC RELEASE OF MEDIAN ARCUATE LIGAMENT	Yes
49329	LAPAROSCOPIC RELEASE OF MEDIAN ARCUATE LIGAMENT	Yes
49329	LAPAROSCOPIC REMOVAL CATHETER PERITONEAL DIALYSIS	Yes
49329	LAPAROSCOPIC RETROPERITONEAL RESECTION OR DEBRIDEMENT OF PANCREAS AND PERIPANCREATIC TISSUE	Yes
49329	LAPAROSCOPY REMOVAL FOREIGN BODY IN OMENTUM/ABDOMEN	Yes
49329	LAPAROSCOPY, SURGICAL, LYSIS OF ADHESIONS UTERUS/OMETUM/ABDOMINAL WALL	Yes
49329	ROBOTIC REMOVAL OF RETROPERITONEAL MASS	Yes
49329	XI ROBOTIC MEDIAN ARCUATE LIGAMENT RELEASE	Yes
49329	XI ROBOTIC REMOVAL MASS; PELVIC	Yes
49422	REMOVAL OF PERMANENT INTRAPERITONEAL CATHETER	No
49441	INSERTION DUODENOSTOMY TUBE PERCUTANEOUS W/ FLUORO GUIDE	No
49441	INSERTION TUBE JEJUNOSTOMY FEEDING	No
49441	INSERTION TUBE JEJUNOSTOMY FEEDING	No
49505	HERNIORRHAPHY INGUINAL	No
49505	HERNIORRHAPHY INGUINAL ELECTIVE ADULT REDUCIBLE	No
49505	HERNIORRHAPHY INGUINAL ELECTIVE ADULT REDUCIBLE BILATERAL	No
49505	HERNIORRHAPHY INGUINAL INITIAL HERNIA >5 YRS REDUCIBLE (ELECTIVE)	No
49505	HERNIORRHAPHY INGUINAL REDUCIBLE, AGE 5 YEARS OR OLDER PEDIATRIC	No
49505	REPAIR INITIAL INGUINAL HERNIA = OR > AGE 5 REDUCIBLE LICHTENSTEIN APPROACH WITH MESH	No
49505	REPAIR INITIAL INGUINAL HERNIA = OR > AGE 5 REDUCIBLE W/ PRE PERITONEAL MESH	No
49505	REPAIR INITIAL INGUINAL HERNIA = OR > AGE 5 REDUCIBLE W/O MESH	No
49507	HERNIORRHAPHY INGUINAL ADULT INCARCERATED OR STRANGULATED	No
49507	HERNIORRHAPHY INGUINAL INCARCERATED	No
49507	HERNIORRHAPHY INGUINAL INCARCERATED OR STRANGULATED	No
49507	HERNIORRHAPHY INGUINAL INCARCERATED OR STRANGULATED, AGE 5 YEARS OR OLDER	No
49507	HERNIORRHAPHY INGUINAL INITIAL HERNIA >5 YRS INCARCERATED	No
49520	HERNIORRHAPHY INGUINAL RECURRENT REDUCIBLE	No
49520	HERNIORRHAPHY INGUINAL RECURRENT REDUCIBLE	No
49520	HERNIORRHAPHY INGUINAL RECURRENT REDUCIBLE ADULT	No
49525	HERNIORRHAPHY INGUINAL SLIDING HERNIA PEDIATRIC	No
49525	REPAIR HERNIA INGUINAL SLIDING	No
49525	REPAIR HERNIA INGUINAL SLIDING	No
49560	HERNIORRHAPHY INCISIONAL REDUCIBLE	No

MSBOS - All records

Individual patient parameters (i.e., anemia, coagulopathy, transfusion risk) should be taken into consideration and may warrant an individual TSCR when not generally recommended.
June 22, 2023 **Sorted by CPT**

Primary CPT	Procedure Name	TSCR (Type and Screen) Needed?
49560	HERNIORRHAPHY INCISIONAL ABDOMINAL ADULT INITIAL REDUCIBLE	No
49560	HERNIORRHAPHY INCISIONAL FLANK ADULT INITIAL REDUCIBLE	No
49560	HERNIORRHAPHY PEDIATRIC VENTRAL REDUCIBLE	No
49560	HERNIORRHAPHY RETRORECTUS APPROACH INITIAL REPAIR, REDUCIBLE HERNIA	No
49560	HERNIORRHAPHY VENTRAL ADULT INITIAL REDUCIBLE	No
49560	HERNIORRHAPHY VENTRAL PEDIATRIC	No
49560	REPAIR OF AN INCISIONAL HERNIA AT A FORMER PARASTOMAL SITE	No
49561	HERNIORRHAPHY INCISIONAL ABDOMINAL ADULT INITIAL INCARCERATED OR STRANGULATED	No
49561	HERNIORRHAPHY INCISIONAL INCARCERATED OR STRANGULATED	No
49561	HERNIORRHAPHY VENTRAL ADULT INCISIONAL OR VENTRAL INCARCERATED OR STRANGULATED	No
49565	HERNIORRHAPHY INCISIONAL ABDOMINAL ADULT RECURRENT REDUCIBLE	No
49565	HERNIORRHAPHY INCISIONAL ABDOMINAL PEDIATRIC RECURRENT REDUCIBLE	No
49565	HERNIORRHAPHY RECURRENT REDUCIBLE	No
49565	HERNIORRHAPHY RETRORECTUS APPROACH RECURRENT REPAIR, REDUCIBLE HERNIA	No
49565	HERNIORRHAPHY VENTRAL RECURRENT, REDUCIBLE	No
49566	HERNIORRHAPHY RETRORECTUS APPROACH RECURRENT REPAIR, STRANGULATED / INCARCERATED HERNIA	No
49566	REPAIR HERNIA, PEDIATRIC RECURRENT INCISIONAL, INCARCERATED OR STRANGULATED	No
49566	REPAIR HERNIA, RECURRENT INCISIONAL, INCARCERATED OR STRANGULATED	No
49566	REPAIR HERNIA, RECURRENT VENTRAL, INCARCERATED OR STRANGULATED	No
49566	REPAIR HERNIA, RECURRENT, INCARCERATED OR STRANGULATED	No
49568	IMPLANT MESH	No
49568	IMPLANT MESH CLOSURE OF DEBRIDEMENT FOR NECROTIZING SOFT TISSUE INFECT ABDOMEN	No
49568	IMPLANT MESH INCISIONAL HERNIA REPAIR	No
49568	IMPLANT MESH/PROSTHESIS INCISIONAL HERNIA REPAIR	No
49568	IMPLANT MESH/PROSTHESIS VENTRAL HERNIA REPAIR	No
49570	HERNIORRHAPHY EPIGASTRIC REDUCIBLE	No
49570	HERNIORRHAPHY EPIGASTRIC REDUCIBLE	No
49570	REPAIR HERNIA EPIGASTRIC REDUCIBLE, PEDIATRIC	No
49580	HERNIORRHAPHY UMBILICAL REDUCIBLE, < 5 YEARS	No
49580	HERNIORRHAPHY UMBILICAL REDUCIBLE, < 5 YEARS	No
49580	HERNIORRHAPHY UMBILICAL REDUCIBLE,INFANT	No
49585	HERNIORRHAPHY UMBILICAL	No
49585	HERNIORRHAPHY UMBILICAL ADULT	No
49585	HERNIORRHAPHY UMBILICAL REDUCIBILE, > 5 YEARS	No
49585	REPAIR HERNIA UMBILICAL REDUCIBLE, > 5 YEARS, < 18 YEARS	No
49585	REPAIR HERNIA UMBILICAL REDUCIBLE, > 5 YEARS, < 18 YEARS PEDIATRIC	No
49585	REPAIR UMBILICAL HERNIA, >5 YEARS, REDUCIBLE	No

MSBOS - All records

Individual patient parameters (i.e., anemia, coagulopathy, transfusion risk) should be taken into consideration and may warrant an individual TSCR when not generally recommended.

June 22, 2023 **Sorted by CPT**

Primary CPT	Procedure Name	TSCR (Type and Screen) Needed?
49587	REPAIR HERNIA UMBILICAL INCARCERATED OR STRANGULATED, > 5 YEARS	No
49650	HERNIORRHAPHY, INGUINAL	No
49650	LAPAROSCOPIC HERNIORRHAPHY, INGUINAL INITIAL	No
49650	LAPAROSCOPIC HERNIORRHAPHY, INGUINAL INITIAL BILATERAL	No
49650	LAPAROSCOPIC HERNIORRHAPHY, INGUINAL INITIAL PEDIATRIC	No
49650	LAPAROSCOPY SURGICAL REPAIR INITIAL INGUINAL HERNIA W/ MESH	No
49650	LAPAROSCOPY SURGICAL REPAIR INITIAL INGUINAL HERNIA WITH TOTAL EXTRA PERITONEAL REPAIR	No
49650	LAPAROSCOPY SURGICAL REPAIR INITIAL INGUINAL HERNIA WITH TRANS ABDOMINAL PRE-PERITONEAL REPAIR	No
49650	ROBOTIC INGUINAL HERNIA REPAIR	No
49650	SI ROBOTIC INGUINAL HERNIA REPAIR	No
49650	XI ROBOTIC INGUINAL HERNIA REPAIR	No
49651	LAPAROSCOPIC HERNIORRHAPHY INGUINAL RECURRENT BILATERAL	No
49651	LAPAROSCOPIC HERNIORRHAPHY, INGUINAL RECURRENT	No
49651	LAPAROSCOPIC HERNIORRHAPHY, INGUINAL RECURRENT	No
49651	LAPAROSCOPY SURGICAL REPAIR RECURRENT INGUINAL HERNIA W/ MESH	No
49651	LAPAROSCOPY SURGICAL REPAIR RECURRENT INGUINAL HERNIA WITH TRANS ABDOMINAL PRE-PERITONEAL REPAIR	No
49652	LAPAROSCOPIC FEMORAL HERNIORRHAPHY FOR REDUCIBLE HERNIA	No
49652	LAPAROSCOPIC HERNIORRHAPHY FOR REDUCIBLE UMBILICAL HERNIA	No
49652	LAPAROSCOPIC REPAIR HERNIA REDUCIBLE EPIGASTRIC W/MESH	No
49652	LAPAROSCOPIC REPAIR HERNIA REDUCIBLE SPIGELIAN W/MESH	No
49652	LAPAROSCOPIC REPAIR HERNIA REDUCIBLE UMBILICAL W/MESH	No
49652	LAPAROSCOPIC REPAIR HERNIA REDUCIBLE VENTRAL W/MESH	No
49652	LAPAROSCOPIC REPAIR VENTRAL HERNIA REDUCIBLE	No
49652	LAPAROSCOPIC REPAIR VENTRAL HERNIA REDUCIBLE	No
49652	ROBOTIC LAPAROSCOPIC REPAIR HERNIA REDUCIBLE VENTRAL W/MESH	No
49652	XI ROBOTIC LAPAROSCOPIC REPAIR HERNIA REDUCIBLE UMBILICAL W/MESH	No
49652	XI ROBOTIC LAPAROSCOPIC REPAIR HERNIA REDUCIBLE VENTRAL W/MESH	No
49653	LAPAROSCOPIC HERNIORRHAPHY FOR STRANGULATED / INCARCERATED HERNIA	No
49653	LAPAROSCOPIC FEMORAL HERNIORRHAPHY FOR STRANGULATED / INCARCERATED HERNIA	No
49653	LAPAROSCOPIC HERNIORRHAPHY FOR STRANGULATED / INCARCERATED UMBILICAL HERNIA	No
49653	LAPAROSCOPIC REPAIR EPIGASTRIC HERNIA INCARCERATED W/ MESH	No
49653	LAPAROSCOPIC REPAIR SPIGELIAN HERNIA INCARCERATED W/ MESH	No
49653	LAPAROSCOPIC REPAIR UMBILICAL HERNIA INCARCERATED W/ MESH	No
49653	LAPAROSCOPIC REPAIR UMBILICAL HERNIA INCARCERATED W/O MESH	No
49653	LAPAROSCOPIC REPAIR VENTRAL HERNIA INCARCERATED W/ MESH	No
49654	HERNIORRHAPHY INCISIONAL REDUCIBLE	No
49654	LAPAROSCOPIC HERNIORRHAPHY INCISIONAL ABDOMEN REDUCIBLE	No

MSBOS - All records

Individual patient parameters (i.e., anemia, coagulopathy, transfusion risk) should be taken into consideration and may warrant an individual TSCR when not generally recommended.

June 22, 2023 **Sorted by CPT**

Primary CPT	Procedure Name	TSCR (Type and Screen) Needed?
49654	LAPAROSCOPIC REPAIR HERNIA REDUCIBLE INCISIONAL W/MESH	No
49654	ROBOTIC INCISIONAL HERNIA REPAIR	No
49654	ROBOTIC LAPAROSCOPIC HERNIORRHAPHY INCISIONAL ABDOMEN REDUCIBLE	No
49655	HERNIORRHAPHY INCISIONAL W/ MESH FOR STRANGULATED / INCARCERATED INCISIONAL HERNIA	No
49655	LAPAROSCOPIC HERNIORRHAPHY INCISIONAL W/ MESH FOR STRANGULATED / INCARCERATED INCISIONAL HERNIA	No
49655	ROBOTIC LAPAROSCOPIC HERNIORRHAPHY INCISIONAL W/ MESH FOR STRANGULATED / INCARCERATED INCISIONAL HERNIA	No
49656	LAPAROSCOPIC INCISIONAL RECURRENT HERNIA REPAIR REDUCIBLE ADULT	No
49656	LAPAROSCOPIC INCISIONAL RECURRENT HERNIA REPAIR W/ MESH REDUCIBLE ADULT	No
49656	LAPAROSCOPIC INCISIONAL RECURRENT HERNIA REPAIR WO/ MESH REDUCIBLE ADULT	No
49999	EVACUATION OF RETROPERITONEAL SEROMA OPEN	No
49999	EXPLORATION ABDOMINAL WALL	No
49999	MESENTERIC BIOPSY	No
49999	REMOVAL FOREIGN BODY ABDOMINAL WALL	No
49999	REMOVAL FOREIGN BODY ABDOMINAL WALL	No
49999	REMOVE SUTURES ABDOMEN VIA INCISION UNDER ANESTHESIA	No
50080	PERCUTANEOUS NEPHROLITHOTOMY	No
50080	PERCUTANEOUS NEPHROLITHOTOMY	No
50080	PERCUTANEOUS NEPHROSTOLITHOTOMY UP TO 2 CM	No
50081	PERC NEPHROLITHOTOMY LITHOTRIPSY,STONE EXTRACTION,ANTEGRADE URETEROSCOPY,STENT PLACEMENT WHEN PERFORMED INCD IMAGING OVER 2cm	No
50081	PERCUTANEOUS NEPHROSTOLITHOTOMY OVER 2 CM	No
50081	PERCUTANEOUS NEPHROSTOLITHOTOMY OVER 2 CM	No
50220	NEPHRECTOMY ADULT	Yes
50220	NEPHRECTOMY ADULT	Yes
50220	NEPHRECTOMY INCL PART URETER OPEN APPROACH W/ RIB RESECT	Yes
50230	NEPHRECTOMY DISSECTION NODES RADICAL	Yes
50230	NEPHRECTOMY RADICAL	Yes
50230	NEPHRECTOMY RADICAL	Yes
50230	NEPHRECTOMY RADICAL PEDIATRIC	Yes
50230	RADICAL NEPHRECTOMY W/ PART URETERECTOMY	Yes
50230	RADICAL NEPHRECTOMY W/ PART URETERECTOMY W/ REG LYMPNODES RESECT	Yes
50230	RADICAL NEPHRECTOMY W/ PART URETERECTOMY W/ RIB & LYMPHNODE RESECTION	Yes
50240	NEPHRECTOMY PARTIAL ADULT	Yes
50360	RENAL ALLOTRANSPLANTATION W/ GRAFT W/O RECIPIENT NEPHRECTOMY	Yes
50543	LAPAROSCOPIC HAND ASSISTED NEPHRECTOMY PARTIAL	No
50543	LAPAROSCOPIC NEPHRECTOMY PARTIAL	No
50543	ROBOTIC LAPAROSCOPIC NEPHRECTOMY PARTIAL	No

MSBOS - All records

Individual patient parameters (i.e., anemia, coagulopathy, transfusion risk) should be taken into consideration and may warrant an individual TSCR when not generally recommended.
June 22, 2023 **Sorted by CPT**

Primary CPT	Procedure Name	TSCR (Type and Screen) Needed?
50543	ROBOTIC LAPAROSCOPIC NEPHRECTOMY PARTIAL	No
50543	SI ROBOTIC LAPAROSCOPIC NEPHRECTOMY PARTIAL	No
50543	XI ROBOTIC LAPAROSCOPIC NEPHRECTOMY PARTIAL	No
50544	PYELOPLASTY LAPAROSCOPIC	No
50544	ROBOTIC LAPAROSCOPIC PYELOPLASTY	No
50544	ROBOTIC LAPAROSCOPIC PYELOPLASTY	No
50544	ROBOTIC SINGLE PORT LAPAROSCOPIC PYELOPLASTY	No
50544	XI ROBOTIC LAPAROSCOPIC PYELOPLASTY	No
50545	LAPAROSCOPIC HAND ASSISTED NEPHRECTOMY	Yes
50545	LAPAROSCOPIC NEPHRECTOMY RADICAL	Yes
50545	NEPHRECTOMY RADICAL	Yes
50545	ROBOTIC LAPAROSCOPIC NEPHRECTOMY RADICAL	Yes
50545	ROBOTIC LAPAROSCOPIC RADICAL NEPHRECTOMY W/ REMOVAL OF LYMPH NODES & ADRENALECTOMY	Yes
50545	SI ROBOTIC LAPAROSCOPIC NEPHRECTOMY RADICAL	Yes
50545	XI ROBOTIC LAPAROSCOPIC NEPHRECTOMY RADICAL	Yes
50545	XI ROBOTIC LAPAROSCOPIC NEPHRECTOMY RADICAL RETROPERITONEAL APPROACH	Yes
50545	XI ROBOTIC RADICAL NEPHRECTOMY WITH IVC THROMBUS	Yes
50546	LAPAROSCOPIC HAND ASSISTED NEPHRECTOMY INCLUDING PARTIAL URETERECTOMY	Yes
50546	LAPAROSCOPIC NEPHRECTOMY W/ PARTIAL URETERECTOMY	Yes
50546	NEPHRECTOMY W/ PARTIAL URETERECTOMY	Yes
50546	ROBOTIC LAPAROSCOPIC NEPHRECTOMY W/ PARTIAL URETERECTOMY	Yes
50546	XI ROBOTIC LAPAROSCOPIC NEPHRECTOMY W/ PARTIAL URETERECTOMY	Yes
50547	NEPHRECTOMY DONOR, LIVING LAPAROSCOPIC	No
50548	LAPAROSCOPIC SURGICAL NEPHRECTOMY W/ TOTAL URETERECTOMY	Yes
50548	ROBOTIC LAPAROSCOPIC NEPHROURETERECTOMY ADULT	Yes
50548	ROBOTIC LAPAROSCOPIC SURGICAL NEPHRECTOMY W/ TOTAL URETERECTOMY	Yes
50548	ROBOTIC LAPAROSCOPIC SURGICAL NEPHRECTOMY W/ TOTAL URETERECTOMY	Yes
50548	XI ROBOTIC LAPAROSCOPIC NEPHROURETERECTOMY ADULT	Yes
50548	XI ROBOTIC LAPAROSCOPIC SURGICAL NEPHRECTOMY W/ TOTAL URETERECTOMY	Yes
50590	EXTRACORPOREAL SHOCKWAVE LITHOTRIPSY BILATERAL	No
50590	EXTRACORPOREAL SHOCKWAVE LITHOTRIPSY UNILATERAL	No
50590	EXTRACORPOREAL SHOCKWAVE LITHOTRIPSY UNILATERAL	No
50947	LAPAROSCOPY URETERONEOCYSTOSTOMY W/ CYSTOSCOPY AND URETERAL STENT PLACEMENT	No
50947	ROBOTIC LAPAROSCOPY URETERONEOCYSTOSTOMY W/ CYSTOSCOPY AND URETERAL STENT PLACEMENT	No
50947	ROBOTIC SINGLE PORT LAPAROSCOPY URETERONEOCYSTOSTOMY W/ CYSTOSCOPY AND URETERAL STENT PLACEMENT	No
50947	SI ROBOTIC LAPAROSCOPY URETERONEOCYSTOSTOMY W/ CYSTOSCOPY AND URETERAL STENT PLACEMENT	No
50947	URETERONEOCYSTOSTOMY W/ CYSTOSCOPY AND URETERAL STENT PLACEMENT	No

MSBOS - All records

Individual patient parameters (i.e., anemia, coagulopathy, transfusion risk) should be taken into consideration and may warrant an individual TSCR when not generally recommended.

June 22, 2023 **Sorted by CPT**

Primary CPT	Procedure Name	TSCR (Type and Screen) Needed?
50947	XI ROBOTIC LAPAROSCOPY URETERONEOCYSTOSTOMY W/ CYSTOSCOPY AND URETERAL STENT PLACEMENT	No
51040	CYSTOSTOMY OPEN	No
51040	CYSTOSTOMY/CYSTOTOMY	No
51040	CYSTOSTOMY/CYSTOTOMY	No
51102	INSERTION CATHETER SUPRAPUBIC	No
51590	CYSTECTOMY BLADDER RADICAL, CONDUIT ILEAL	Yes
51999	CYSTECTOMY BLADDER, CONDUIT ILEAL AND LYMPHADENECTOMY	Yes
51999	ENDOSCOPIC PANCREATIC NECROSECTOMY	Yes
51999	LAPAROSCOPIC COMPLETE CYSTECTOMY AND LYMPHADENECTOMY W/ SIGMOID BLADDER	Yes
51999	LAPAROSCOPIC COMPLETE CYSTECTOMY AND LYMPHADENECTOMY W/URETEROILEAL CONDUIT	Yes
51999	LAPAROSCOPIC REPAIR BLADDER	Yes
51999	LAPAROSCOPY SURGICAL BLADDER DIVERTICULECTOMY SINGLE OR MULTIPLE	Yes
51999	ROBOTIC LAPAROSCOPIC BLADDER CYSTOPLASTY AUGMENTATION	Yes
51999	ROBOTIC LAPAROSCOPIC CYSTECTOMY BLADDER, CONDUIT ILEAL AND LYMPHADENECTOMY	Yes
51999	ROBOTIC LAPAROSCOPIC CYSTECTOMY COMPLETE, W/CONTINENT DIVERSION, NEOBLADDER	Yes
51999	ROBOTIC LAPAROSCOPIC EXCISION BLADDER DIVERTICULUM,SINGLE OR MULTIPLE	Yes
51999	ROBOTIC LAPAROSCOPIC PELVIC EXENTENERATION W/ BILATERAL PELVIC NODE DISSECTION	Yes
51999	ROBOTIC LAPAROSCOPIC TRANSABDOMINAL REMOVAL OF MESH FROM BLADDER	Yes
51999	ROBOTIC LAPAROSCOPY CYSTECTOMY BLADDER PARTIAL SIMPLE	Yes
51999	ROBOTIC URETERONEOCYCTOSTOMY ANASTAMOSIS OF SINGLE URETER TO BLADDER	Yes
51999	Robotic Urethral Diverticulectomy	Yes
51999	SI ROBOTIC LAPAROSCOPIC EXCISION BLADDER DIVERTICULUM,SINGLE OR MULTIPLE	Yes
51999	XI ROBOTIC LAPAROSCOPIC CYSTECTOMY BLADDER, CONDUIT ILEAL AND LYMPHADENECTOMY	Yes
51999	XI ROBOTIC LAPAROSCOPIC CYSTECTOMY COMPLETE, W/CONTINENT DIVERSION, NEOBLADDER	Yes
51999	XI ROBOTIC LAPAROSCOPIC EXCISION BLADDER DIVERTICULUM,SINGLE OR MULTIPLE	Yes
51999	XI ROBOTIC LAPAROSCOPIC PELVIC EXENTENERATION W/ BILATERAL PELVIC NODE DISSECTION	Yes
51999	XI ROBOTIC LAPAROSCOPIC REPAIR BLADDER	Yes
51999	XI ROBOTIC LAPAROSCOPY CYSTECTOMY BLADDER PARTIAL SIMPLE	Yes
51999	XI ROBOTIC LAPARSCOPIC SUPRATRIGONAL CYSTECTOMY WITH URINARY DIVERSION	Yes
52000	CYSTOSCOPY	No
52000	CYSTOSCOPY	No
52000	CYSTOSCOPY FLEXIBLE	No
52000	CYSTOSCOPY PEDIATRIC	No
52000	CYSTOSCOPY RIGID	No
52000	CYSTOSCOPY W/CYSVIEW	No
52000	CYSTOSCOPY, URETEROSCOPY	No
52000	CYSTOSCOPY, URETEROSCOPY WITH FLUOROSCOPY	No

MSBOS - All records

Individual patient parameters (i.e., anemia, coagulopathy, transfusion risk) should be taken into consideration and may warrant an individual TSCR when not generally recommended.

June 22, 2023 **Sorted by CPT**

Primary CPT	Procedure Name	TSCR (Type and Screen) Needed?
52000	CYSTOURETHROSCOPY	No
52001	CYSTOURETHROSCOPY W/ EVACUATE CLOTS	Yes
52001	EVACUATION CLOT BLADDER	Yes
52001	EVACUATION CLOT BLADDER	Yes
52005	CYSTOSCOPY, RETROPYELOGRAM	No
52005	CYSTOSCOPY, RETROPYELOGRAM	No
52005	CYSTOURETHROSCOPY W/ URETERAL CATHETERIZATION,	No
52005	CYSTOURETHROSCOPY W/ URETERAL CATHETERIZATION, PEDIATRIC	No
52005	PYELOGRAM RETROGRADE	No
52204	BIOPSY URETHRA	No
52204	CYSTOSCOPY, BIOPSY BLADDER AND FULGURATION	No
52204	CYSTOSCOPY, BIOPSY BLADDER AND FULGURATION	No
52204	CYSTOURETHROSCOPY W/ BIOPSY(S)	No
52234	CYSTOURETHROSCOPY LASER EXCISION SMALL BLADDER TUMOR(S) 0.5 TO 2.0 CM ~ HOLMIUM	No
52234	CYSTOURETHROSCOPY, SMALL BLADDER TUMOR(S) 0.5-2.0 CM	No
52234	CYSTOURETHROSCOPY, W/FULGURATION SMALL BLADDER TUMOR(S) 0.5-2.0 CM	No
52234	EXCISION BLADDER TUMOR SMALL (0.5 UP TO 2.0 CM)	No
52234	LASER EXCISION CYST / MASS BLADDER TRANSURETHRAL (0.5 UP TO 2.0 CM) ~YAG	No
52235	CYSTOURETHROSCOPY LASER EXCISION MEDIUM BLADDER TUMOR(S) 2.0 TO 5.0 CM ~ HOLMIUM	No
52235	CYSTOURETHROSCOPY W/FULGURATION &/OR RESECTION MEDIUM BLADDER TUMOR(S) 2.0 - 5.0 CM W/SALINE BIPOLAR	No
52235	CYSTOURETHROSCOPY, W/FULGURATION MEDIUM BLADDER TUMOR(S) 2.0-5.0 CM	No
52235	LASER EXCISION CYST / MASS BLADDER TRANSURETHRAL (2.0 TO 5.0 CM) ~YAG	No
52235	RESECTION BLADDER TUMOR TRANSURETHRAL	No
52235	RESECTION BLADDER TUMOR TRANSURETHRAL	No
52240	CYSTOSCOPY W/ FULGURATION BLADDER TUMORS LARGE, > 5.0 CM	No
52240	CYSTOSCOPY W/ FULGURATION BLADDER TUMORS LARGE, > 5.0 CM	No
52240	CYSTOSCOPY W/ FULGURATION BLADDER TUMORS LARGE, > 5.0 CM PEDIATRIC	No
52240	CYSTOURETHROSCOPY LASER EXCISION LARGE BLADDER TUMOR(S) ~ HOLMIUM	No
52240	LASER EXCISION CYST / MASS BLADDER TRANSURETHRAL LARGE >5 CM ~YAG -	No
52260	CYSTOURETHROSCOPY W/ BLADDER DILATION FOR INTERSTITIAL CYSTITIS	No
52260	HYDRODISTENTION BLADDER	No
52260	HYDRODISTENTION BLADDER	No
52276	CYSTOURETHROSCOPY W/ DIRECT VISION INTERNAL URETHROTOMY	No
52276	CYSTOURETHROSCOPY W/ DIRECT VISION INTERNAL URETHROTOMY	No
52276	URETHROTOMY VISUAL INTERNAL	No
52281	CYSTOSCOPY, MEATOTOMY	No
52281	CYSTOURETHROSCOPY W/ CALIBRATION / DILATION OF STRICTURE W/ GRAM	No

MSBOS - All records

Individual patient parameters (i.e., anemia, coagulopathy, transfusion risk) should be taken into consideration and may warrant an individual TSCR when not generally recommended.
June 22, 2023 **Sorted by CPT**

Primary CPT	Procedure Name	TSCR (Type and Screen) Needed?
52281	CYSTOURETHROSCOPY W/ CALIBRATION / DILATION OF STRICTURE W/O GRAM	No
52281	DILATION URETHRAL	No
52281	DILATION URETHRAL	No
52287	CYSTOURETHROSCOPY W/INJECTION(S) FOR CHEMODENERVATION OF THE BLADDER	No
52310	CYSTOSCOPY W/ REMOVAL FOREIGN BODY, CALCULUS, URETERAL STENT FROM URETHRA OR BLADDER, SIMPLE	No
52310	CYSTOSCOPY W/ REMOVAL FOREIGN BODY, CALCULUS, URETERAL STENT FROM URETHRA OR BLADDER, SIMPLE PEDIATRIC	No
52310	CYSTOSCOPY, EXTRACTION STONE	No
52310	CYSTOURETHROSCOPY W/ REMOVE URETERAL STENT	No
52310	CYSTOURETHROSCOPY W/ REMOVE URETERAL STENT	No
52310	URETHERAL STENT REMOVAL	No
52317	CYSTOLITHOLAPAXY SIMPLE OR SMALL (LESS THAN 2.5 CM)	No
52317	LASER CYSTOLITHOLAPAXY BLADDER SIMPLE OR SMALL (LESS THAN 2.5 CM)	No
52317	LASER CYSTOLITHOLAPAXY BLADDER SIMPLE OR SMALL (LESS THAN 2.5 CM) ~ HOLMIUM	No
52317	LASER CYSTOLITHOLAPAXY BLADDER SIMPLE OR SMALL (LESS THAN 2.5 CM) ~ HOLMIUM	No
52317	REMOVAL STONE BLADDER SIMPLE OR SMALL (LESS THAN 2.5 CM)	No
52318	CYSTOLITHOLAPAXY COMPLICATED OR LARGE (OVER 2.5 CM)	No
52318	CYSTOLITHOLAPAXY COMPLICATED OR LARGE (OVER 2.5 CM)	No
52318	LASER CYSTOLITHOLAPAXY BLADDER COMPLICATED OR LARGE (OVER 2.5 CM)	No
52318	LASER CYSTOLITHOLAPAXY BLADDER COMPLICATED OR LARGE (OVER 2.5 CM) ~HOLMIUM	No
52318	REMOVAL STONE BLADDER COMPLICATED OR LARGE (OVER 2.5 CM)	No
52332	CYSTOSCOPY, INSERTION STENT URETERAL J	No
52332	CYSTOSCOPY, INSERTION STENT URETERAL J	No
52332	INSERTION STENT DOUBLE J	No
52332	INSERTION STENT URETERAL	No
52351	CYSTOURETHROSCOPY W/URETEROSCOPY AND/OR PYELOSCOPY DIAG	No
52351	CYSTOURETHROSCOPY W/URETEROSCOPY AND/OR PYELOSCOPY DIAG	No
52351	CYSTOURETHROSCOPY W/URETEROSCOPY AND/OR PYELOSCOPY DIAG PEDIATRIC	No
52351	URETEROSCOPY FLEXIBLE	No
52351	URETEROSCOPY RIGID	No
52352	CYSTOSCOPY WITH URETEROSCOPY WITH REMOVAL OR MANIPULATION OF URETERAL CALCULI	No
52353	CYSTOURETHROSCOPY W/ URETEROSCOPY AND/OR PYELOSCOPY W/ LITHOTRIPSY	No
52353	LASER CYSTOURETHROSCOPY W/ URETEROSCOPY AND/OR PYELOSCOPY W/ LITHOTRIPSY HOLMIUM	No
52353	LASER CYSTOURETHROSCOPY W/ URETEROSCOPY AND/OR PYELOSCOPY W/ LITHOTRIPSY HOLMIUM	No
52353	LASER LITHOTRIPSY STONES URETER ~HOLMIUM	No
52353	LASER LITHOTRISPY STONES URETER	No
52354	CYSTOURETHROSCOPY W/URETEROSCOPY AND BIOPSY	No
52354	CYSTOURETHROSCOPY W/URETEROSCOPY AND/OR PYELOSCOPY W/ BIOPSY AND/OR FULGURATION URETERAL OR RENAL PELVIC LESION	No

MSBOS - All records

Individual patient parameters (i.e., anemia, coagulopathy, transfusion risk) should be taken into consideration and may warrant an individual TSCR when not generally recommended.

June 22, 2023 **Sorted by CPT**

Primary CPT	Procedure Name	TSCR (Type and Screen) Needed?
52354	CYSTOURETHROSCOPY W/URETEROSCOPY AND/OR PYELOSCOPY W/ BIOPSY AND/OR FULGURATION URETERAL OR RENAL PELVIC LESION	No
52356	CYSTOURETHROSCOPY W/ URETEROSCOPY AND/OR PYELOSCOPY W/ LITHOTRIPSY INCLUDE INSERTION OF INDWELLING URETERAL STENT	No
52441	CYSTOURETHROSCOPY W/INSERTION OF PERM ADJ TRANSPROSTATIC IMPLANT; SINGLE IMPLANT	No
52500	INCISION BLADDER NECK	No
52500	INCISION BLADDER NECK	No
52500	RESECTION BLADDER NECK TRANSURETHRAL	No
52500	RESECTION BLADDER NECK TRANSURETHRAL PEDIATRIC	No
52601	CYSTOSCOPY, MANIPULATION STONE, TRANSURETHRAL RESECTION PROSTATE	No
52601	CYSTOSCOPY, RESECTION PROSTATE TRANSURETHRAL	No
52601	TRANSURETHRAL ELECTROSURGICAL COMPLETE VAPORIZATION OF PROSTATE	No
52601	TURP 1ST STAGE OF 2 STAGE RESECTION	No
52601	TURP COMPLETE	No
52601	TURP COMPLETE	No
52601	TURP COMPLETE W/ SALINE BIPOLAR	No
52601	TURP W/ CYSTOURETHROSCOPY COMPLETE	No
52648	LASER PROSTATECTOMY	No
52648	LASER PROSTATECTOMY ~ GREEN LIGHT	No
52648	LASER VAPORIZATION OF PROSTATE ~ XPS	No
52648	LASER VAPORIZATION OF PROSTATE ~ XPS	No
52648	LASER VAPORIZATION OF PROSTATE ~HPS	No
52648	LASER VAPORIZATION OF PROSTATE-DIODE	No
52649	LASER ENUCLEATION PROSTATE WITH MORCELLATION	No
53410	URETHROPLASTY ADULT	No
53410	URETHROPLASTY ANTERIOR 1-STAGE PROCEDURE, ADULT MALE	No
53410	URETHROTOMY/ URETHROSTOMY PERINEAL URETHRA, EXTERNAL	No
53445	IMPLANT SPHINCTER URINARY	No
53445	INSERTION INFLATABLE URETHRAL BLADDER NECK SPHINCTER W/ PUMP,RESV & CUFF	No
53445	INSERTION INFLATABLE URETHRAL BLADDER NECK SPHINCTER W/ PUMP,RESV & CUFF	No
53899	BIOPSY BLADDER	No
53899	BIOPSY BLADDER	No
53899	BLADDER CYSTOPLASTY AUGMENTATION	No
53899	LOOPOSCOPY, LOOP-O-GRAM	No
53899	REMOVAL CATHETER SUPRAPUBIC	No
53899	ROBOTIC DIVERTICULECTOMY BLADDER	No
54161	CIRCUMCISION	No
54161	CIRCUMCISION ADULT	No

MSBOS - All records

Individual patient parameters (i.e., anemia, coagulopathy, transfusion risk) should be taken into consideration and may warrant an individual TSCR when not generally recommended.

June 22, 2023 **Sorted by CPT**

Primary CPT	Procedure Name	TSCR (Type and Screen) Needed?
54161	CIRCUMCISION RITUAL PEDIATRIC	No
54161	CIRCUMCISION SURGICAL EXCISION CHILD OLDER THAN 28 DAYS AGE	No
54405	INSERTION PROSTHESIS PENILE INFLATABLE MULTI-COMPONENT	No
54410	REMOVAL - REPLACE PROSTHESIS PENILE RIGID MULTI- COMPONENT, INFLATABLE SAME OPER SESSION	No
54520	ORCHIECTOMY	No
54520	ORCHIECTOMY SIMPLE W/ SCROTAL OR INGUINAL APPROACH	No
54520	ORCHIECTOMY SIMPLE W/ SCROTAL OR INGUINAL APPROACH	No
54530	ORCHIECTOMY RADICAL	No
54640	ORCHIOPEXY INGUINAL OR SCROTAL APPROACH	No
54640	ORCHIOPEXY INGUINAL OR SCROTAL APPROACH	No
54640	ORCHIOPEXY INGUINAL OR SCROTAL APPROACH PEDIATRIC	No
55040	EXCISION HYDROCELE, UNILATERAL ADULT	No
55040	EXCISION HYDROCELE, UNILATERAL, PEDIATRIC	No
55040	HYDROCELECTOMY	No
55040	HYDROCELECTOMY ADULT	No
55040	HYDROCELECTOMY PEDIATRIC	No
55040	SPERMATOCELECTOMY UNILATERAL	No
55250	VASECTOMY	No
55700	BIOPSY PROSTATE	No
55700	BIOPSY PROSTATE	No
55700	BIOPSY PROSTATE; NEEDLE	No
55700	NEEDLE ASPIRATION / ASPIRATION BIOPSY PROSTATE WITH ULTRASOUND	No
55706	BIOPSIES, PROSTATE, NEEDLE, TRANSPERINEAL, STEREOTACTIC TEMPLATE GUIDED SATURATION SAMPLING, INCLUDING IMAGING GUIDANCE	No
55866	LAPAROSCOPIC SURGICAL RETROPUBIC RADICAL PROSTATECTOMY W/ NERVE SPARING	No
55866	RETROPUBIC RADICAL PROSTATECTOMY W/ NERVE SPARING	No
55866	ROBOTIC LAPAROSCOPIC RETROPUBIC RADICAL PROSTATECTOMY W/ NERVE SPARING	No
55866	ROBOTIC SINGLE PORT LAPAROSCOPIC RETROPUBIC RADICAL PROSTATECTOMY W/ NERVE SPARING	No
55866	XI ROBOTIC LAPAROSCOPIC RETROPUBIC RADICAL PROSTATECTOMY W/ NERVE SPARING	No
55874	TRANSPERINEAL PLACEMENT BIODEGRADABLE MATERIAL,PERI-PROSTATIC,SINGLE OR MULTIPLE INJECTION(S),INCLUDES IMAGE GUIDANCE WHEN PERFORMED	No
55875	INSERTION TRANSPERINEAL NEEDLES OR CATHETERS PROSTATE FOR INTERSTITIAL RADIOELEMENT APPLICATION	No
55899	EPIDIDYMOSTOMY	No
55899	EXAM UNDER ANESTHESIA MALE GENITALS	No
55899	EXCISION MASS PERINEUM MALE	No
55899	EXCISION OF SPERMATIC CORD	No
55899	PROSTATECTOMY RETROPUBIC SUBTOTAL	No

MSBOS - All records

Individual patient parameters (i.e., anemia, coagulopathy, transfusion risk) should be taken into consideration and may warrant an individual TSCR when not generally recommended.

June 22, 2023 **Sorted by CPT**

Primary CPT	Procedure Name	TSCR (Type and Screen) Needed?
55899	ROBOTIC LAPAROSCOPIC PROSTATECTOMY RETROPUBIC SUBTOTAL	No
55899	ROBOTIC LAPAROSCOPIC PROSTATECTOMY SUPRAPUBIC SUBTOTAL 1 OR 2 STAGES	No
55899	ROBOTIC SINGLE PORT LAPAROSCOPIC PROSTATECTOMY RETROPUBIC SUBTOTAL	No
55899	ROBOTIC SINGLE PORT LAPAROSCOPIC PROSTATECTOMY SUPRAPUBIC SUBTOTAL 1 OR 2 STAGES	No
55899	SI ROBOTIC LAPAROSCOPIC PROSTATECTOMY RETROPUBIC SUBTOTAL	No
55899	SPERMATIC CORD DENERVATION	No
55899	XI ROBOTIC LAPAROSCOPIC PROSTATECTOMY RETROPUBIC SUBTOTAL	No
55899	XI ROBOTIC LAPAROSCOPIC PROSTATECTOMY SUPRAPUBIC SUBTOTAL 1 OR 2 STAGES	No
56620	VULVECTOMY PARTIAL SIMPLE	No
57155	INSERT UTERINE TANDEMS FOR CLINICAL BRACHYTHERAPY	No
57155	INSERT UTERINE TANDEMS FOR CLINICAL BRACHYTHERAPY	No
57155	INSERT UTERINE VAGINAL OVOIDS FOR CLINICAL BRACHYTHERAPY	No
57240	ANTERIOR COLPORRHAPHY REPAIR CYSTOCELE W/O REPAIR URETHROCELE INCLUDING CYSTOURETHROSCOPY WHEN PERFORMED	No
57240	ANTERIOR COLPORRHAPHY REPAIR CYSTOCELE W/REPAIR URETHROCELE INCLUDING CYSTOURETHROSCOPY WHEN PERFORMED	No
57240	ANTERIOR COLPORRHAPHY REPAIR CYSTOCELE WITH OR W/O REPAIR URETHROCELE INCLUDING CYSTOURETHROSCOPY WHEN PERFORMED	No
57240	ANTERIOR COLPORRHAPHY REPAIR CYSTOCELE WITH OR W/O REPAIR URETHROCELE INCLUDING CYSTOURETHROSCOPY WHEN PERFORMED	No
57250	COLPORRHAPHY POSTERIOR	No
57250	POSTERIOR COLPORRHAPHY W/ REPAIR RECTOCELE AND PERINEORRHAPHY	No
57250	POSTERIOR COLPORRHAPHY W/ REPAIR RECTOCELE AND PERINEORRHAPHY	No
57250	POSTERIOR COLPORRHAPHY W/ REPAIR RECTOCELE W/O PERINEORRHAPHY	No
57260	COMBINED ANTERIOPOSTERIOR COLPORRHAPHY INCLUDING CYSTOURETHROSCOPY WHEN PERFORMED	No
57282	COLPOPEXY VAGINAL EXTRA-PERITONEAL	No
57282	COLPOPEXY VAGINAL EXTRA-PERITONEAL	No
57282	FIXATION LIGAMENT SACROSPINOUS	No
57283	COLPOPEXY	No
57283	COLPOPEXY VAGINAL VAULT SUSPENSION INTRA-PERITONEAL APPROACH	No
57283	COLPOPEXY VAGINAL VAULT SUSPENSION INTRA-PERITONEAL APPROACH	No
57287	INCISION SLING VAGINAL	No
57287	REMOVAL SUSPENSION BLADDER TRANSVAGINAL TAPE	No
57287	REMOVE SLING TX FOR STRESS INCONTINENCE FEMALE	No
57287	REMOVE SLING TX FOR STRESS INCONTINENCE FEMALE	No
57287	REVISE SLING TX FOR STRESS INCONTINENCE FEMALE	No
57288	PUBO VAGINAL SLING W/ RECTUS FASCIA GRAFT	No

MSBOS - All records

Individual patient parameters (i.e., anemia, coagulopathy, transfusion risk) should be taken into consideration and may warrant an individual TSCR when not generally recommended.
June 22, 2023 **Sorted by CPT**

Primary CPT	Procedure Name	TSCR (Type and Screen) Needed?
57288	SLING SURGERY FOR STRESS INCONTINENCE W/ TENSION FREE VAG TAPE	No
57288	SLING SURGERY FOR STRESS INCONTINENCE W/ TENSION FREE VAG TAPE	No
57288	SUSPENSION BLADDER SLING	No
57288	SUSPENSION BLADDER SLING TAPE TRANSOBTURATOR	No
57288	SUSPENSION BLADDER SLING TAPE TRANSVAGINAL	No
57288	URETHRAL SLING FOR STRESS INCONTINENCE OPEN	No
57410	EXAM UNDER ANESTHESIA PELVIC / VAGINAL	No
57425	COLPOPEXY LAPAROSCOPIC	No
57425	ROBOTIC LAPAROSCOPIC COLPOPEXY	No
57425	ROBOTIC LAPAROSCOPIC COLPOPEXY	No
57425	SI ROBOTIC LAPAROSCOPIC COLPOPEXY	No
57425	XI ROBOTIC LAPAROSCOPIC COLPOPEXY	No
57461	COLPOSCOPY CERVIX INCL UPPER/ADJACENT VAGINA W/ LEEP	No
57520	BIOPSY CERVICAL CONE COLD KNIFE	No
57520	BIOPSY CERVICAL CONE COLD KNIFE	No
57520	COLD KNIFE CONIZATION OF UTERINE CERVIX W/ D&C	No
57520	CONIZATION OF CERVIX W/W/O DILATION AND CURETTAGE COLD KNIFE OR LASER	No
57522	BIOPSY CERVICAL CONE LEEP	No
57522	LEEP LOOP CONIZATION OF UTERINE CERVIX W/ D&C	No
57522	LEEP PROCEDURE	No
57522	LEEP PROCEDURE	No
58120	DILATION AND CURETTAGE	No
58120	DILATION AND CURETTAGE	No
58120	DILATION AND CURETTAGE, REMOVAL DEVICE INTRAUTERINE	No
58120	DILATION AND CURETTAGE, SUCTION	No
58146	MYOMECTOMY EXCISION FIBROID TUMOR(S) > 5 W/ TOTAL WEIGHT < 250 G AND/OR REMOVAL SURFACE MYOMAS, ABDOMINAL	Yes
58146	MYOMECTOMY EXCISION FIBROID TUMOR(S) > 5 W/ TOTAL WEIGHT > 250 G	Yes
58146	MYOMECTOMY EXCISION FIBROID TUMOR(S) > 5 W/ TOTAL WEIGHT > 250 G AND/OR REMOVAL SURFACE MYOMAS, ABDOMINAL	Yes
58146	MYOMECTOMY EXCISION FIBROID TUMOR(S) 5 OR MORE W/ TOTAL WEIGHT < 250 GM, ABDOMINAL	Yes
58150	HYSTERECTOMY ABDOMINAL TOTAL	Yes
58150	HYSTERECTOMY ABDOMINAL TOTAL	Yes
58150	HYSTERECTOMY ABDOMINAL TOTAL WITH SALPINGO-OOPHORECTOMY BILATERAL	Yes
58150	HYSTERECTOMY ABDOMINAL TOTAL WITH SALPINGO-OOPHORECTOMY UNILATERAL	Yes
58260	HYSTERECTOMY VAGINAL UTERUS 250G OR LESS	No
58262	HYSTERECTOMY VAGINAL UTERUS 250G OR LESS REMOVAL TUBE(S) AND/OR OVARY(S)	No
58291	VAGINAL HYSTERECTOMY W/ REMOVAL OF TUBE(S) AND/OR OVARY(S), UTERUS GREATER THAN 250 G	No
58300	INSERTION DEVICE INTRAUTERINE	No

MSBOS - All records

Individual patient parameters (i.e., anemia, coagulopathy, transfusion risk) should be taken into consideration and may warrant an individual TSCR when not generally recommended.

June 22, 2023 **Sorted by CPT**

Primary CPT	Procedure Name	TSCR (Type and Screen) Needed?
58545	LAPAROSCOPIC MYOMECTOMY 1 TO 4 MYOMAS	No
58545	LAPAROSCOPIC MYOMECTOMY 1 TO 4 MYOMAS	No
58545	ROBOTIC LAPAROSCOPIC MYOMECTOMY; 1 TO 4 MYOMAS; TOTAL WEIGHT OF 250 G OR LESS	No
58545	XI ROBOTIC LAPAROSCOPIC MYOMECTOMY; 1 TO 4 MYOMAS; TOTAL WEIGHT OF 250 G OR LESS	No
58552	HYSTERECTOMY VAGINAL UTERUS 250 G OR LESS; REMOVAL - TUBE(S) AND/OR OVARY(S)	No
58552	LAPAROSCOPIC ASSISTED HYSTERECTOMY VAGINAL UTERUS 250 G OR LESS; REMOVAL - TUBE(S) AND/OR OVARY(S)	No
58552	ROBOTIC LAPAROSCOPIC ASSISTED HYSTERECTOMY VAGINAL UTERUS 250 G OR LESS; REMOVAL - TUBE(S) AND/OR OVARY(S)	No
58555	HYSTEROSCOPY	No
58558	HYSTEROSCOPY SURGICAL W/SAMPLING BIOPSY OF ENDOMETRIUM &/OR POLPYECTOMY W/ OR W/O D&C (MYOSURE)	No
58558	HYSTEROSCOPY SURGICAL W/SAMPLING BIOPSY OF ENDOMETRIUM &/OR POLYPECTOMY W/ OR W/O D&C (TRUCLEAR)	No
58558	HYSTEROSCOPY SURGICAL W/SAMPLING BIOPSY OF ENDOMETRIUM &/OR POLYPECTOMY W/ OR W/O D&C (TRUCLEAR)	No
58558	HYSTEROSCOPY W/ BIOPSY OF ENDOMETRIUM W/ D&C	No
58558	HYSTEROSCOPY W/ BIOPSY OF ENDOMETRIUM W/O D&C	No
58558	HYSTEROSCOPY W/ POLYPECTOMY W/ D&C	No
58558	HYSTEROSCOPY W/ POLYPECTOMY W/O D&C	No
58558	HYSTEROSCOPY, D & C	No
58558	HYSTEROSCOPY, SURGICAL; WITH SAMPLING BIOPSY OF ENDOMETRIUM AND/OR POLYPECTOMY WITH D&C	No
58558	HYSTEROSCOPY, SURGICAL; WITH SAMPLING BIOPSY OF ENDOMETRIUM AND/OR POLYPECTOMY WITHOUT D&C	No
58558	POLYPECTOMY UTERINE	No
58561	HYSTEROSCOPY W/ REMOVAL LEIOMYOMATA	No
58561	HYSTEROSCOPY W/ REMOVAL LEIOMYOMATA	No
58561	HYSTEROSCOPY W/ REMOVAL LEIOMYOMATA W/ MYOSURE	No
58562	HYSTEROSCOPY W/ REMOVAL IMPACTED FOREIGN BODY	No
58562	REMOVE IUD EMBEDDED W/ HYSTEROSCOPY	No
58562	REMOVE IUD EMBEDDED W/ HYSTEROSCOPY	No
58563	HYSTEROSCOPY W/ ENDOMETRIAL ABLATION PLASMA	No
58563	HYSTEROSCOPY WITH ENDOMETRIAL ABLATION	No
58563	HYSTEROSCOPY WITH ENDOMETRIAL ABLATION	No
58563	HYSTEROSCOPY, ABLATION ENDOMETRIAL NOVASURE	No
58570	HYSTERECTOMY TOTAL FOR UTERUS 250G OR LESS	No
58570	LAPAROSCOPIC HYSTERECTOMY TOTAL FOR UTERUS 250G OR LESS	No
58570	ROBOTIC LAPAROSCOPIC TOTAL HYSTERECTOMY, FOR UTERUS 250 G OR LESS	No
58570	XI ROBOTIC LAPAROSCOPIC TOTAL HYSTERECTOMY, FOR UTERUS 250 G OR LESS	No
58571	HYSTERECTOMY TOTAL FOR UTERUS 250 G OR LESS W/REMOVAL TUBE(S) AND/OR OVARY(S)	Yes if Hgb less than 9
58571	LAPAROSCOPIC HYSTERECTOMY TOTAL FOR UTERUS 250 G OR LESS W/REMOVAL TUBE(S) AND/OR OVARY(S)	Yes if Hgb less than 9
58571	ROBOTIC LAPAROSCOPIC TOTAL HYSTERECTOMY W/ BILATERAL OOPHERECTOMY UTERUS=<250G	Yes if Hgb less than 9
58571	ROBOTIC LAPAROSCOPIC TOTAL HYSTERECTOMY W/ BILATERAL SALPINGECTOMY UTERUS=<250G	Yes if Hgb less than 9

MSBOS - All records

Individual patient parameters (i.e., anemia, coagulopathy, transfusion risk) should be taken into consideration and may warrant an individual TSCR when not generally recommended.

June 22, 2023 **Sorted by CPT**

Primary CPT	Procedure Name	TSCR (Type and Screen) Needed?
58571	ROBOTIC LAPAROSCOPIC TOTAL HYSTERECTOMY W/ BSO UTERUS=<250G	Yes if Hgb less than 9
58571	ROBOTIC LAPAROSCOPIC TOTAL HYSTERECTOMY W/ UNILAT REMOVE TUBES & OVARY UTERUS=<250G	Yes if Hgb less than 9
58571	ROBOTIC LAPAROSCOPIC TOTAL HYSTERECTOMY W/ UNILATERAL OOPHERECTOMY UTERUS=<250G	Yes if Hgb less than 9
58571	ROBOTIC SINGLE PORT LAPAROSCOPIC HYSTERECTOMY TOTAL FOR UTERUS 250 G OR LESS W/REMOVAL TUBE(S) AND/OR OVARY(S)	Yes if Hgb less than 9
58571	SINGLE PORT LAPAROSCOPIC HYSTERECTOMY TOTAL FOR UTERUS 250 G OR LESS W/REMOVAL TUBE(S) AND/OR OVARY(S)	Yes if Hgb less than 9
58571	XI ROBOTIC LAPAROSCOPIC TOTAL HYSTERECTOMY W/ BILATERAL OOPHERECTOMY UTERUS=<250G	Yes if Hgb less than 9
58571	XI ROBOTIC LAPAROSCOPIC TOTAL HYSTERECTOMY W/ BILATERAL SALPINGECTOMY UTERUS=<250G	Yes if Hgb less than 9
58571	XI ROBOTIC LAPAROSCOPIC TOTAL HYSTERECTOMY W/ BSO UTERUS=<250G	Yes if Hgb less than 9
58571	XI ROBOTIC LAPAROSCOPIC TOTAL HYSTERECTOMY W/ UNILAT REMOVE TUBES & OVARY UTERUS=<250G	Yes if Hgb less than 9
58571	XI ROBOTIC LAPAROSCOPIC TOTAL HYSTERECTOMY W/ UNILATERAL SALPINGECTOMY UTERUS=<250G	Yes if Hgb less than 9
58571	XI ROBOTIC SINGLE PORT LAPAROSCOPIC HYSTERECTOMY TOTAL FOR UTERUS 250 G OR LESS W/REMOVAL TUBE(S) *	Yes if Hgb less than 9
58573	HYSTERECTOMY TOTAL FOR UTERUS GREATER THAN 250G W/REMOVAL TUBE(S) AND/OR OVARY(S	Yes if Hgb less than 9
58573	LAPAROSCOPIC HYSTERECTOMY TOTAL FOR UTERUS GREATER THAN 250G W/REMOVAL TUBE(S) AND/OR OVARY(S	Yes if Hgb less than 9
58573	ROBOTIC LAPAROSCOPIC TOTAL HYSTERECTOMY W/ BSO UTERUS=>250G	Yes if Hgb less than 9
58661	LAPAROSCOPIC OOPHORECTOMY	No
58661	LAPAROSCOPIC SALPINGECTOMY	No
58661	LAPAROSCOPY WITH OOPHORECTOMY AND SALPINGECTOMY	No
58661	LAPAROSCOPY WITH OOPHORECTOMY AND SALPINGECTOMY	No
58661	LAPAROSCOPY WITH REMOVE ADNEXA	No
58661	ROBOTIC LAPAROSCOPY REMOVAL ADNEXAL STRUCTURES	No
58661	SINGLE PORT LAPAROSCOPY WITH OOPHORECTOMY AND SALPINGECTOMY	No
58661	XI ROBOTIC LAPAROSCOPY W/ OOPHORECTOMY AND SALPINGECTOMY	No
58662	LAPAROSCOPY FULGURATION OR EXCISION OF LESIONS OF THE OVARY PELVIC VISCERA OR PERITONEAL SURFACE BY ANY METHOD	No
58662	LAPAROSCOPY LASER ABLATION ENDOMETRIOSIS ~ YAG	No
58662	LAPAROSCOPY WITH EXCISION LESIONS	No
58662	LAPAROSCOPY WITH EXCISION OF ENDOMETRIAL LESIONS PELVIS	No
58662	LAPAROSCOPY WITH EXCISION OF ENDOMETRIAL LESIONS PELVIS	No
58662	LAPAROSCOPY WITH FULGURATION OR EXCISION OF LESIONS OF THE OVARY	No
58662	LAPAROSCOPY, SURGICAL; WITH FULGURATION OR EXCISION OF LESIONS OF THE OVARY, PELVIC VISCERA, OR PERITONEAL SURFACE BY ANY METHOD	No
58662	LAPROSCOPIC OVARY CYSTECTOMY	No
58662	LASER ENDOMETRIOSIS ~ CO2	No
58662	ROBOTIC FULGURATION OR EXCISION OF LESIONS OF THE OVARY PELVIC VISCERA OR PERITONEAL SURFACE BY ANY METHOD	No
58662	XI ROBOTIC LAPAROSCOPIC MOBILIZATION OF SIGMOID COLON AND RECTUM WITH PRESACRAL DISSECTION	No
58953	DEBULKING SALPINGO-OOPHORECTOMY, OMENTECTOMY, TOTAL ABDOMINAL HYSTERECTOMY AND RADICAL DISSECTION, BILATERAL	Yes

MSBOS - All records

Individual patient parameters (i.e., anemia, coagulopathy, transfusion risk) should be taken into consideration and may warrant an individual TSCR when not generally recommended.

June 22, 2023 **Sorted by CPT**

Primary CPT	Procedure Name	TSCR (Type and Screen) Needed?
59151	LAPAROSCOPIC REMOVAL ECTOPIC PREGNANCY W/ OOPHORECTOMY	No
59151	LAPAROSCOPIC REMOVAL ECTOPIC PREGNANCY W/ SALPINGECTOMY	No
59151	LAPAROSCOPIC REMOVAL ECTOPIC PREGNANCY W/ SALPINGECTOMY AND OOPHORECTOMY	No
59151	LAPAROSCOPIC REMOVAL ECTOPIC PREGNANCY W/ SALPINGECTOMY AND OOPHORECTOMY	No
59812	DILATION AND CURETTAGE, SUCTION INCOMPLETE ABORTION, ANY TRIMESTER	Yes
59820	DILATION AND CURETTAGE, SUCTION MISSED ABORTION, 1ST TRIMESTER	No
59820	DILATION AND CURETTAGE, SUCTION MISSED ABORTION, 1ST TRIMESTER	No
59820	TREATMENT OF MISSED AB COMPLETED SURGICALLY D&C, 1ST TRIMESTER	No
60220	EXCISION CYST / MASS THYROID	No
60220	EXCISION OF CYST / ADENOMA OF THYROID	No
60220	LOBECTOMY THYROID TOTAL, UNILATERAL; WITH OR WITHOUT ISTHMUSECTOMY	No
60220	TRANSECT ISTHMUS	No
60240	THYROIDECTOMY TOTAL	No
60252	THYROIDECTOMY SUBTOTAL	No
60252	THYROIDECTOMY,TOTAL OR SUBTOTAL FOR MALIGNANCY; WITH LIMITED NECK DISSECTION	No
60252	THYROIDECTOMY,TOTAL OR SUBTOTAL FOR MALIGNANCY; WITH LIMITED NECK DISSECTION	No
60260	THYROIDECTOMY AFTER PARTIAL THYROIDECTOMY	No
60260	THYROIDECTOMY COMPLETE FOLLOWING PARTIAL REMOVAL	No
60260	THYROIDECTOMY COMPLETE FOLLOWING PARTIAL REMOVAL	No
60271	THYROIDECTOMY INCLUD SUBSTERNAL THYROID, CERVICAL APPROACH	No
60271	THYROIDECTOMY INCLUD SUBSTERNAL THYROID, CERVICAL APPROACH	No
60271	THYROIDECTOMY, CERVICAL APPROACH.	No
60500	EXPLORATION PARATHYROID	No
60500	PARATHYROIDECTOMY	No
60500	PARATHYROIDECTOMY	No
60500	ROBOTIC PARATHYROIDECTOMY OR EXPLORATION OF PARATHYROID(S)	No
60650	ADRENALECTOMY	No
60650	LAPAROSCOPIC ADRENALECTOMY	No
60650	ROBOTIC LAPAROSCOPIC ADRENALECTOMY DORSAL APPROACH	No
60650	ROBOTIC LAPAROSCOPIC LEFT TRANSABDOMINAL ADRENALECTOMY	No
60650	ROBOTIC LAPAROSCOPIC RIGHT TRANSABDOMINAL ADRENALECTOMY	No
60650	SI ROBOTIC LAPAROSCOPIC ADRENALECTOMY DORSAL APPROACH	No
60650	SI ROBOTIC LAPAROSCOPIC RIGHT TRANSABDOMINAL ADRENALECTOMY	No
60650	XI ROBOTIC LAPAROSCOPIC ADRENALECTOMY DORSAL APPROACH	No
60650	XI ROBOTIC LAPAROSCOPIC LEFT TRANSABDOMINAL ADRENALECTOMY	No
60650	XI ROBOTIC LAPAROSCOPIC RIGHT RETROPERITONEAL ADRENALECTOMY	No
60650	XI ROBOTIC LAPAROSCOPIC RIGHT TRANSABDOMINAL ADRENALECTOMY	No

MSBOS - All records

Individual patient parameters (i.e., anemia, coagulopathy, transfusion risk) should be taken into consideration and may warrant an individual TSCR when not generally recommended.
June 22, 2023 **Sorted by CPT**

Primary CPT	Procedure Name	TSCR (Type and Screen) Needed?
61154	BURR HOLE(S) W/ EVAC/DRAIN HEMATOMA	No
61154	BURR HOLE(S) W/ EVAC/DRAIN HEMATOMA EXTRADURAL	No
61154	BURR HOLE(S) W/ EVAC/DRAIN HEMATOMA SUBDURAL	No
61312	CRANIECTOMY/ CRANIOTOMY EVACUATION HEMATOMA	Yes
61312	CRANIECTOMY/ CRANIOTOMY EVACUATION HEMATOMA EXTRADURAL OR SUBDURAL, SUPRATENTORIAL, SUPRATENTORIAL	Yes
61312	CRANIOTOMY FOR EVACUATION EXTRADURAL HEMATOMA SUPRATENTORIAL EXTRADURAL	Yes
61312	CRANIOTOMY FOR EVACUATION OF SUPRATENTORIAL SUBDURAL HEMATOMA	Yes
61313	CRANIECTOMY/ CRANIOTOMY EVACUATION HEMATOMA SUPRATENTORIAL, INTRACEREBRAL	No
61313	CRANIOTOMY FOR EVACUATION HEMATOMA	No
61313	CRANIOTOMY FOR EVACUATION HEMATOMA SUPRATENTORIAL INTRACEREBRAL	No
61343	CRANIECTOMY SUBOCCIPITAL W/ CERV LAMI FOR DECOMPRESS W/ DURAL GRAFT	No
61343	CRANIECTOMY SUBOCCIPITAL W/ CERV LAMI FOR DECOMPRESS W/ DURAL GRAFT	No
61343	CRANIECTOMY SUBOCCIPITAL WITH CERV LAMI FOR DECOMPRESS WITH DURAL GRAFT PEDIATRIC	No
61458	CRANIECTOMY FOR EXPLORATION / DECOMPRESSION CRANIAL NERVES	No
61510	CRANIECTOMY EXCISION TUMOR, TREPHINATION, BONE FLAP CRANIOTOMY, SUPRATENTORIAL, EXCEPT MENINGIOMA	No
61510	CRANIECTOMY EXCISION TUMOR, TREPHINATION, BONE FLAP CRANIOTOMY, SUPRATENTORIAL, EXCEPT MENINGIOMA	No
61510	CRANIECTOMY EXCISION TUMOR, TREPHINATION, BONE FLAP CRANIOTOMY, SUPRATENTORIAL, EXCEPT MENINGIOMA PEDIATRIC	No
61512	CRANIECTOMY, TREPHINATION, BONE FLAP CRANIOTOMY / EXCISE SUPRATENTORIAL MENINGIOMA	Yes
61512	CRANIECTOMY, TREPHINATION, BONE FLAP CRANIOTOMY / EXCISE SUPRATENTORIAL MENINGIOMA	Yes
61512	CRANIOTOMY FOR EXCISION SUPRATENTORIAL MENINGIOMA	Yes
61537	CRANI W/ ELEVATION BONE FLAP FOR TEMPORAL LOBECTOMY W/O INTRAOPERATIVE EC	No
61537	CRANI W/ ELEVATION BONE FLAP FOR TEMPORAL LOBECTOMY W/O INTRAOPERATIVE EC	No
61537	CRANIOTOMY W/ BONE FLAP FOR TEMPORAL LOBECTOMY W/O INTRAOPERATIVE EC	No
61624	PERCUTANEOUS TRANSCATHETER PERMANENT OCCLUSION OR EMBOLIZATION ANY METHOD CENTRAL NERVOUS SYSTEM	No
61624	TRANS CATH EMBOLIZATION CNS INTRASPINAL	No
61624	TRANSCATH EMBOLIZATION CNS INTRACRANIAL	No
61624	TRANSCATH EMBOLIZATION CNS INTRACRANIAL	No
61645	PERC ARTERIAL TRANSLUMINAL MECHANICAL THROMBECTOMY AND/OR INFUSION FOR THROMBOLYSIS, INTRACRANIAL, ANY METHOD INCL DIAG ANGIOGRAPHY, FLOURO GUIDANCE,CATH PLACEMENT/ INTRAPROC PHARM THROMBOLYTIC INJ	No
61711	ARTERIAL ANASTOMOSIS EXTRACRANIAL->INTRACRANIAL ARTERIES W/ CRANIOTOMY	No
61750	NAVIGATIONAL BIOPSY, ASPIRATION, OR EXCISION, WITH BURR HOLE(S), FOR INTRACRANIAL LESION	No
61750	STEREOTACTIC BIOPSY INTRACRANIAL LESION	No
61750	STEREOTACTIC BIOPSY INTRACRANIAL LESION	No
61760	STEREOTACTIC IMPLANT OF DEPTH ELECTRODES INTO CEREBRUM FOR SEIZURE MONITORING	No
61867	BURR HOLE W/ STEROTACTIC IMP NEUROSTIM LEAD SUBCORTICAL W/ INTRAOP RECORD 1ST ARRAY	No
61867	BURR HOLE W/ STEROTACTIC IMP NEUROSTIM LEAD SUBCORTICAL W/ INTRAOP RECORD 1ST ARRAY	No
61867	CRANIECTOMY W/ STEROTACTIC IMP NEUROSTIM LEAD SUBCORTICAL W/ INTRAOP RECORD 1ST ARRAY	No

MSBOS - All records

Individual patient parameters (i.e., anemia, coagulopathy, transfusion risk) should be taken into consideration and may warrant an individual TSCR when not generally recommended.
June 22, 2023 **Sorted by CPT**

Primary CPT	Procedure Name	TSCR (Type and Screen) Needed?
61867	TWIST DRILL BURR HOLE W/ STEREO IMPL NEUROSTIM ELEC SUBCORTICAL 1ST ARRAY W/ INTRAOP RECORDING	No
61885	INSERT VAGUS NERVE STIMULATOR GENERATOR, CONNECT TO SINGLE ELECTRODE LEAD ARRAY	No
61885	INSERTION OR REPLACEMENT GENERATOR NEUROSTIMULATOR CRANIAL	No
61885	INSERTION OR REPLACEMENT GENERATOR NEUROSTIMULATOR CRANIAL	No
61886	INSERT/REPLACE CRANIAL NEUROSTIMULOR GENERATOR/RECEIVER W/ CONNECT TO MULTI ELECTRODES	No
62165	NEUROENDOSCOPY INTRACRANIAL W/ EXCISION OF PITUITARY TUMOR	No
62165	NEUROENDOSCOPY INTRACRANIAL W/ EXCISION OF PITUITARY TUMOR TRANS-NASAL APPROACH	No
62165	NEUROENDOSCOPY INTRACRANIAL W/ EXCISION OF PITUITARY TUMOR TRANS-SPHENOIDAL APPROACH	No
62223	CREATION OF SHUNT; PEDIATRIC VENTRICULO-PERITONEAL, -PLEURAL, OTHER TERMINUS	No
62223	CREATION OF SHUNT; PEDIATRIC VENTRICULO-PERITONEAL, -PLEURAL, OTHER TERMINUS PEDIATRIC	No
62223	IMPLANT SHUNT VENTRICULOPERITONEAL	No
62223	IMPLANT SHUNT VENTRICULOPERITONEAL	No
62223	IMPLANT SHUNT VENTRICULOPERITONEAL PEDIATRIC	No
62223	NEURO ENDOSCOPIC CREATION OF VENTRICULO-PERITONEAL SHUNT ADULT	No
62230	REVISION SHUNT	No
62230	REVISION SHUNT LUMBO-PERITONEAL	No
62230	REVISION SHUNT SUBDURAL PERITONEAL	No
62230	REVISION SHUNT VENTRICULO-ATRIAL	No
62230	REVISION SHUNT VENTRICULOPERITONEAL	No
62230	REVISION SHUNT VENTRICULOPERITONEAL PEDS	No
62362	IMPLANT INTHRATHECAL PROGRAMMABLE PUMP WITH LEAD	No
62362	IMPLANT PROGRAMMABLE PAIN PUMP	No
62362	REPLACE PROGRAMMABLE PAIN PUMP	No
62362	REPLACE PROGRAMMABLE PAIN PUMP	No
62362	REPLACE PUMP INTHRATHECAL RESERVIOR	No
63005	DECOMPRESSION LAMINECTOMY LUMBAR LEVEL 1	No
63005	DECOMPRESSION LAMINECTOMY LUMBAR LEVEL 1	No
63005	DECOMPRESSION LAMINECTOMY LUMBAR LEVEL 1 OR 2 WITH C-ARM	No
63005	DECOMPRESSION LAMINECTOMY LUMBAR LEVEL 2	No
63005	LAMINECTOMY LUMBAR DECOMPRESSION WITH FUSION	No
63005	LAMINECTOMY LUMBAR LEVEL 1	No
63005	LAMINECTOMY LUMBAR LEVEL 2	No
63015	DECOMPRESSION LAMINECTOMY CERVICAL POSTERIOR LEVEL 3	No
63015	DECOMPRESSION LAMINECTOMY CERVICAL POSTERIOR LEVEL 3	No
63015	DECOMPRESSION LAMINECTOMY CERVICAL POSTERIOR LEVEL 4	No
63015	DECOMPRESSION LAMINECTOMY CERVICAL POSTERIOR LEVEL 5	No
63015	DISCECTOMY CERVICAL LEVEL 2	No

MSBOS - All records

Individual patient parameters (i.e., anemia, coagulopathy, transfusion risk) should be taken into consideration and may warrant an individual TSCR when not generally recommended.
June 22, 2023 **Sorted by CPT**

Primary CPT	Procedure Name	TSCR (Type and Screen) Needed?
63015	LAMINECTOMY CERVICAL LEVEL 3	No
63015	LAMINECTOMY CERVICAL POSTERIOR LEVEL 3	No
63015	LAMINECTOMY W/ DECOMPRESSION W/O DISCETOMY > 2 VERT SEG CERV SPINE	No
63015	LAMINECTOMY W/ EXPLORATION W/O DISCECTOMY > 2 VERT SEG CERV SPINE	No
63030	DISCOTOMY LUMBAR LEVEL 1	No
63030	LAMINECTOMY DISCECTOMY LUMBAR LEVEL 1	No
63030	LAMINOTOMY W/ DECOMPRESSION OF NERVE ROOT(S) PARTIAL FACETECTOMY FORAMINOTOMY AND/OR EXCISION OF HERNIATED INTERVERTEBRAL DISC 1 INTERSPACE LUMBAR	No
63030	LAMINOTOMY W/ DECOMPRESSION OF NERVE ROOT(S) PARTIAL FACETECTOMY FORAMINOTOMY AND/OR EXCISION OF HERNIATED INTERVERTEBRAL DISC 1 INTERSPACE LUMBAR	No
63030	OPEN HEMILAMINECTOMY FOR DECOMPRESSION NERVE ROOT 1 INTERSPACE LUMBAR	No
63042	LAMINOTOMY LUMBAR LEVEL 1	No
63042	OPEN LAMINOTOMY LUMBAR W/ DECOMPRESS W/ PART EXC OF DISC 1 INTRSPACE	No
63042	RE-EXPLORATION LAMINOTOMY DISCECTOMY LUMBAR LEVEL 1	No
63042	RE-EXPLORATION LAMINOTOMY DISCECTOMY LUMBAR LEVEL 1	No
63042	RE-EXPLORATION LUMBAR LAMINOTOMY (HEMILAMINECTOMY),W/DECOMPRESSION OF NERVE ROOT(S),INCLUDING PARTIAL FACETECTOMY,FORAMINOTOMY AND/OR EXCISION OF HERNIATED INTERVERTEBRAL DISC SINGLE INTERSPACE	No
63045	DECOMPRESSION LAMINECTOMY CERVICAL FACETECTOMY AND FORAMINOTOMY, SINGLE VERT SEGMENT LEVEL 6	No
63045	DECOMPRESSION LAMINECTOMY CERVICAL REEXPLORATION W/ PARTIAL FACETECTOMY, FORAMINOTOMY AND/OR EXCISION HERNIATED INTERVERTEBRAL DISC LEVEL 3	No
63045	DECOMPRESSION LAMINECTOMY CERVICAL, FACETECTOMY AND FORAMINOTOMY	No
63045	DECOMPRESSION LAMINECTOMY CERVICAL, FACETECTOMY AND FORAMINOTOMY, SINGLE VERT SEGMENT LEVEL 2	No
63045	DECOMPRESSION LAMINECTOMY CERVICAL, FACETECTOMY AND FORAMINOTOMY, SINGLE VERT SEGMENT LEVEL 3	No
63045	DECOMPRESSION LAMINECTOMY CERVICAL, FACETECTOMY AND FORAMINOTOMY, SINGLE VERT SEGMENT LEVEL 4	No
63045	DECOMPRESSION LAMINECTOMY CERVICAL, FACETECTOMY AND FORAMINOTOMY, SINGLE VERT SEGMENT LEVEL 5	No
63045	DECOMPRESSION LAMINECTOMY CERVICAL, FACETECTOMY AND FORAMINOTOMY, SINGLE VERT SEGMENT LEVEL 7	No
63045	DECOMPRESSION LAMINECTOMY CERVICAL, FACETECTOMY AND FORAMINOTOMY, SPINAL OR LATERAL RECESS STENOSIS LEVEL 1	No
63046	DECOMPRESSION LAMINECTOMY THORACIC, FACETECTOMY AND FORAMINOTOMY, SPINAL OR LATERAL RECESS STENOSIS	No
63046	DECOMPRESSION LAMINECTOMY THORACIC, FACETECTOMY AND FORAMINOTOMY, SPINAL OR LATERAL RECESS STENOSIS SINGLE LEVEL 1	No
63046	DECOMPRESSION LAMINECTOMY THORACIC, FACETECTOMY AND FORAMINOTOMY, SPINAL OR LATERAL RECESS STENOSIS SINGLE LEVEL 10	No
63046	DECOMPRESSION LAMINECTOMY THORACIC, FACETECTOMY AND FORAMINOTOMY, SPINAL OR LATERAL RECESS STENOSIS SINGLE LEVEL 12	No
63046	DECOMPRESSION LAMINECTOMY THORACIC, FACETECTOMY AND FORAMINOTOMY, SPINAL OR LATERAL RECESS STENOSIS SINGLE LEVEL 2	No

MSBOS - All records

Individual patient parameters (i.e., anemia, coagulopathy, transfusion risk) should be taken into consideration and may warrant an individual TSCR when not generally recommended.
June 22, 2023 **Sorted by CPT**

Primary CPT	Procedure Name	TSCR (Type and Screen) Needed?
63046	DECOMPRESSION LAMINECTOMY THORACIC, FACETECTOMY AND FORAMINOTOMY, SPINAL OR LATERAL RECESS STENOSIS SINGLE LEVEL 6	No
63046	DECOMPRESSION LAMINECTOMY THORACIC, FACETECTOMY AND FORAMINOTOMY, SPINAL OR LATERAL RECESS STENOSIS SINGLE LEVEL 8	No
63046	DECOMPRESSION LAMINECTOMY THORACIC, FACETECTOMY AND FORAMINOTOMY, SPINAL OR LATERAL RECESS STENOSIS SINGLE LEVEL 9	No
63047	DECOMPRESSION LAMINECTOMY LUMBAR POSTERIOR LEVEL 1	No
63047	DECOMPRESSION LAMINECTOMY LUMBAR POSTERIOR LEVEL 1	No
63047	DECOMPRESSION LAMINECTOMY LUMBAR POSTERIOR LEVEL 1 WITH C ARM	No
63047	DECOMPRESSION SPINAL CORD LAMINECTOMY LUMBAR ANTERIOR LEVEL 1	No
63047	DECOMPRESSION SPINAL CORD LAMINECTOMY LUMBAR LEVEL 1	No
63047	LAMINECTOMY DECOMPRESSION FACETECTOMY AND FORAMINOTOMY	No
63047	MIS DECOMPRESSION LAMINECTOMY; DISCECTOMY; LUMBAR LEVEL 1	No
63051	C-LAMINOPLASTY W/GRAFT/PLATE	No
63051	C-LAMINOPLASTY W/GRAFT/PLATE	No
63051	LUMBAR LAMINECTOMY W/ RECON OF THE POST BONY ELEMENTS	No
63052	LAM FACETEC/FORAMOT DRG ARTHRD LUMBAR 1 VRT SGM	No
63267	LAMINECTOMY FOR EXCISION / EVACUATION OF EXTRADURAL INTRASPINAL LESION NON-NEOPLASM LUMBAR SPINE	No
63650	IMPLANTATION ELECTRODE ARRAY, NEUROSTIMULATOR, EPIDURAL, PERCUTANEOUS	No
63650	IMPLANTATION ELECTRODE ARRAY, NEUROSTIMULATOR, EPIDURAL, PERCUTANEOUS	No
63650	PERCUTANEOUS IMPLANT LEAD NEUROSTIM EPIDURAL TRIAL	No
63685	INSERTION SPINAL CORD STIMULATOR GENERATOR	No
63685	INSERTION SPINAL CORD STIMULATOR GENERATOR	No
63685	INSERTION STIMULATOR TRIAL SPINAL CORD	No
63685	REPLACE STIMULATOR GENERATOR PROGRAMMABLE	No
64561	PERCUTANEOUS IMPLANTATION OF NEUROSTIMULATOR ECLECTRODE ARRAY SACRAL NERVE W/IMAGE GUIDANCE	No
64581	IMPLANT STIMULATOR LEAD(S) BLADDER INCISIONAL APPROACH	No
64581	INTERSTIM PROCEDURE STAGE 1	No
64581	INTERSTIM PROCEDURE STAGE 1	No
64581	TRANSFORAMINAL PLCMNT VIA INCISION NEUROSTIM ELECTRODES SACRAL	No
64582	OPEN IMPLTJ HPGLSL NRV NSTIM RA PG&RESPIR SENSOR	No
64585	REMOVE PERIPHERAL NEUROSTIMULATOR LEADS LOWER EXTREMITY	No
64585	REMOVE PERIPHERAL NEUROSTIMULATOR LEADS UPPER EXTREMITY	No
64585	REMOVE STIMULATOR LEAD BLADDER	No
64585	REVISION OR REMOVAL PERIPHERAL NEUROSTIMULATOR ELECTRODES	No
64585	REVISION OR REMOVAL PERIPHERAL NEUROSTIMULATOR ELECTRODES	No
64590	INSERT PERIPHERAL NEUROSTIMULATOR PULSE GENERATOR LOWER EXTREMITY	No

MSBOS - All records

Individual patient parameters (i.e., anemia, coagulopathy, transfusion risk) should be taken into consideration and may warrant an individual TSCR when not generally recommended.

June 22, 2023 **Sorted by CPT**

Primary CPT	Procedure Name	TSCR (Type and Screen) Needed?
64590	INSERTION GASTRIC NEUROSTIMULATOR GENERATOR	No
64590	INSERTION PERIPHERAL NEURO STIMULATOR PULSE GENERATOR	No
64590	INSERTION STIMULATOR GENERATOR BLADDER	No
64590	INSERTION STIMULATOR GENERATOR BLADDER	No
64590	INTERSTIM PROCEDURE STAGE 2	No
64590	REPLACE STIMULATOR GENERATOR BLADDER	No
64590	REPLACEMENT GASTRIC NEUROSTIMULATOR GENERATOR	No
64590	REPLACEMENT PERIPHERAL NEUROSTIMULATOR PULSE GENERATOR	No
64642	CHEMODENERVATION OF ONE LOWER EXTREMITY; 1-4 MUSCLE(S)	No
64642	CHEMODENERVATION OF ONE LOWER EXTREMITY; 1-4 MUSCLE(S)	No
64642	CHEMODENERVATION OF ONE UPPER EXTREMITY; 1-4 MUSCLE(S)	No
64718	DECOMPRESSION NERVE ULNAR ELBOW	No
64718	DECOMPRESSION NERVE ULNAR ELBOW	No
64718	NEUROPLASTY ULNAR NERVE AT ELBOW	No
64718	TRANSPOSITION NERVE ULNAR	No
64721	DECOMPRESSION NERVE MEDIAN CARPAL TUNNEL	No
64721	DECOMPRESSION NERVE MEDIAN CARPAL TUNNEL	No
64721	NEUROPLASTY MEDIAN NERVE AT CARPAL TUNNEL	No
64999	ANASTOMOSIS FACIAL TO MASSETERIC NERVES W/ CRANIOTOMY	No
64999	CRANIECTOMY OR CRANIOTOMY FOR INSERTION OF AUDITORY BRAIN STEM IMPLANT	No
64999	DESTRUCTION BY RFA/ KNEE/ COOL METHOD	No
64999	DESTRUCTION BY RFA/ SACROILIAC JOINT/ BILATERAL/ COOL METHOD	No
64999	DESTRUCTION BY RFA/ SACROILIAC JOINT/ UNILATERAL/ COOL METHOD	No
64999	ENCEPHALODURANGIOMYANGIOSIS PROCEDURE	No
64999	ENDOSCOPIC RESECTION OF AN INTRACRANIAL/ INTRADURAL TUMOR	No
64999	ENDOSCOPIC RESECTION TRANSNASAL SKULL BASE TUMOR	No
64999	ENDOSCOPIC TRANSNASAL INTRACRANIAL BIOPSY	No
64999	NERVE BLOCK,UNSPECIFIED	No
64999	NERVE DESTRUCTION,UNSPECIFIED	No
64999	NERVE TRANSFER UPPER EXTREMITY	No
64999	NEUROENDOSCOPIC REPAIR OF CSF LEAK WITH FLAP	No
64999	NEUROENDOSCOPY ANTERIOR CRANIOFACIAL RESECTION	No
64999	PLACEMENT OF INTRACRANIAL PRESSURE MONITOR W/ CRANI	No
64999	REMOVAL OF SUBDURAL EEG ELECTRODES	No
64999	REMOVAL OF SUBDURAL EEG ELECTRODES	No
64999	RFA DESTRUCTION SUPRASCAPULAR NERVE	No
64999	TRANSMASTOID REPAIR ENCEPHALOCELE	No

MSBOS - All records

Individual patient parameters (i.e., anemia, coagulopathy, transfusion risk) should be taken into consideration and may warrant an individual TSCR when not generally recommended.
June 22, 2023 **Sorted by CPT**

Primary CPT	Procedure Name	TSCR (Type and Screen) Needed?
65730	KERATOPLASTY, PENETRATING CORNEAL TRANSPLANT EXCEPT APHAKIA OR PSUEDOAPHAKIA	No
65756	KERATOPLASTY ENDOTHELIAL DESCEMET MEMBRANE (DMEK)	No
65756	KERATOPLASTY ENDOTHELIAL DESCEMET MEMBRANE (DMEK)	No
65756	KERATOPLASTY ENDOTHELIAL DESCEMET STRIPPING (DSAEK)	No
66821	LASER POSTERIOR CAPSULOTOMY BILATERAL~ YAG	No
66821	LASER POSTERIOR CAPSULOTOMY UNILATERAL~ YAG	No
66821	LASER POSTERIOR CAPSULOTOMY UNILATERAL~ YAG	No
66982	COMPLEX EXTRACAPSULAR CATARACT REMOVAL W/ INS OF IOL W/O ENDOSCOPIC CYCLOPHOTOCOAGULATION PEDS	No
66982	EXTRACAPSULAR CATARACT REMOVAL W/ INSERTION OF IOL ON AMBLYOPIC W/O ENDOSCOPIC CYCLOPHOTOCOAGULATION PEDS	No
66982	REMOVAL EXTRACAPSULAR CATARACT W/INSERTION IOL PROSTHESIS COMPLEX W/O ENDOSCOPIC CYCLOPHOTOCOAGULATION	No
66982	REMOVAL EXTRACAPSULAR CATARACT W/INSERTION IOL PROSTHESIS COMPLEX W/O ENDOSCOPIC CYCLOPHOTOCOAGULATION	No
66984	EXTRACAPSULAR CATARACT REMOVAL W/ INSERTION OF IOL ONE STAGE W/O ENDOSCOPIC CYCLOPHOTOCOAGULATION PEDS	No
66984	EXTRACAPSULAR CATARACT REMOVAL W/INS IOL MANUAL/MECHANICAL W/O ENDOSCOPIC CYCLOPHOTOCOAGULATION	No
66984	PHACOEMULSIFICATION CATARACT ANTERIOR IMPLANT INTRAOCULAR LENS W/O ENDOSCOPIC CYCLOPHOTOCOAGULATION	No
66984	PHACOEMULSIFICATION CATARACT IMPLANT INTRAOCULAR LENS W/O ENDOSCOPIC CYCLOPHOTOCOAGULATION	No
66984	PHACOEMULSIFICATION CATARACT IMPLANT INTRAOCULAR LENS W/O ENDOSCOPIC CYCLOPHOTOCOAGULATION	No
67218	DESTROY LOC LESION RETINA W/ RADIATION IMPLANT	No
67311	REPAIR STRABISMUS 1 HORIZONTAL MUSCLE	No
67312	REPAIR STRABISMUS 2 HORIZONTAL MUSCLES	No
67314	STRABISMUS SURGERY, RECESSION OR RESECTION PROCEDURE; 1 VERTICAL MUSCLE	No
67908	REPAIR BLEPHAROPTOSIS CONJUNCTIVO-TARSO LEVATOR RESECTION	No
67966	EXCISION & REPAIR OF > 1/4 OF EYELID W/GRAFT	No
69436	TYMPANOSTOMY W/VENT TUBES GEN ANES	No
69620	MYRINGOPLASTY	No
69620	MYRINGOPLASTY	No
69620	MYRINGOPLASTY PEDIATRIC	No
69631	TYMPANOPLASTY	No
69930	IMPLANT COCHLEAR DEVICE W/ MASTOIDECTOMY	No
69930	IMPLANT COCHLEAR DEVICE W/ MASTOIDECTOMY	No
69930	IMPLANT COCHLEAR DEVICE W/ MASTOIDECTOMY PEDS	No
69930	IMPLANT COCHLEAR DEVICE W/O MASTOIDECTOMY	No
75625	AORTOGRAM ABDOMINAL	No
75710	ANGIOGRAM EXTREMITY UNILATERAL RADIOLOGICAL	No
75710	ANGIOGRAM EXTREMITY UNILATERAL RADIOLOGICAL	No
75710	ANGIOGRAM FEMORAL	No

MSBOS - All records

Individual patient parameters (i.e., anemia, coagulopathy, transfusion risk) should be taken into consideration and may warrant an individual TSCR when not generally recommended.

June 22, 2023 **Sorted by CPT**

Primary CPT	Procedure Name	TSCR (Type and Screen) Needed?
75710	ARTERIOGRAM POPLITEAL	No
76998	S&I INTRAOPERATIVE ULTRASOUND GUIDANCE	No
92018	EXAM UNDER ANESTHESIA EYE COMPLETE	No
92136	OPHTHALMIC BIOMETRY BY PARTIAL COHERENCE INTERFEROMETRY W/INTRAOCULAR LENS POWER CALCULATION	No
92652	AUDITORY EVOKED POTENTIALS; FOR THRESHOLD ESTIMATION AT MULTIPLE FREQUENCIES,W/INTERPRETATION AND REPORT	No
97605	APPLICATION WOUND VAC ABDOMEN TOTAL WOUND SURFACE LESS THAN 50 SQ CENTIMETERS	No
97605	APPLICATION WOUND VAC BACK TOTAL WOUND SURFACE LESS THAN 50 SQ CENTIMETERS	No
97605	APPLICATION WOUND VAC EXTREMITY LOWER TOTAL WOUND SURFACE LESS THAN 50 SQ CENTIMETERS	No
97605	NEGATIVE PRESSURE WOUND THERAPY TRUNK TOTAL WOUND SURFACE AREA LESS THEN OR EQUAL TO 50 SQ CM	No
97605	NEGATIVE PRESSURE WOUND THERAPY TRUNK TOTAL WOUND SURFACE AREA LESS THEN OR EQUAL TO 50 SQ CM	No
97605	REMOVAL WOUND VAC EXTREMITY LOWER TOTAL WOUND SURFACE LESS THAN 50 SQ CENTIMETERS	No
S2118	APPLICATION WOUND VAC CHEST TOTAL WOUND SURFACE LESS THAN 50 SQ CENTIMETERS	No
