# Supplementary material for: A maximum surgical blood ordering schedule: Does it add value?
Source: Vox Sang. 2025 Feb 25;120(4):411–8. doi: 10.1111/vox.13804 (PMC12017947; doi:10.1111/vox.13804)
Supplement: Supplementary file 3 — Data S3. Supporting information. [file VOX-120-411-s001.docx]

**Maximum Surgical Blood Ordering Schedule (MSBOS)**

**Project A3**

**Project Sponsor**; Dr. Mark Taylor. **Process Owners**: Dr. Deborah Benzil,

Dr. NurJehan Quraishy **Project Lead**: Debbie Tolich

**Project Team**: Ralph Luther, Dr. Ken Cummings, Dr. Moises Auron, Dan Lallo, Jessica Calo, Michael Reese, Noreen Flowers

**1. Background**

Cleveland clinic performs more than 200,000 surgeries and procedures every year. At present, the pre-operative testing for nearly all procedures requires a Type and Screen and "Confirm ABO" test. In reality, fewer than X % (pending data) of cases require transfusion during or immediately following surgery and even smaller number on emergent basis. It is well established that eliminating unnecessary testing such as Type and Screen can reduce costs without a negative impact on patient safety and quality of care. In addition to the labor, supplies, transport and laboratory costs associated with this testing, there is also the potential for delayed starts and patient discomfort (from multiple sticks).

Critical support must be established from anesthesia, the chairs of surgical departments, and the staff of pre-operative services in order to achieve consensus on case designation and critical co-morbidities (eg. age, pre-operative Hgb, risk factors). Anesthesia transfusion documentation will be challenging because formats and definitions are not aligned with Business Intelligence. Ordering providers must have access to link to the process within EPIC pre-op blood orders.

Currently:

- Nearly all surgeries have Type and Screen as "default" for pre-operative testing.
- Inefficiency: time spent allocating/issuing units and then returning unused units. Allocated units are not available for other patients.
- Data: number of Type and Screens drawn in pre-operative pateints, cost of time, supplies, laboratory processing, estimation of delays related to Type and Screen draws, number of intraoperative transfusions given
- Blood expenses
- Tech time/ labor costs

**2. Goal Statement & Targets**

Eliminate 90% of unnecessary Type and Screen/Confirm ABO tests through an automated, stratified risk protocol and EPIC automation.

- Develop a consensus risk stratification for the most common procedures performed across the enterprise
- Track compliance after roll out, reduction of tests, and associated cost savings
- Track complications resulting from NOT having a Type and Screen in a patient who required transfusion intraoperatively

**3. Scope**

**Out of Scope:** Pediatric, Obstetric, and emergency surgical procedures, patients with atypical antibodies, Cleveland Clinic Abu Dhabi (CCAD), Cleveland Clinic London (CCL).

**4. Plan (Components: Responsible Team/Anticipated Date of completion)**

1. Initiate connection with EPIC team: June 2021/Completed
2. Communication Plan: Jan 2023/Completed
3. Meeting D Benzil, K Mahesheshwari, J Owens (where transfusions occur): June 2021/Completed
4. Data collection Top Surgeries: June 2021/Completed
5. Regional and MC separate
6. 75% coverage of cases
7. Transfusion rate/case type/pending
8. Avg estimated blood loss/pending
9. Review top cases with institutes and anesthesia for TSCR need: DB lead once data available/pending data/Completed
10. Forward literature to group: June 2021/Completed
11. Follow up meeting 3-4 weeks: June 2021/Completed
12. Combine Project Plan Templates: June 2021/Completed

***See project task tracker**

**5. POST IMPLEMENTATION**

1. Audit compliance and feedback 2-3 weeks after launch.
2. Compliance data at short, regular intervals until stable
3. Report any patient safety events at short regular intervals until stable
4. Assess need for intervention to enhance compliance
5. Compare 6-12 month data prior and post implementation (report build in EPIC?)
6. Assess financial impact

**6. References**

Dexter *F. et al.* Systematic Criteria for Type and Screen Based on Procedure’s probability of Erythrocyte Transfusion, Anesthesiology, 2012, 116;4: 768-771

Sutherland A. Evaluating the Benefits of an Updated Blood Ordering Process

Clinic Laboratory Science, 2017, 30;4:219-223

Frank S.F. et al. Optimizing Preoperative Blood Ordering with Data Acquired from an Anesthesia Information Management System, Anesthesiology, 2013, 118;6:1286-1297

Azizgolshani NM, Porter ED, Fay KA, Dunbar NM, Hasson RM, Millington TM, Finley DJ, Phillips JD. Preoperative Type and Screen is Unnecessary in Elective Anatomic Lung Resection and Esophagectomy. J Surg Res. 2020 Nov;255:411-419.

Christopher ZK, Bruce MR, Reynolds EG, Spangehl MJ, Bingham JS, Kraus MB. Routine Type and Screens Are Unnecessary for Primary Total Hip and Knee Arthroplasties at an Academic Hospital. Arthroplast Today. 2020 Nov 26;6(4):941-944.

Stangenberg L, Curran T, Shuja F, Rosenberg R, Mahmood F, Schermerhorn ML. Development of a risk prediction model for transfusion in carotid endarterectomy and demonstration of cost-saving potential by avoidance of "type and screen". J Vasc Surg. 2016 Dec;64(6):1711-1718.

van Klei WA, Moons KG, Leyssius AT, Knape JT, Rutten CL, Grobbee DE. A reduction in type and screen: preoperative prediction of RBC transfusions in surgery procedures with intermediate transfusion risks. Br J Anaesth. 2001 Aug;87(2):250-7.

Prichard RS, O'Keefe M, McLaughlin R, Malone C, Sweeney KJ, Kerin MJ. A study of pre-operative type and screen in breast surgery: improved efficiency and cost saving. Ir J Med Sci. 2011 Jun;180(2):513-6.

Gupta N, Visagie M, Kajstura TJ, Han M, Trock B, Gehrie EA, Frank SM, Bivalacqua TJ. Reducing preoperative blood orders and costs for radical prostatectomy. J Comp Eff Res. 2020 Feb;9(3):219-226.
